# Quantifying the failure modes of current one-step retrosynthesis models

**DOI:** 10.1039/d6sc01323f

**Published:** 2026-06-04

**Authors:** Suong B. A. Tran, Jihye Roh, Connor W. Coley

**Affiliations:** a Department of Chemistry, Massachusetts Institute of Technology 77 Massachusetts Avenue Cambridge MA 02139 USA; b Department of Chemical Engineering, Massachusetts Institute of Technology 77 Massachusetts Avenue Cambridge MA 02139 USA ccoley@mit.edu; c Department of Electrical Engineering and Computer Science, Massachusetts Institute of Technology 77 Massachusetts Avenue Cambridge MA 02139 USA

## Abstract

Computer-aided synthesis planning (CASP) automates retrosynthetic analysis, generally by recursively applying one-step retrosynthesis models within multistep search algorithms to simplify a target molecule into commercially available starting materials. Despite their utility, these tools often fail to recover literature-reported pathways. Such failures arise from two causes: either (i) the literature-reported precursor is not proposed at all or (ii) it is proposed but ranked too low to be discovered during a multistep search. In this work, we quantify the challenges that data-driven one-step retrosynthesis models face in reproducing literature-reported precursors. We first evaluate model performance using standard top-*k* exact-match accuracy and stratify this accuracy by product and reaction complexity, demonstrating a decrease in performance with increasing complexity. This decline is accompanied by a systematic underprediction of the number of reacting atoms and changing rings, indicating a bias toward simpler transformations, even when complex examples are included in the training data. To gain deeper insights into failure modes, we evaluate models with complementary metrics that account for incorrect stereochemistry, leaving groups, and multi-stage reactions. Overall, our work provides a quantitative analysis of how one-step retrosynthesis models fail to capture literature-reported reactions, highlighting opportunities for improving future models and providing guidance on using model predictions more effectively in prospective synthesis planning.

## Abstract 2

This work quantifies the failure modes of one-step retrosynthesis models in recovering literature-reported precursors, using complementary accuracy metrics and stratifying accuracy across product and reaction complexity.

## Graphical abstract

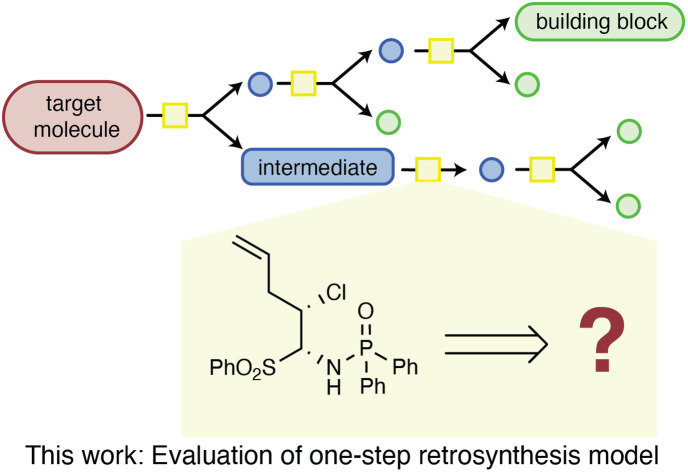

## 1 Introduction

The synthesis of structurally and synthetically complex molecules often poses significant challenges, requiring substantial time, resources, and chemical expertise. Retrosynthetic analysis provides the conceptual basis for computer-aided synthesis planning (CASP) to facilitate this process.^1–4^ These systems generally formulate multistep planning as a search problem in which route construction proceeds through a cycle of expansion and selection. During expansion, a one-step retrosynthesis model aims to propose candidate reactants (precursors) for a target molecule. The selection policy then evaluates these candidates and chooses which precursor to pursue next in the search. If the expansion step fails to propose the literature-reported precursors, then the search will fail to find the literature-reported route regardless of the selection policy. Therefore, we focus on the expansion step to characterize the failure modes of one-step retrosynthesis models in this work.

Many types of models have been developed to carry out one-step retrosynthesis prediction. Expert methods rely on encoded reaction rules, where chemists define transformations for known reaction types.^2,4,5^ Data-driven approaches try to infer such patterns from reaction data directly, including template-based and template-free methods. Template-based models generally treat retrosynthesis as a classification problem, predicting the most likely algorithmically-extracted reaction template for a given product.^6–9^ Template-free models, in contrast, learn reaction patterns directly from molecular representations such as graphs or sequences without making use of symbolic templates.^10–13^

Evaluation of one-step models has largely relied on top-*k* exact-match accuracy, which provides an objective, quantitative measure of whether the experimentally reported precursors appear within a model's top-*k* suggestions. Variations of this metric have been introduced to capture different categories of incorrect predictions. Stereochemistry-agnostic accuracy, for example, accounts for incorrect stereochemistry.^8,14^ MaxFrag accuracy^10^ evaluates predictions based only on the largest reactant fragment; while this relaxation was introduced to address ambiguity in agents, it can erroneously classify predictions as correct even when smaller fragments are incorrectly specified, including cases where essential atoms are missing for product formation (Fig. S6B).

Retrosynthesis is inherently a one-to-many problem, in which a single product can arise from multiple sets of reactants. Accordingly, metrics such as the Tanimoto similarity between the molecular fingerprints of the predicted and recorded precursor sets, which quantifies structural similarity, and “halogen-agnostic” accuracy, which assumes halogen interchangeability, have been used to evaluate alternate potentially-valid pathways.^14^ In addition to structural matching criteria, there is round-trip accuracy,^15^ which evaluates whether a product-prediction model can regenerate the product from the predicted precursors. The ChemCensor score^16^ offers another approach, assigning a confidence level (0–5) by comparing a reaction center and functional group context against documented synthetic precedents. However, they remain coarse approximations of experimental feasibility as they rely on predictive models or simple heuristics.

Evaluation of a multi-step pathway search is even more fraught, as the one-to-many problem is exacerbated by the even larger number of chemically plausible answers.^17^ Existing benchmarks typically report metrics that measure search success and efficiency (*e.g.*, solve rate under varying computational budget), or characterize properties of the pathways found (*e.g.*, the number or diversity of pathways and the number of reactions per pathway).^17–19^ However, as with one-step retrosynthesis, recovery of literature-reported routes provides the only objective evidence available for experimental feasibility, short of actual experimental execution.^20–22^ In practice, CASP tools often fail to reproduce these literature-reported routes. Such failures arise from two reasons: (1) the reported precursor(s) is not generated at all, or (2) it is generated but ranked too low to be selected during a multistep search.

**Fig. 1:**
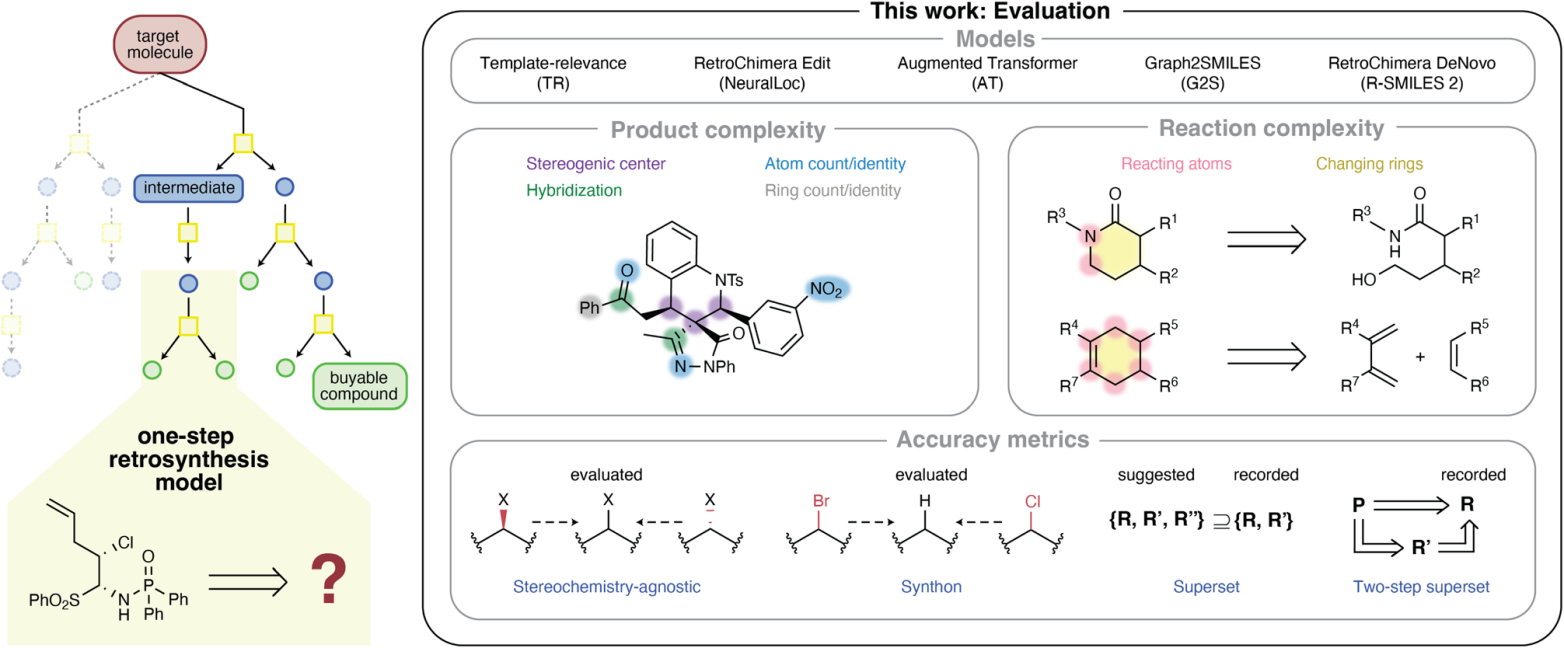
Overview of our evaluation of selected one-step retrosynthesis models: product complexity is quantified using chemical information and machine-learning metrics. Reaction complexity considers structural changes, including reacting atoms and changing rings. Prediction accuracy is measured with complementary metrics, including stereochemistry-agnostic, synthon, superset, and two-step superset, allowing quantified identification of models' failure modes.

In this work, we present a quantitative analysis of the failure modes of data-driven one-step retrosynthesis models in recovering literature-reported precursor(s) (Fig. 1). Five representative template-based and template-free models are included in the evaluation. We examine how performance decreases with increasing product and reaction complexity, stratifying the models' performance in order to quantify this anecdotal trend. Through this analysis, we demonstrate that current models systematically underpredict the number of reacting atoms and fail to capture ring transformations. We also include complementary metrics beyond conventional top-*k* exact-match accuracy that account for incorrect stereochemistry, choice of different leaving groups, and the ambiguity of single-step *versus* two-step reactions to show the contribution of each failure mode to each model's performance. Our analysis provides a more nuanced understanding of one-step model performance, informing both future model development and for the use of model suggestions in synthesis planning.

## 2 Methods

### 2.1 Models

In this work, we select representative models from both template-based and template-free one-step retrosynthesis approaches. For the template-based approaches, we consider two models: (1) the Template-relevance (TR) model,^7,23^ which uses a feedforward neural network to prioritize reaction templates by their likelihood of applying to a given product, and (2) NeuralLoc,^24^ which encodes templates as graphs to share information across related templates and uses atom-level localization to resolve multiple product matches. The template-free approaches include the following three models: (1) Augmented Transformer^10^ (AT), a SMILES-to-SMILES transformer enhanced with product SMILES augmentation, (2) Graph2SMILES^13^ (G2S), which encodes the molecular graph structure of the product and decodes the SMILES strings of reactants, and (3) R-SMILES 2, a sequence-to-sequence model that builds on the root-aligned SMILES representation^11^ to make product and reactant strings more directly aligned, and modernizes the transformer backbone with techniques from recent large language models.^24^ Details about each model, training procedure, and the choice of models used in this work are provided in Section S3.

### 2.2 Datasets

We use the following three reaction datasets to evaluate the performance of one-step retrosynthesis models: USPTO,^25^ Pistachio (2025Q2 version),^26^ and a subset of CAS covering reactions from 2010 to 2015.^27^ Our preprocessing procedure includes deduplication, validation, and standardization of reaction SMILES (Section S2.1).

To better assess the performance of models in generalizing to reactions originating from distinct publications,^28^ we perform our analysis using a document-based split, splitting the reactions into training (75%), validation (5%), and test (20%) sets. In the document-based split, all reactions reported in the same document are assigned to the same data partition, but are otherwise divided randomly (Table S5). We ensure that the reaction complexity distributions of the train and test sets are closely matched (Section S2.4). The specific definition of reaction complexity is provided in Section 2.3. We additionally report the top-*k* exact-match accuracy for a random split (Fig. S8). Our analyses focus on internal dataset evaluation, where models are evaluated within the same dataset. This approach minimizes variations arising from dataset-specific bookkeeping conventions that can otherwise confound accuracy statistics (Section S2.3).

### 2.3 Complexity metrics

We analyze model performance in relation to reaction and product complexity. Product complexity metrics are defined as those computed solely from the product SMILES, and reaction complexity metrics as those computed from the reaction SMILES (*i.e.*, considering both the reactants and products). For product complexity, we adopt the Böttcher score, Spacial Score (SPS), NPScore, and SAScore.^29–32^ The overall product complexity is calculated as the average of these four normalized metrics, each linearly scaled based on the minimum and maximum values specific to its dataset (Section S1.1).

For reaction complexity, we quantify the number of reacting atoms and the number of changed rings between reactants and products (Fig. S1). A reacting atom is defined as any product atom that exhibits a change in formal charge, neighboring atoms, or bond types compared to its corresponding atom in the reactant. The number of changing rings is quantified by comparing fully-mapped rings in both reactants and products. This metric quantifies instances of ring formation, cleavage, and rearrangement. Such changes are identified through atom mapping,^33^ which may introduce minor confounding errors due to its own imperfections (Section S5.3.2).

### 2.4 Evaluation metrics

Each one-step retrosynthesis model generates a ranked list of candidates for a given product, where each candidate consists of one or more reactant molecules. We define the following accuracy metrics to quantify how different failure modes contribute to overall performance.

#### 2.4.1 Exact-match accuracy

A prediction is correct at top-*k* by exact-match accuracy if at least one candidate in the top-*k* predictions exactly matches the recorded precursor(s).

#### 2.4.2 Stereochemistry-agnostic accuracy

A prediction is considered correct at top-*k* by stereochemistry-agnostic accuracy if at least one candidate within the top-*k* set of unique candidates (after removing stereochemistry from all predictions) matches the recorded precursor(s) with stereochemistry removed. Previous “stereo-agnostic” metrics consider only *R*/*S* configurations; we extend this definition to include *E*/*Z* stereochemistry.^8,14^

#### 2.4.3 Synthon accuracy

A prediction is considered correct at top-*k* by synthon accuracy if at least one synthon within the top-*k* set of unique synthons matches the recorded synthon. A synthon is obtained from a precursor by removing all atoms and bonds that are not in the product. The term synthon was first introduced by Corey, but its usage within the chemical community is not consistent.^34^ Here, we adopt a definition of synthons closely aligned with Corey's original concept^35^ (Section S4). This accuracy extends the “halogen-agnostic” accuracy metric used by Srikar-Tadanki *et al.*^14^ to also account for other types of leaving groups. Although the exact identity of the leaving groups is essential for determining reactivity, this evaluation captures the core logic of the transformation when assessing one-step retrosynthesis models' ability to propose correct retrosynthetic disconnections.

#### 2.4.4 Superset accuracy

A prediction is correct at top-*k* by superset accuracy if at least one candidate in the top-*k* predictions is a superset of the recorded precursor(s).

#### 2.4.5 Two-step superset accuracy

A prediction is considered correct at top-*k* by two-step superset accuracy if at least one candidate in the top-*k* predictions of the first pass (i) is correct by superset accuracy or (ii) its second-pass output, when combined with the smaller first-pass precursor(s), is correct by superset accuracy. To construct the second pass predictions, we treat the first-pass candidates as input products and generate an additional set of candidates. For candidates with multiple precursor molecules, we take only the one with the greatest number of heavy atoms.

We note that these metrics are complementary rather than mutually exclusive, since a single prediction may satisfy multiple criteria depending on how it differs from the recorded precursors. Details on the implementation of each metric are provided in Section S4.

## 3 Results

### 3.1 Models' performance declines with increasing product and reaction complexity

**Fig. 2:**
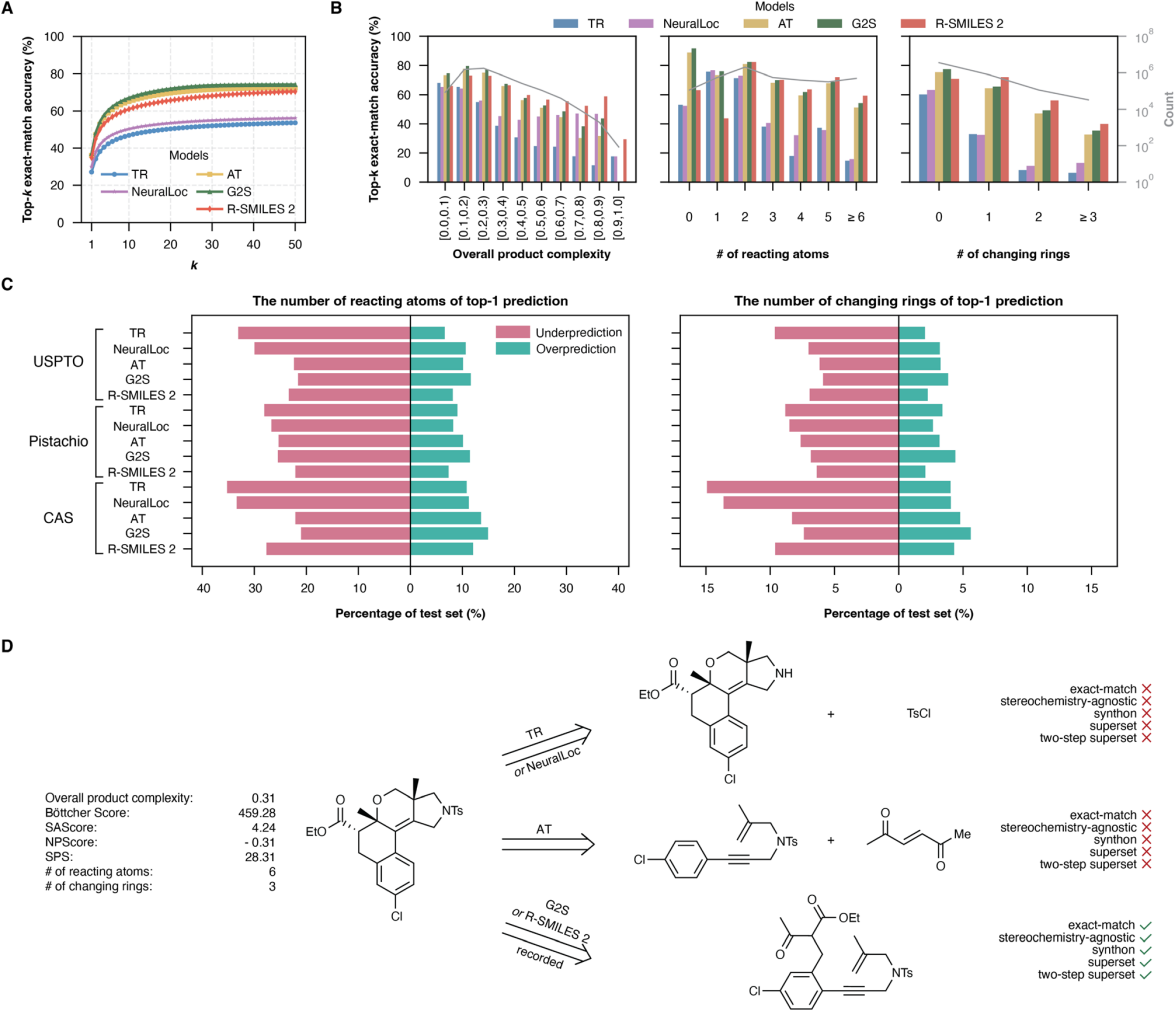
Performance of models trained and tested on CAS: (A) comparison of top-*k* exact-match accuracy. (B) Top-50 exact-match accuracy stratified by product and reaction complexity. (C) Comparison of under- and overprediction of the number of reacting atoms and the number of changing rings in top-1 model predictions across all models and three datasets. The numbers of reacting atoms and changing rings are computed using atom mapping from RXNMapper;^33^ an analogous analysis using a second atom mapping tool^36^ is shown in Fig. S17. (D) Example of a complex reaction correctly predicted only by G2S or R-SMILES 2 but not by TR, NeuralLoc, or AT. Only the top-1 candidates from each are shown, but the ground-truth precursor does not exist within the top-50 predictions for TR, NeuralLoc, and AT.

To evaluate the overall behavior of the one-step models, we first compare their performance across the USPTO, Pistachio, and CAS datasets. Among all models, the template-free models demonstrate higher top-*k* exact-match accuracy than template-based models across all three datasets (Fig. 2A and S9). Stratifying top-*k* exact-match accuracy by product and reaction complexity reveals that the models' performance declines as product and reaction complexity increases (Fig. 2B). This corroborates anecdotal observations of CASP performance.

We then compare the number of reacting atoms and changing rings in the models' predictions to the ground-truth to better understand how the models handle complex reactions. Our analysis focuses on the numbers of reacting atoms and changing rings in the proposals relative to what is recorded, regardless of whether the correct atoms or rings are modified. Interestingly, even though the training and test sets were selected to have minimal differences in distribution (Section S2.4), all models empirically underpredict both the number of reacting atoms and the number of changing rings in their top-1 predictions (Fig. 2C).

For more complex products and complex transformations, AT and G2S still outperform TR (Fig. 2B). A representative example shown in Fig. 2D involves an intramolecular [2 + 2 + 2] cycloaddition between a 1,3-dicarbonyl compound and a 1,6-enyne.^37^ Notably, G2S and R-SMILES 2 recognize the underlying [2 + 2 + 2] cycloaddition motif and recovers the correct precursors despite the closest training example being substantially different (Fig. S30). TR and NeuralLoc, on the other hand, suggest a simpler nitrogen-protection transformation. AT proposes an unreasonable one, which may undergo a [2 + 2 + 2] cycloaddition but cannot form the desired product.

### 3.2 Failure mode 1: models fail to capture stereochemical logic

**Fig. 3:**
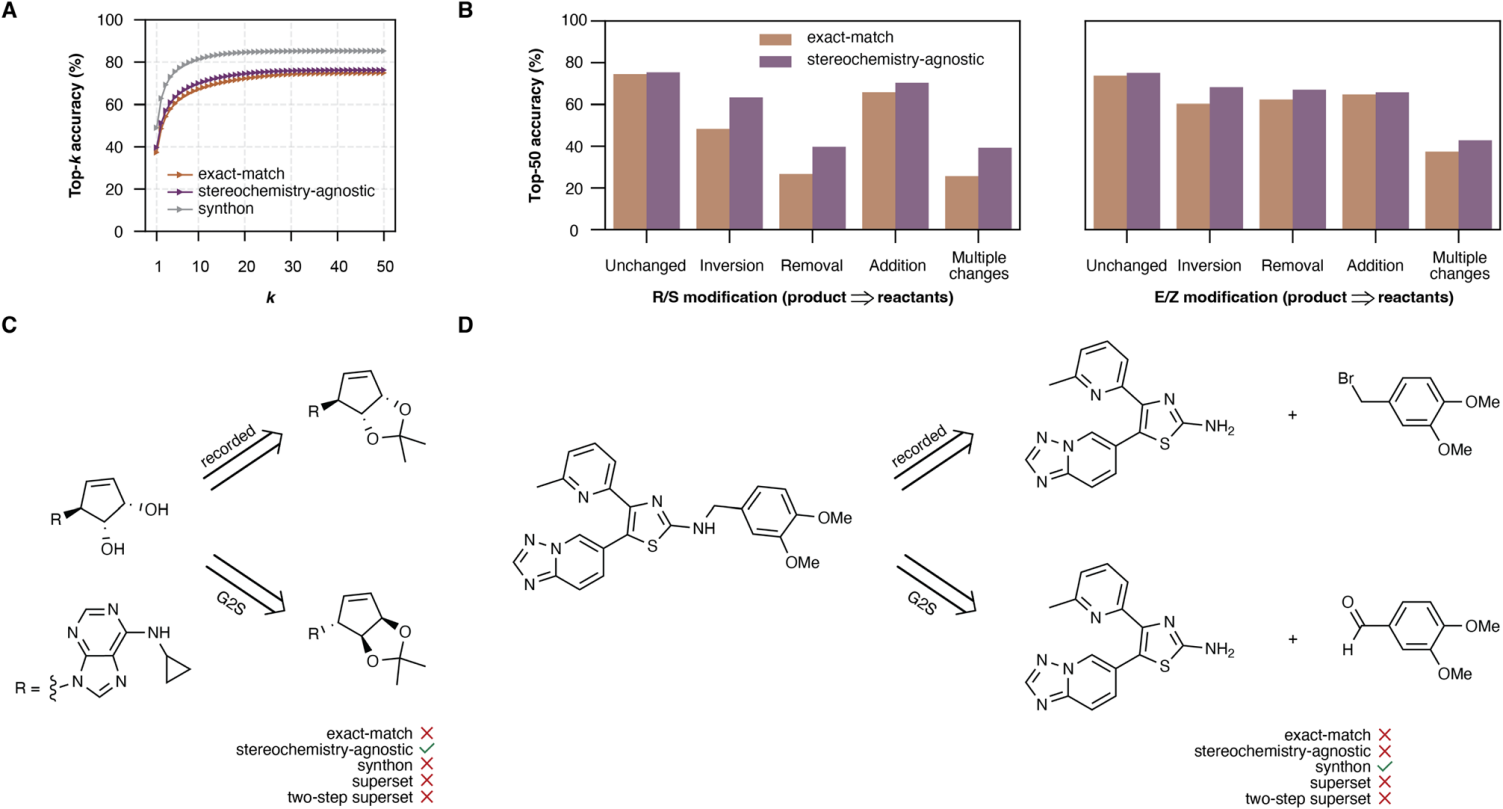
Performance of G2S trained and tested on the CAS dataset: (A) performance evaluated using exact-match, stereochemistry-agnostic, and synthon accuracies. (B) Top-50 exact-match and stereochemistry-agnostic accuracy stratified by whether the reaction involves a change in stereochemistry (*R*/*S* and *E*/*Z*). (C) An example of a top-1 candidate precursor predicted by G2S that is incorrect under exact-match accuracy but correct when evaluated with stereochemistry-agnostic accuracy. (D) An example of a top-1 candidate precursor that is correct when evaluated with synthon accuracy. In both cases, no correct prediction appears within the top-50 under exact-match evaluation.

To understand how stereochemical discrepancies affect models' performance, we evaluated predictions using a stereochemistry-agnostic accuracy metric, which compares the predicted and recorded reactions after removing stereochemical information. This metric is able to capture cases such as Fig. 3C, where G2S predicts the enantiomer of the recorded reactant, although the reaction does not involve any stereochemical inversion. When evaluated with stereochemistry-agnostic accuracy, performance increases by 2–3% at top-10 (Fig. 3A and S9). Although the absolute change is small, further stratifying the results by whether the reaction involves a change in stereochemistry shows greater discrepancies for the subset of reactions where stereochemical changes occur (Fig. 3B, Section S5.4).

We note that some published one-step retrosynthesis models either completely ignore stereochemistry in their molecular representations or simply copy stereochemistry from the product to reactants.^7,38,39^ Such approaches are less helpful for the synthesis of complex molecules where stereochemistry can be essential, for example in stereoselective or asymmetric synthesis.^34^

### 3.3 Failure mode 2: models predict the correct disconnections but assign different leaving groups from the recorded reference

Another common failure mode is the prediction of recorded disconnections but assignment of different leaving groups compared to the literature-reported precursors. For example, G2S predicts the product forming *via* reductive amination, which is chemically feasible, while the literature reports a substitution reaction (Fig. 3D).^40^ Because a synthon can represent the strategic disconnection underlying a reaction, we quantify this failure mode using synthon accuracy, which compares the synthons of the predicted and recorded precursors. Evaluating performance with synthon accuracy, performance increases by 12–15% at top-10 (Fig. 3A and S9).

For the failure cases identified by synthon accuracy, we examined some typical differences between the leaving groups of recorded and predicted precursors (Section S5.5). Common examples include halogen mismatches, where only the halogen differs. This type of failure can be captured by the halogen-agnostic accuracy metric.^14^ Other examples include minor structural variations such as methyl *versus* ethyl groups, or carboxylic acids *versus* their derivatives. The model sometimes predicts the correct leaving groups but assigns them to different reactants, which is commonly observed in cross-coupling reactions. This abstraction of leaving groups is conceptually aligned with, but more lenient than, recent higher-level retrosynthesis formulations that emphasize strategic disconnections in a multi-step search.^41^ Our synthon accuracy effectively distinguishes model failures due to incorrect strategic disconnections from failures arising from differences in leaving groups.

### 3.4 Failure mode 3: models fail in reactions involving multi-stage transformations

**Fig. 4:**
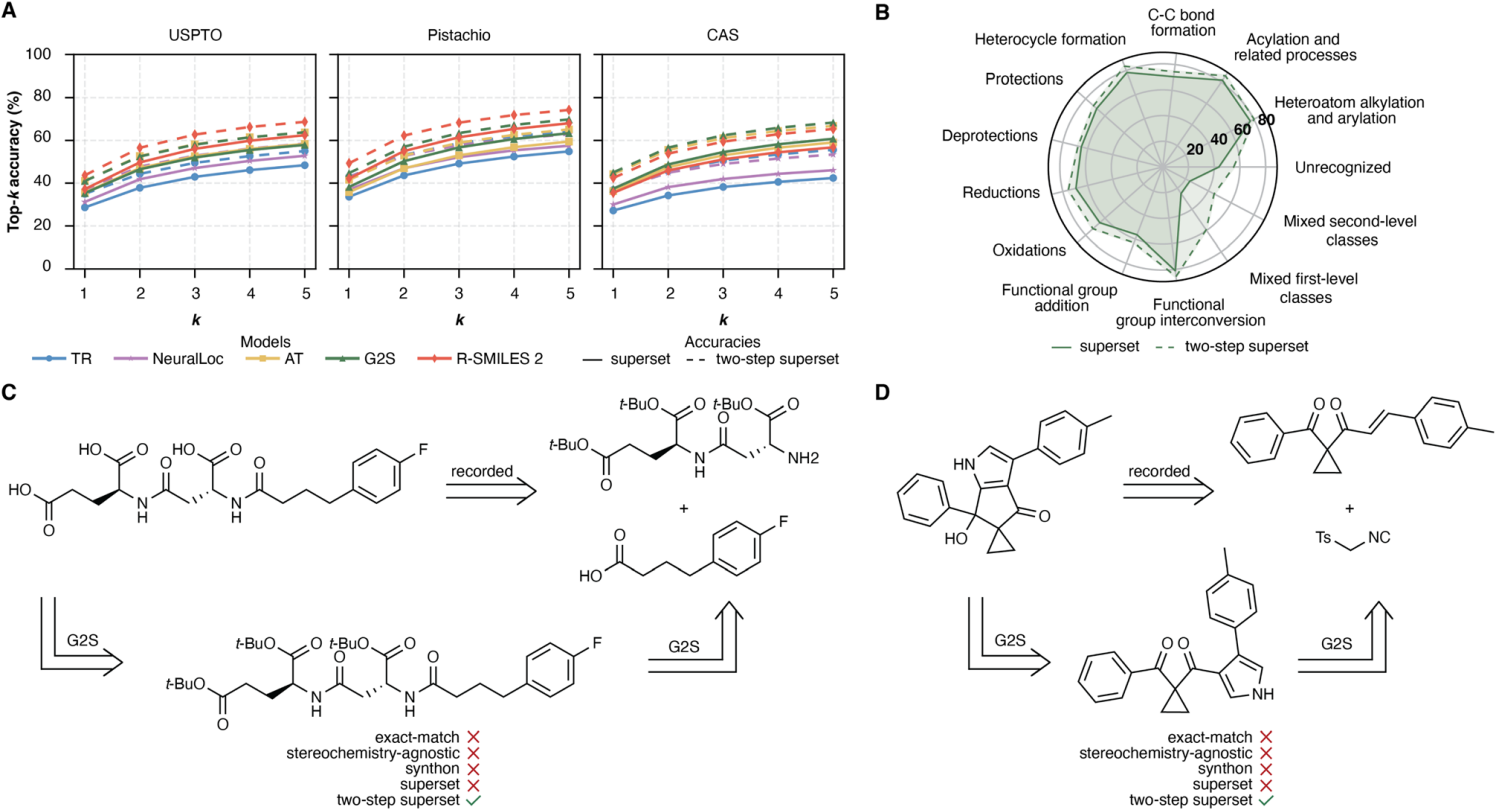
(A) Top-*k* superset *versus* two-step superset accuracies across all models and three datasets, where *k* refers to the number of candidates considered in the first pass. (B) Top-5 superset and two-step superset accuracies of G2S on CAS, stratified by reaction class. (C) and (D) Examples of a precursor incorrectly predicted in the first pass (no correct suggestion within the top-50, only the top-1 candidates are shown) but correctly predicted in the second pass (top-1).^42,43^

In some cases, a model's initial prediction does not match the literature-reported precursor(s), but when it is fed back into the model, the second pass successfully recovers the literature-reported precursor(s). For example, the recorded entry shown in Fig. 4C is a two-stage transformation where G2S predicts only the deprotection in the first pass and requires a second pass to successfully propose the amide coupling. To capture the possibility of recovering the recorded precursors, the largest predicted precursor is fed back into the model, and the results are evaluated using two-step superset accuracy. We evaluate with superset accuracy, instead of two-step exact-match, since correct smaller precursors may already be generated in the first pass (Fig. S7). Under superset evaluation, a prediction is considered correct if the predicted precursors contain all recorded precursors. Evaluating performance with two-step superset accuracy, we observe that the performance increases by 6–17% at top-5 (Fig. 4A).

Notably, this failure mode frequently arises from the presence of multiple transformations within a single recorded entry. Common examples include multi-stage reactions, which involve multiple reaction classes. NameRxn,^44^ which provides a three-level hierarchical reaction classification scheme, often assigned these reactions as 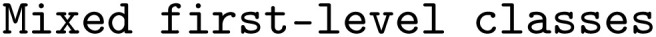, 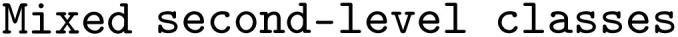, or 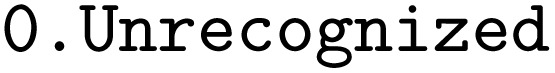 (Section S2.2.2). When stratifying the accuracies by reaction classes, we find that the largest performance gains occur for reactions labeled as mixed-classes or unrecognized, supporting the conclusion that a substantial portion of these entries involve multi-stage transformations (Fig. 4B and S10). Additionally, in another reaction example shown in Fig. 4D, which is not a multi-stage reaction, G2S predicts first-pass precursors containing a correct subset of the bonds to disconnect relative to the recorded set. The first-pass prediction closely resembles the intermediate after [3 + 2] cycloaddition proposed in the literature.^43^ This complementary two-step superset accuracy metric can capture not only multi-stage reactions but also tandem reactions. In the context of a multi-step pathway search, predictions passing this metric would be able to recover the recorded pathway, different only by an additional unrecorded intermediate.

### 3.5 Failure mode 4: models propose the reported precursors but rank them too low

Some failures arise when the reported precursors are generated by the one-step model but receive ranks too low to be selected during a multistep search. Because generating large numbers of precursor candidates during expansion and evaluating them during selection quickly become computationally expensive, data-driven retrosynthesis tools typically prune the number of predictions considered at each step to limit the branching ratio (*e.g.*, retaining only the top-50 predictions when expanding the search tree^17,21,45^). Therefore, when the reported precursors are ranked below the retained set, they cannot be recovered during a multistep search. Nevertheless, pruning remains necessary in practice, as recovering a literature-recorded pathway offers limited practical value if it appears only among an extremely large number of alternatives.

To quantify the contribution of this ranking- and pruning-induced failure mode, we compute the theoretical upper bound on top-*k* exact-match accuracy obtained when all precursors that a model can produce are taken into account, which we refer to as the top-∞ exact match accuracy. We focus on the template-relevance method for the analysis of top-*∞* accuracy: for template-free methods, computing top-*∞* accuracy is not practical or meaningful, as in principle any SMILES string could be generated. In contrast, for template-based models, the total space of precursors that can be generated is defined by the templates, allowing for a meaningful upper bound on the top-*k* accuracy to be computed through exhaustive template enumeration, though at a substantial computational cost. Thus, we evaluate the top-*∞* accuracy for the template-relevance model by exhaustively applying all templates seen by the model during training. We find that 4–9% of the failures in recovering the reported reactions can be attributed to the reported precursors existing among the model's predictions but appearing beyond the top-50 (Table 1 top-*∞*, freq. ≥ 5).

**Table 1:** Template-relevance model's exact match accuracies (%) across datasets. Top-*∞* (freq. ≥ 5) refers to the maximum accuracy obtainable for the model when exhaustively applying all templates used during training (*i.e.*, templates with 5 or more reference reactions). Top-*∞* (all) is the maximum accuracy obtainable for the model when exhaustively applying all templates extracted from the training set regardless of frequency

Dataset	Exact match accuracy (%)		
	Top-50 (freq. ≥ 5)	Top-*∞* (freq. ≥ 5)	Top-*∞* (all)
USPTO	61.45	66.46	76.03
Pistachio	67.24	75.92	81.47
CAS	53.63	64.88	72.33

In our standard setup, template extraction applies a minimum precedent count of five reactions in the training set to ensure sufficient examples for reliable learning. To mitigate the effects of this filtering choice and to provide the most generous possible coverage for the template-based approach, we additionally repeat the top-*∞* analysis using the complete set of templates extracted from the training data prior to frequency filtering. This expanded set includes templates that were not seen during training (*i.e.*, 1 ≤ freq. ≤ 4) and thus represents disconnections that are theoretically available to the method but not directly learned by the model.

With this full template set, the coverage of reactions in the test set increases by an additional 5–10% (Table 1, top-*∞*, all). These results indicate that several reported reactions are not fundamentally inaccessible to the template-based model, but are missed because the relevant transformations are either not ranked highly enough or are pruned from the template set during training. However, including low-frequency templates introduces many highly specific and less broadly applicable transformations (Section S5.6.1), and in both cases, whether using the default or complete template set, exhaustively enumerating all templates imposes a significant computational burden during template application and precursor selection because of the large template set and the resulting precursor space (Section S5.6.2).

### 3.6 Summary of failure modes

Across all datasets, approximately 93–95% of reactions were either (i) successfully recovered with exact-match accuracy or (ii) assigned to at least one specific failure mode captured by the complementary accuracy metrics (Table 2). The remaining 5–7% of instances that do not fall into any specific category often arise from more complex transformations and complex products (Fig. S28, S29, S32–S35). Additionally, some failures can be attributed to errors in the underlying reaction data (Fig. S5), which unfortunately prevent the models from accurately reproducing the reported precursors.

**Table 2:** Summary of failure cases across datasets. Accuracies reflect the percentage of test reactions where predictions from at least one model satisfy at least one of the metrics

Models	Accuracy metric	Top-*k* predictions	Accuracy (%)		
			USPTO	Pistachio	CAS
TR, NeuralLoc, AT, G2S, R-SMILES 2	Exact-match	50	82.53	86.06	85.17
TR, NeuralLoc, AT, G2S, R-SMILES 2	Stereochemistry-agnostic	50	83.21	87.27	86.86
TR, NeuralLoc, AT, G2S, R-SMILES 2	Synthon	50	91.30	92.00	92.67
TR, NeuralLoc, AT, G2S, R-SMILES 2	Superset	50	82.66	86.21	85.56
TR, NeuralLoc, AT, G2S, R-SMILES 2	Two-step superset	5	78.30	82.48	83.00
**All 5 metrics and models**		—	**92.34**	**93.06**	**94.44**
TR	Exact-match	*∞* (freq. ≥ 5)	66.46	75.92	64.88
TR	Exact-match	*∞* (all)	76.03	81.47	72.33
**All metrics and models**		—	**92.65**	**93.94**	**95.05**

## 4 Discussion

### 4.1 Complementary evaluation methods provide a more nuanced understanding of one-step model performance

Top-*k* exact-match accuracy is the standard metric for assessing one-step retrosynthesis performance, since it directly measures whether a model reproduces the recorded precursors. However, this metric alone does not provide insight into how these models fail to recover them. Without additional evaluation criteria, distinct types of errors are grouped together, making it difficult to interpret how models behave and which aspects of their performance require improvement.

In contrast to round-trip accuracy that relies on reaction prediction models, these complementary accuracy metrics allow quantification of the failure modes while still evaluating predictions against the recorded precursors, which serve as the only proof of experimental feasibility. Stereochemistry-agnostic accuracy identifies cases where the predicted transformation is structurally correct aside from stereochemical details. Synthon accuracy highlights when the correct bond-breaking/forming pattern is correctly predicted even if the specific leaving groups differ. Superset and two-step superset accuracy measure whether the model proposes all required reactants, either in a single step or through plausible multi-stage transformations. Top-∞ exact-match accuracy further quantifies failures attributable to ranking and distinguishes cases that are fundamentally inaccessible to the model from those that could be generated with more compute. Moreover, it demonstrates that the decline in top-*k* exact-match accuracy with increasing molecular complexity is not simply a consequence of having more potential sites of transformation, which would make it harder for the recorded precursors to appear among the top-ranked predictions (Fig. S27). We recommend that future studies of one-step retrosynthesis models adopt these broader sets of evaluation criteria beyond the standard top-*k* exact match accuracy to more accurately assess model capabilities and limitations.

### 4.2 Datasets that better represent complex chemical space are crucial for improving model performance

Our analyses show that model performance declines for reactions with complex products, multi-center bond changes, or stereochemically relevant transformations (Sections 3.1 and S5.4). This space of reactions, which are relevant to many challenging synthetic targets of interest, remains underrepresented in existing datasets (Tables S3 and S10). Because one-step models learn directly from the reactions in their training data, limited representation of both complex transformations and chemistries associated with complex products restricts their ability to generalize to the reactions required for challenging synthetic targets. Improving the coverage of this chemical space may therefore require more deliberate data strategies. Enhanced curation to better capture multi-center reactivity, rearrangements, and stereochemical outcomes, along with targeted data collection^46^ or data augmentation strategies^47–49^ focusing on this underrepresented space, could help mitigate the performance gap.

### 4.3 Enhancing model capacity to capture complex transformations

However, these performance declines cannot be attributed solely to dataset coverage or simple class imbalance. Even when training and test sets are curated to have closely matched distributions across reaction complexity metrics, one-step models underpredict the complexity of the recorded transformations. Additionally, training on a complexity-rebalanced dataset does not remove the systematic bias toward simpler transformations (Section S5.3). This systematic bias toward predicting simpler transformations indicates that the models are not fully learning or reproducing the complexity present in the training data itself. These results suggest that reweighting the training distribution alone is insufficient to resolve the observed bias. Improving performance on complex transformations will therefore require a multifaceted approach, potentially involving model architectures and more sophisticated training objectives in parallel with efforts to expand datasets.

### 4.4 Implications for the practical use of CASP tools

While our analysis focuses on the failure modes of one-step retrosynthesis models in recovering literature-reported precursors, the results have direct implications for the use of CASP tools in prospective applications, where the goal is not to reproduce a known route but to design new routes to new target molecules. In this setting, CASP tools can serve as sources of retrosynthetic ideas for further examination and refinement. The gap between exact-match and synthon accuracy is instructive in this regard: a model may correctly identify the underlying strategic disconnection even when the specific leaving group choices differ from those in reported reactions. In practical synthesis planning, users may therefore benefit from considering alternative functional group or leaving group choices compatible with the disconnections identified by the model, even when such specific implementations are not explicitly proposed.

Our analyses also inform how one might most effectively use these models, even when initial proposals may not appear promising. For complex molecules, it may be more productive to apply CASP tools to intermediates from which the final target can be synthesized using known chemistry rather than to the final target directly. The observed decrease in performance with increasing product complexity, despite the underlying transformations often being relatively simple, provides quantitative support for this intermediate-focused strategy. Similarly, since correct disconnections may be present among the model's outputs but ranked too low to appear among the top suggestions, examining lower-ranked candidates may provide additional inspiration. Additionally, for stereochemically rich targets, querying the model both with and without explicit stereochemical information may also be valuable, as this can help separate scaffold-level disconnection strategies from questions of stereochemical control.

While model suggestions alone can be informative, comparison with related known reactions can provide additional value for assessing reaction feasibility. For template-based methods, the reaction precedents underlying each template can be helpful to assess a proposed disconnection; for template-free methods, similarity searches over reaction databases can serve an analogous purpose. Although template-free models may better recover literature-reported reactants, their predictions do not inherently provide the same direct links to templates and their source reactions, making external comparison to related known reactions particularly useful for interpretation and evaluation. As with any computational tool, extracting the most value from these suggestions requires an informed understanding of where and how they fail, as characterized here.

## 5 Conclusions

In this work, we provide a quantitative analysis of how one-step retrosynthesis models fail to recover the literature-reported precursors across three reaction datasets. We quantitatively show that model performance declines with increasing complexity of target molecules or transformations. By comparing the distributions of the number of reacting atoms and changing rings in the recorded reaction and model's prediction, we found that the models exhibit a persistent bias toward simpler transformations, underpredicting the extent of structural changes observed in the literature even when equally-complex examples are included in the training data. This trend highlights that the high-complexity cases, often those of greatest synthetic interest, are where current models face the greatest challenges in reproducing literature-reported precursors. In addition, we quantified the specific failure modes of one-step models using complementary accuracy metrics, including stereochemistry-agnostic accuracy, synthon accuracy, superset accuracy, and two-step superset accuracy, and recommend their use in future evaluations, as they provide a more informative picture of model behavior than exact-match accuracy alone. Finally, we discuss how an understanding of these failure modes can inform the use of CASP tools in practice, where models are meant to provide sources of retrosynthetic ideas for further examination rather than exact recovery of literature.

We acknowledge that retrosynthesis is inherently a one-to-many problem, where multiple possible precursor sets are feasible regardless of whether they are recorded in the dataset. However, systematically evaluating such alternatives remains challenging. Literature-reported precursors represent disconnections that have been successfully executed in practice and thus provide the only experimentally validated evidence. Therefore, establishing the reliability of one-step retrosynthesis models with respect to literature-reported reactions remains a necessary foundation of quantitative evaluation.

## Author contributions

S. T., J. R., and C. W. C. conceptualized the project. S. T. and J. R. conducted computational experiments, analyzed the results, and prepared the original draft. All authors reviewed and approved the final manuscript.

## Conflicts of interest

There are no conflicts to declare.

## Supplementary Material

SC-017-D6SC01323F-s001

## Data Availability

The reaction data used in this article are: USPTO-Full^25^ was obtained from Coley *et al.*^50^ Pistachio (2025Q2): Pistachio is a commercial resource that can be licensed from NextMove Software at https://www.nextmovesoftware.com/pistachio. CAS: These reactions originate from patents and publications dated 2010–2015 and were curated by CAS, a division of the American Chemical Society. More details on the features of this reaction data set can be found at https://www.cas.org/cas-data/cas-reactions. The code for all models is available at https://gitlab.com/mlpds_mit/askcosv2/retro distributed under the MIT License. The split data, model checkpoints, and the code for the analysis are available at https://github.com/suongbatran/one_step_retro_failure_mode distributed under the MIT License. Supplementary information (SI): Additional results and data processing details. See DOI: https://doi.org/10.1039/d6sc01323f.
