# Supplementary material for: Quantifying the failure modes of current one-step retrosynthesis models
Source: Chem Sci. 2026 Jun 4;17(28):14054–63. doi: 10.1039/d6sc01323f (PMC13262262; doi:10.1039/d6sc01323f)
Supplement: SC-017-D6SC01323F-s001 [file SC-017-D6SC01323F-s001.pdf]

Supplementary Information (SI) for Chemical Science.
This journal is © The Royal Society of Chemistry 2026

Supplementary Information for
Quantifying the Failure Modes of Current One-step Retrosynthesis Models

Suong B. A. Tran,[a‡] Jihye Roh,[b‡], and Connor W. Coley[b,c*]

[a] *Department of Chemistry, Massachusetts Institute of Technology, 77 Massachusetts Avenue, Cambridge, MA 02139, USA.*
[b] *Department of Chemical Engineering, Massachusetts Institute of Technology, 77 Massachusetts Avenue, Cambridge, MA 02139, USA.*
[c] *Department of Electrical Engineering and Computer Science, Massachusetts Institute of Technology, 77 Massachusetts Avenue, Cambridge, MA 02139, USA.*
‡ These authors contributed equally to this work.
[*]*E-mail: ccoley@mit.edu*

Contents

S1 Complexity Metrics **2**
S1.1 Product complexity . . . 2
S1.2 Reaction complexity . . . 3

S2 Details on Data **4**
S2.1 Data preprocessing . . . 4
S2.2 Data distribution . . . 5
S2.2.1 Product and reaction complexity . . . 5
S2.2.2 Reaction class . . . 6
S2.3 Data bookkeeping . . . 7
S2.3.1 Inconsistency across datasets . . . 7
S2.3.2 Inconsistency within a dataset . . . 9
S2.4 Splitting data . . . 11

S3 Models **13**
S3.1 Model hyperparameters, training, and additional details . . . 13
S3.2 Discussion on model choice for analysis . . . 14

S4 Evaluation metrics **15**

S5 Additional Results **16**
S5.1 Random split Results . . . 16
S5.2 Analysis for USPTO and Pistachio . . . 17
S5.3 Evaluation with complexity-rebalanced dataset . . . 19
S5.4 Additional analysis of model underprediction of number of reacting atoms and changing ring . . . 20
S5.4.1 Left-shift in distributions of number of reacting atoms and changing rings . . . 20
S5.4.2 Mapping affects the analysis of the count of reacting atoms and changing rings . . . 24
S5.5 Stratified results by product stereochemistry and stereochemical change during reaction . . . 27
S5.6 The difference between predicted and recorded leaving groups . . . 31
S5.7 Template enumeration results . . . 33
S5.7.1 Examples of filtered out templates extending coverage . . . 33
S5.7.2 Unique precursors generated per product molecule . . . 40
S5.7.3 Template-based model performance stratified by complexity . . . 41
S5.8 Summary of complementary metrics for all models and three datasets . . . 42
S5.9 Additional reaction examples . . . 46

S1 Complexity Metrics

S1.1 Product complexity

The code for computing product complexity can be accessed and deployed from ASKCOS.[1]

Table S1 Summary of product metrics.

Type	Metrics	Description
Product complexity metric	Böttcher score[2]	Molecule is quantified in âĂIJcomplexity bitsâĂİ by accounting for atomic features such as valency, stereochemistry, and local diversity.
	Spacial Score (SPS)[3]	Molecule is evaluated on each atom's hybridization (penalizing unsaturation), stereoisomerism, presence of non-aromatic rings, and number of heavy-atom neighbors (prioritizing branching).
	SAScore[4]	The metric is defined as fragmentScore minus complexityPenalty; fragmentScore is obtained by averaging the contributions of molecular fragments according to their frequency in the PubChem database, while complexityPenalty captures structural features such as rings, stereocenters, and macrocycles.
	NPScore[5]	The similarity of molecules to the structural space of natural products is quantified by a Bayesian measure.
	Overall product complexity	Calculated as the average of the four metrics above, each linearly normalized to a scale of 1 using specific minimum and maximum values to each dataset.

Table S2 Minimum and maximum values of product complexity metrics in the document-based test set for each dataset.

Metric	Dataset	Min	Max
Böttcher Score	CAS	7.50	3515.79
	Pistachio	7.50	12894.03
	USPTO	9.00	4265.80
SPS	CAS	2.80	105.67
	Pistachio	3.20	108.00
	USPTO	3.60	108.00
SAScore	CAS	1.00	9.66
	Pistachio	1.00	10.00
	USPTO	1.00	10.00
NPScore	CAS	-4.06	4.11
	Pistachio	-4.08	4.04
	USPTO	-3.77	4.00

S1.2 Reaction complexity

- **Number of reacting atoms:** This metric quantifies the atoms in the product that undergo a chemical change during the reaction. Reacting atoms in the product are identified by comparing each mapped atom between the product and reactant structures. Each mapped atom in the product is considered reacting if it satisfies any of the following criteria:
  - its formal charge changes
  - the types of neighboring atoms bonded to it differ
  - the set of specific neighboring atoms it is bonded to (based on atom mapping) change
  - the bond orders to neighboring atoms differ
- **Number of changing rings:** This metric counts the number of ring systems in both reactants and product that are different in the reaction. Fully-mapped rings are extracted from both reactants and product, and the total number of rings affected by the reaction is determined as the symmetric difference between the sets of these rings in the reactants and products. In deprotection reactions, for example, by-products containing protecting groups are often excluded, causing atoms of protecting-group unmapped. Therefore, only rings containing all mapped atoms are considered (Fig. S1C). (Fig. S1C).

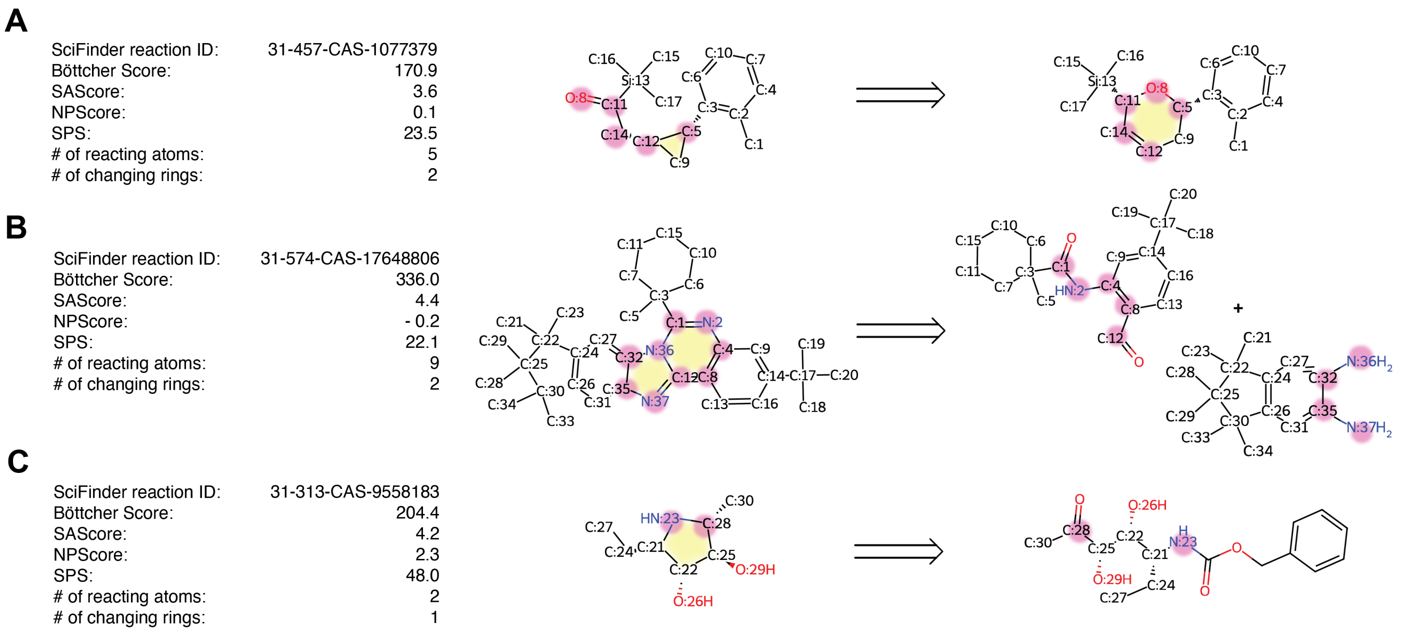

Figure S1 Example of reactions involving A) ring rearrangement and B) ring formation. C) A deprotecting reaction where the protecting group is not recorded as a product, so only the fully mapped ring structure is considered.

S2 Details on Data

We conduct the analysis for three datasets: USPTO-Full[6], Pistachio[7] (2025Q2 version), and a subset of CAS containing single-step reactions from 2010 to 2015.[8] For the USPTO dataset, we use the version of USPTO-Full dataset cleaned by Coley *et al.*[9]. Each dataset contains chemical reactions represented in the Simplified Molecular Input Line Entry System (SMILES) format.[10] In this format, reactants, agents, and products are concatenated using > to form the reaction SMILES (i.e., `REACTANTS>AGENTS>PRODUCTS`), and multiple molecules within each field are separated by a dot. Reaction SMILES also include atom-mapping numbers that specify the correspondence between atoms in the reactants and products.

S2.1 Data preprocessing

The datasets were obtained from their original sources. Except for CAS, we only kept reactions where all reactants were added in one experimental stage. This was only possible for CAS because we have access to detailed experimental records showing when each reactant, agent, or solvent was added. All datasets underwent the following general preprocessing steps:

- **Step 1:** Deduplicate reactions based on mapped SMILES (from original dataset), keeping the first instance.
- **Step 2:** Classify reactions using NextMove's NameRxn[11] tool, which provides a three-tier hierarchical reaction classification. Reactions that fail classification due to incorrect or incomplete SMILES are then removed.
- **Step 3:** Clean reaction SMILES:
  - Agents are moved to reactants; chemicals present in both reactants and products are moved to agents.
  - Remove products with fewer than 5 heavy atoms.
  - Remove reactions that meet any of the following criteria:
    - Agents or products contain two atoms with the same mapping number.
    - Mapped atoms in reactants and products correspond to different elements.
  - Remove atom mappings for atoms not present in both reactants and products, then move fully unmapped reactants and products to the agent side.
- **Step 4:** Retain only reactions with a single product.
- **Step 5:** Remove atom mappings and deduplicate reactions based on unmapped SMILES without agents (i.e., `reactants>>product`).

S2.2 Data distribution

S2.2.1 Product and reaction complexity

In general, the distributions of the reaction complexity metrics are comparable between datasets. CAS generally exhibits slightly higher mean values than USPTO and Pistachio (Table S3). CAS products show higher average Böttcher score and Spacial Score (SPS), suggesting a greater degree of product structural complexity. NPScore values are more negative for USPTO, reflecting a lower proportion of natural product–like compounds relative to CAS and Pistachio. The numbers of reacting atoms and changing rings are similar across all datasets, with most reactions involving two reacting atoms and no ring changes. Since the CAS dataset only includes reactions from 2010 to 2015, we also focus on reactions from this period for comparison. When comparing the 2010–2015 subsets, three datasets display consistent trends with their full counterparts, though USPTO shows a slight increase in Böttcher Score, indicating marginally more complex product in that period.

Table S3 Summary statistics of complexity metrics across datasets. Numbers of reacting atoms and changing rings are computed based on the original atom mapping provided by the dataset.

Metric	Dataset	Mean	Median	10%	25%	75%	90%
Böttcher Score	USPTO	289 ± 127	275	139	192	370	453
	USPTO (2010–2015)	316 ± 127	312	155	217	401	477
	Pistachio	330 ± 178	307	151	212	414	520
	Pistachio (2010–2015)	318 ± 162	299	148	205	401	496
	CAS	311 ± 183	280	136	194	387	499
SPS	USPTO	15.3 ± 5.86	13.8	10.3	11.1	17.8	22.3
	USPTO (2010–2015)	15.4 ± 5.45	14.2	10.4	11.3	18.0	22.1
	Pistachio	16.4 ± 6.60	14.9	10.5	11.5	19.3	24.3
	Pistachio (2010–2015)	16.8 ± 7.36	14.7	10.4	11.4	19.7	25.8
	CAS	16.7 ± 7.70	14.2	10.5	11.3	19.5	26.4
SAScore	USPTO	3.02 ± 0.74	2.97	2.13	2.49	3.47	3.96
	USPTO (2010–2015)	3.10 ± 0.71	3.07	2.22	2.59	3.54	4.00
	Pistachio	3.30 ± 0.90	3.21	2.25	2.67	3.79	4.43
	Pistachio (2010–2015)	3.27 ± 0.88	3.19	2.25	2.65	3.75	4.38
	CAS	3.27 ± 1.04	3.13	2.11	2.53	3.80	4.58
NPScore	USPTO	-0.78 ± 0.76	-0.85	-1.65	-1.29	-0.35	0.17
	USPTO (2010–2015)	-0.95 ± 0.70	-1.02	-1.77	-1.43	-0.55	-0.06
	Pistachio	-0.72 ± 0.77	-0.81	-1.60	-1.24	-0.31	0.22
	Pistachio (2010–2015)	-0.67 ± 0.82	-0.78	-1.60	-1.23	-0.23	0.36
	CAS	-0.47 ± 0.90	-0.52	-1.53	-1.09	0.03	0.66
# of reacting atoms	USPTO	2.82 ± 1.83	2	1	2	3	5
	USPTO (2010–2015)	2.73 ± 1.68	2	1	2	3	5
	Pistachio	2.60 ± 2.26	2	1	2	2	5
	Pistachio (2010–2015)	2.36 ± 1.94	2	1	1	2	4
	CAS	2.99 ± 2.34	2	1	2	4	6
# of changing rings	USPTO	0.15 ± 0.43	0	0	0	0	1
	USPTO (2010–2015)	0.15 ± 0.42	0	0	0	0	1
	Pistachio	0.15 ± 0.47	0	0	0	0	1
	Pistachio (2010–2015)	0.13 ± 0.42	0	0	0	0	1
	CAS	0.26 ± 0.61	0	0	0	0	1

S2.2.2 Reaction class

In this study, we classify reactions using NameRXN, which assigns each reaction to one of 10 first-level reaction classes or to `0.Unrecognized` if the reaction is not recognized. `Heteroatom alkylation and arylation` is the most prevalent identified class for the USPTO (22%) and Pistachio (20%) datasets. Pistachio displays a comparatively higher percentage of deprotection reactions (14%) compared to other datasets. CAS contains fewer medicinal–chemistry–relevant reactions, such as `Heteroatom alkylation and arylation`, `Acylation and related processes`, and `Functional group interconversion`, but includes a substantially higher proportion of reactions unclassified as `0.Unrecognized` (Fig. S2). `0.Unrecognized` often contains reactions involving complex transformations (Fig. 4D) or multi-stage transformation (**??**) that are not recognized by NameRxn.

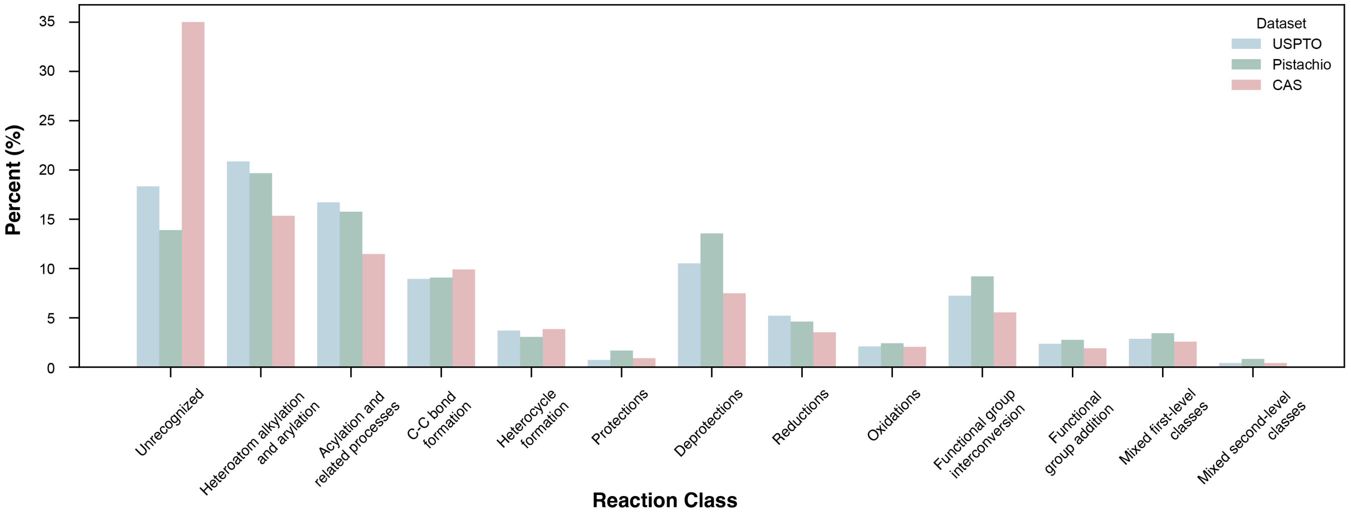

Figure S2 Reaction class distribution across datasets.

`Mixed first-level classes` denotes reactions that involve a deprotection together with an additional reaction class. `Mixed second-level classes` denotes reactions that contain multiple deprotection of different protecting groups (Table S4).

Table S4 Composition of `Mixed first-level` and `Mixed second-level` reaction classes by first-level classes across datasets. Percentages indicate the fraction of reactions within each mixed class that belong to a given first-level class.

Class	USPTO		Pistachio		CAS	
	Mixed first-level classes	Mixed second-level classes	Mixed first-level classes	Mixed second-level classes	Mixed first-level classes	Mixed second-level classes
Heteroatom alkylation and arylation	34.97	0.00	30.69	0.00	30.12	0.00
Acylation and related processes	24.77	0.00	23.14	0.00	25.63	0.00
C-C bond formation	14.09	0.00	13.72	0.00	19.84	0.00
Heterocycle formation	4.81	0.00	4.18	0.00	5.01	0.00
Protections	0.55	0.00	1.96	0.00	0.76	0.00
Deprotections	99.47	100.00	99.45	100.00	100.00	100.00
Reductions	5.97	0.00	6.97	0.00	5.47	0.00
Oxidations	1.01	0.00	1.62	0.00	1.05	0.00
Functional group interconversion	12.20	0.00	14.60	0.00	10.85	0.00
Functional group addition	1.61	0.00	2.61	0.00	1.20	0.00

S2.3 Data bookkeeping

S2.3.1 Inconsistency across datasets

The same type of reaction can be reported differently across datasets. For example, consider the transformation of an aromatic amine into an aromatic azide using sodium aizde and sodium nitrate (Fig. S3). Each dataset encodes this reaction differently: USPTO retains the azide ion, Pistachio includes sodium azide, and CAS does not include any of the agents. As a result, the atoms preserved after preprocessing vary depending on the source. A similar problem occurs in oxidations performed with *tert*-butyl hydroperoxide (Fig. S4). USPTO includes the agent, whereas both Pistachio and CAS do not. These inconsistencies ultimately lead to different set of reactants retrained after preprocessing. This issue becomes particularly important in cases where agents contribute atoms to the final product, which directly impacts computed reaction complexity metrics (i.e., number of reacting atoms and the number of ring changes). Therefore, our analysis focuses on internal dataset evaluation only.

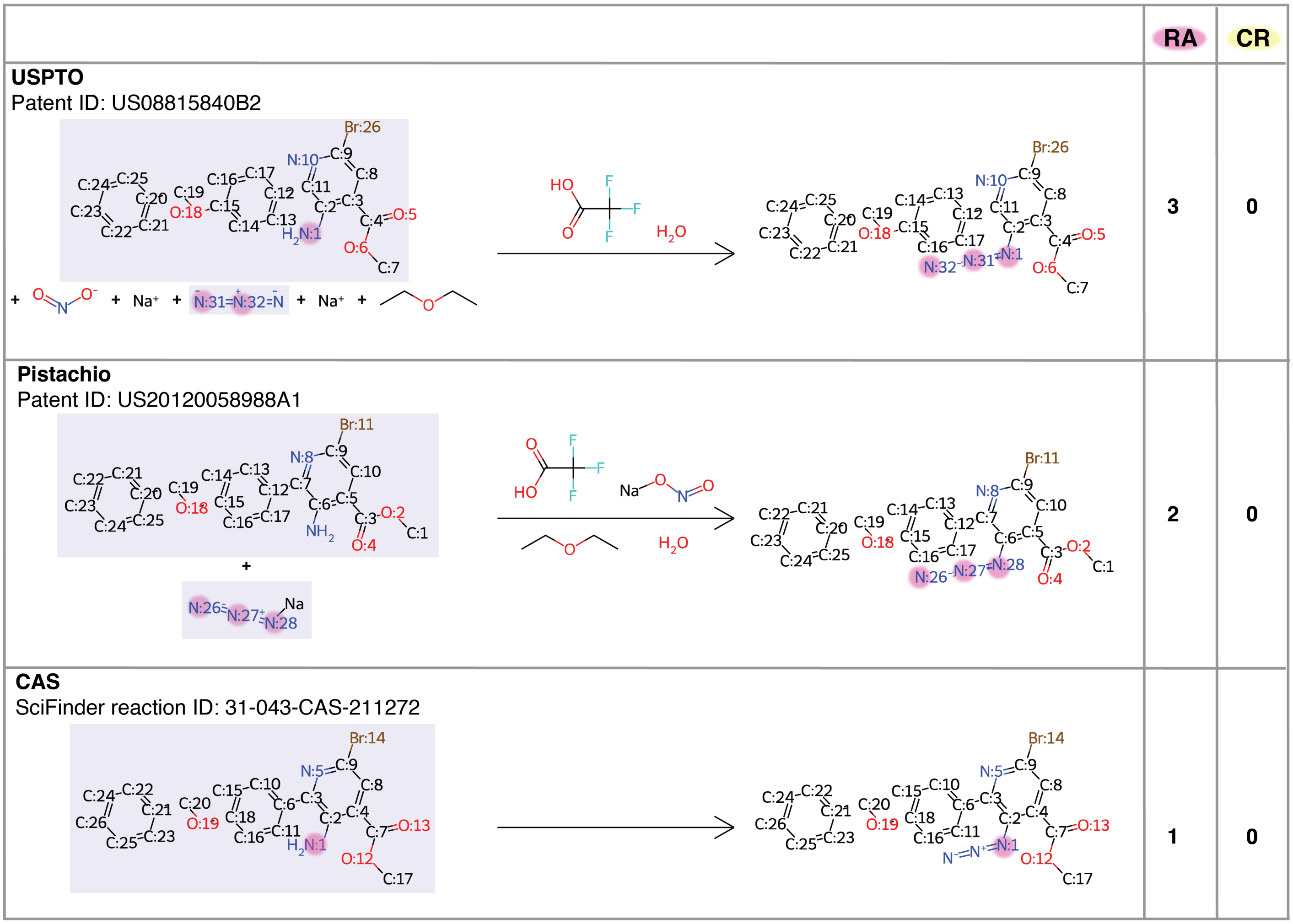

Figure S3 Example of `9.7.2 Amino to azido` reaction recorded differently across datasets. The entries shown here correspond to the raw records of the same reaction with the same substrate. After preprocessing, only the molecules highlighted in the box are retained in the reactant side. RA: reacting atoms, CR: changing rings.

	RA	CR
**USPTO** Patent ID: US06020512 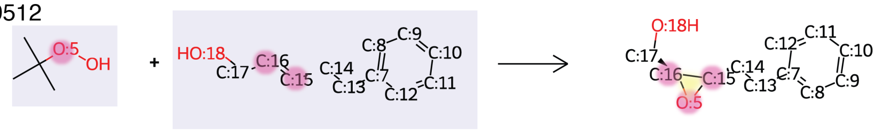	3	1
**Pistachio** Patent ID: US20020128185A1 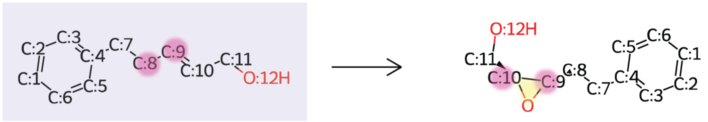	2	0
**CAS** SciFinder reaction ID: 31-279-CAS-9922071 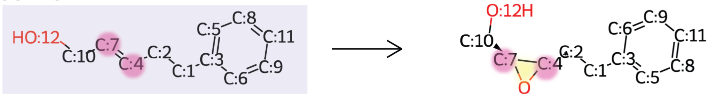	2	0

Figure S4 Example of `4.2.8 Prilezhaev epoxidation` reaction recorded differently across datasets. The entries shown here correspond to the raw records of the same reaction with the same substrate. After preprocessing, only the molecules highlighted in the box are retained in the reactant side. RA: reacting atoms, CR: changing rings.

S2.3.2 Inconsistency within a dataset

Reactions in a dataset are sometimes recorded without all agents required to satisfy stoichiometric needs (Fig. S6A). To account for this inconsistency within a dataset, we introduce superset accuracy, which considers a prediction correct if the predicted precursors form a superset of the recorded reactants. MaxFrag,[12] which evaluates only the largest reactant shared between the ground truth and the prediction, has previously been used; however, this metric is overly relaxed and may incorrectly classify predictions as correct even when they do not have a chemically similar disconnection strategy (Fig. S6B). Our superset accuracy ensures that all documented components are captured, while synthon accuracy offers a more permissive and informative evaluation that focuses on the core disconnection (Fig. S6C). Additionally, within the same dataset, similar reactions may be reported inconsistently, and errors in atom mapping can result in reactions that are chemically equivalent appearing different after preprocessing (Fig. S5).

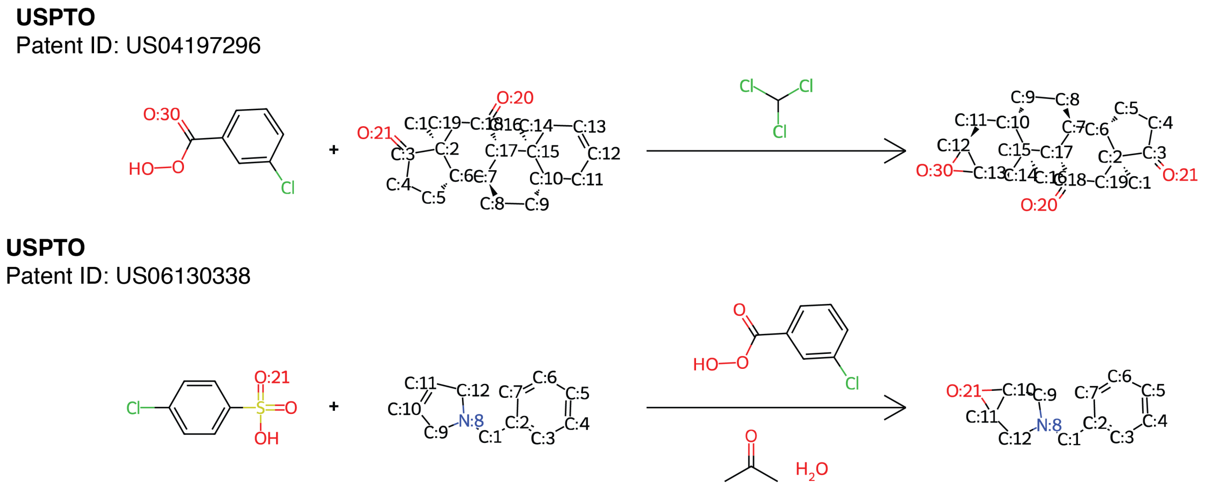

Figure S5 An example of the `4.2.8 Prilezhaev epoxidation` reaction is recorded inconsistently in the USPTO dataset. Due to incorrect atom mapping, the second instance incorrectly includes *p*-chlorobenzenesulfonate as a reactant. This inconsistency makes prediction substantially more challenging.

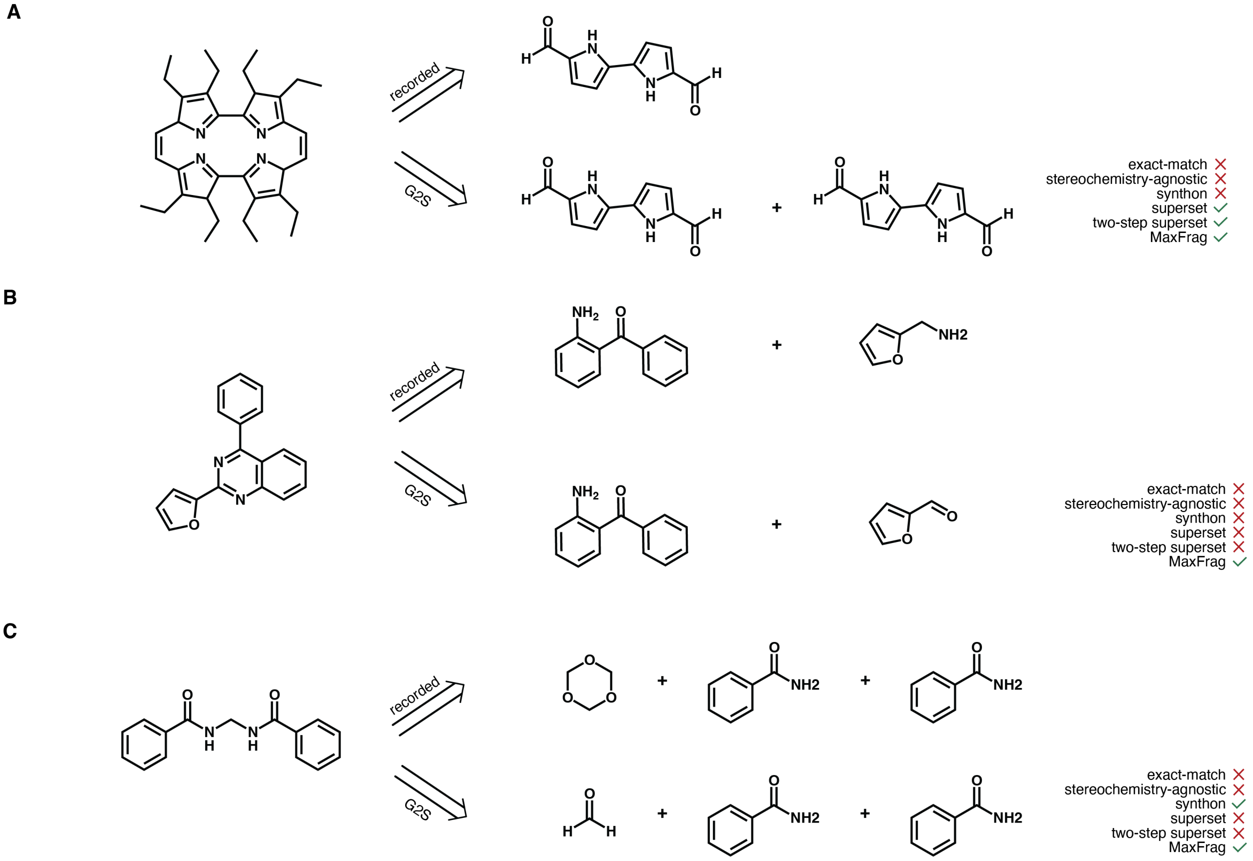

Figure S6 A) Example where reaction is recorded without all agents required to satisfy stoichiometric needs.[13] Example where MaxFrag considers the prediction correct while the superset metric does not: B) The prediction proposes a different transformation compared with the reported reaction.[14] The smaller fragment in the prediction would need to undergo conversion of the aldehyde into the corresponding primary amine, as in the reported reaction, in order to form the product. C) The prediction captures the correct underlying chemistry. The only difference is that 1,3,5-trioxane is used as the formaldehyde source in the literature, whereas the prediction directly proposes formaldehyde.[15]

S2.4 Splitting data

We focus on internal dataset evaluation due to the differences in bookkeeping across datasets. Analysis are mainly focused on document-based split. The ratio of the number of reactions used for the training, validation, and test sets was 75:5:20 (Table S5). For the document-based split, we perform the split randomly at the document level, ensuring that all reactions originating from the same document are grouped into the same set. We only report the overall top-k exact-match accuracy for the random split (75:5:20).

For the document-based split, to ensure a close match between the train and test sets for our analysis, we performed the split using 100 different random seeds and selected the split with the smallest train–test differences in the distribution of reaction complexity metrics, as determined by the two-sample Kolmogorov–Smirnov (KS) test[16] implemented via the `ks_2samp` function in the SciPy library.[17] Although originally defined for continuous distributions, we use the KS statistic purely as a distance metric to compare the empirical distributions of our discrete reaction complexity features. For each candidate split, we computed the KS statistic between the train and test distributions for each reaction complexity metric.

We used the largest KS distance value across the complexity metrics to characterize the worst-case distributional mismatch for that split. We then selected the split with the smallest such worst-case value, which maximizes the worst-case similarity and provides the most balanced match between the train and test distributions (Table S6). While generating these splits, we also included the number of reacting atoms (from forward perspective) when computing the minimum. A mapped atom in the reactant is considered reacting atom if it meets any of the following criteria: its formal charge changes; the types of neighboring atoms, whether mapped or unmapped, change; the set of specific neighboring atoms it is bonded to (based on atom mapping) changes; or the bond orders to neighboring atoms change. This forward definition was used only for generating the splits and not in subsequent analyses.

Table S5 Number of reactions in each dataset before and after preprocessing, and in the train/validation/test splits. The counts before preprocessing include duplicates, where identical reactant–product pairs may appear multiple times.

Dataset	Before preprocessing	After preprocessing	Document-based split			Random split		
			Train	Validation	Test	Train	Validation	Test
USPTO	1,808,254	966,744	729,328	48,477	188,939	725,058	48,337	193,349
Pistachio	21,632,854	4,326,750	3,231,989	210,021	884,740	3,245,062	216,337	865,351
CAS	6,599,048	4,451,647	3,333,498	225,402	892,747	3,338,735	222,582	890,330

Table S6 Two sample Kolmogorov–Smirnov statistics comparing the selected test and train splits across datasets for the document-based split.

Dataset	Metric	KS statistic ($\times 10^{-3}$)
USPTO	# of reacting atoms (forward)	1.69
	# of reacting atoms	1.83
	# of changing rings	0.47
Pistachio	# of reacting atoms (forward)	0.74
	# of reacting atoms	1.00
	# of changing rings	0.58
CAS	# of reacting atoms (forward)	0.96
	# of reacting atoms	1.17
	# of changing rings	0.56

Table S7 Summary statistics of product and reaction complexity metrics across datasets and splits for the document-based split.

Metric	Dataset	Split	Mean ± SD	Median	10%	25%	75%	90%
Böttcher Score	CAS	Train	311 ± 181	280	136	194	387	499
		Validation	312 ± 183	282	136	194	387	500
		Test	313 ± 186	282	136	194	388	498
	Pistachio	Train	330 ± 179	307	151	212	414	519
		Validation	326 ± 174	302	150	209	410	518
		Test	332 ± 176	309	152	213	416	522
	USPTO	Train	289 ± 128	275	139	192	369	452
		Validation	289 ± 128	274	139	190	372	458
		Test	290 ± 127	275	139	192	372	457
Spacial Score	CAS	Train	16.7 ± 7.70	14.2	10.5	11.3	19.5	26.3
		Validation	16.7 ± 7.70	14.1	10.5	11.3	19.4	26.0
		Test	16.8 ± 7.71	14.3	10.5	11.3	19.6	26.5
	Pistachio	Train	16.4 ± 6.59	14.9	10.5	11.5	19.2	24.3
		Validation	16.4 ± 6.53	14.9	10.5	11.5	19.2	24.2
		Test	16.5 ± 6.65	15.0	10.5	11.5	19.3	24.3
	USPTO	Train	15.3 ± 5.83	13.7	10.3	11.1	17.8	22.3
		Validation	15.3 ± 5.69	13.8	10.3	11.1	18.0	22.3
		Test	15.5 ± 6.03	13.8	10.3	11.1	17.9	22.4
SAScore	CAS	Train	3.27 ± 1.04	3.13	2.11	2.53	3.80	4.57
		Validation	3.25 ± 1.05	3.11	2.10	2.52	3.79	4.54
		Test	3.28 ± 1.06	3.14	2.11	2.53	3.82	4.60
	Pistachio	Train	3.30 ± 0.90	3.21	2.25	2.67	3.79	4.42
		Validation	3.29 ± 0.89	3.19	2.25	2.66	3.77	4.43
		Test	3.31 ± 0.91	3.22	2.25	2.68	3.80	4.44
	USPTO	Train	3.02 ± 0.74	2.97	2.13	2.49	3.47	3.96
		Validation	3.01 ± 0.73	2.95	2.12	2.48	3.46	3.97
		Test	3.04 ± 0.74	3.00	2.13	2.50	3.48	3.98
NPScore	CAS	Train	-0.46 ± 0.90	-0.52	-1.54	-1.09	0.03	0.66
		Validation	-0.48 ± 0.90	-0.54	-1.53	-1.12	0.01	0.64
		Test	-0.46 ± 0.90	-0.52	-1.53	-1.09	0.03	0.66
	Pistachio	Train	-0.72 ± 0.77	-0.81	-1.60	-1.24	-0.31	0.22
		Validation	-0.72 ± 0.78	-0.80	-1.61	-1.25	-0.30	0.24
		Test	-0.72 ± 0.78	-0.81	-1.60	-1.24	-0.30	0.23
	USPTO	Train	-0.78 ± 0.75	-0.85	-1.65	-1.29	-0.35	0.16
		Validation	-0.77 ± 0.74	-0.85	-1.62	-1.28	-0.36	0.14
		Test	-0.77 ± 0.77	-0.85	-1.65	-1.29	-0.33	0.19
# of reacting atoms	CAS	Train	2.99 ± 2.32	2	1	2	4	6
		Validation	2.97 ± 2.33	2	1	2	4	6
		Test	3.00 ± 2.40	2	1	2	4	6
	Pistachio	Train	2.60 ± 2.26	2	1	2	2	5
		Validation	2.61 ± 2.28	2	1	2	2	5
		Test	2.60 ± 2.25	2	1	2	2	5
	USPTO	Train	2.82 ± 1.83	2	1	2	3	5
		Validation	2.82 ± 1.82	2	1	2	3	5
		Test	2.82 ± 1.85	2	1	2	3	5
# of changing rings	CAS	Train	0.26 ± 0.61	0	0	0	0	1
		Validation	0.25 ± 0.61	0	0	0	0	1
		Test	0.26 ± 0.60	0	0	0	0	1
	Pistachio	Train	0.14 ± 0.47	0	0	0	0	1
		Validation	0.15 ± 0.47	0	0	0	0	1
		Test	0.15 ± 0.47	0	0	0	0	1
	USPTO	Train	0.15 ± 0.43	0	0	0	0	1
		Validation	0.15 ± 0.43	0	0	0	0	1
		Test	0.16 ± 0.44	0	0	0	0	1

S3 Models

In this work, we use five different model architectures: template-relevance (TR), Retrochimera Edit (Neural Loc), augmented transformer (AT), Graph2SMILES (G2S), and Retrochimera DeNovo (R-SMILES 2).

S3.1 Model hyperparameters, training, and additional details

The code for these models is publicly available and can be accessed and deployed via ASKCOS.[1] For G2S and AT, we used the default hyperparameter settings provided in ASKCOS. For the RetroChimera models (NeuralLoc and R-SMILES 2), we used the hyperparameter settings from the authors' published repository: the Pistachio configuration for our Pistachio and CAS splits, and the USPTO-FULL configuration for our USPTO-FULL split.*

Across the template-free models, we observe that no single model dominates across all datasets. While R-SMILES 2 substantially outperforms G2S on USPTO, the two perform comparably on Pistachio, and G2S outperforms R-SMILES 2 on CAS (Figs. 2A, S9A1, A2). This dataset-dependent ordering suggests that the relative performance of template-free models is sensitive to factors beyond the modeling paradigm itself, such as dataset size and the choice of training hyperparameters. We used the published training configurations for each model and did not perform additional per-dataset hyperparameter tuning, so we do not claim any single model to be state-of-the-art across all datasets. Importantly, however, the broader trend is clear: template-free models tend to outperform template-based models across all three datasets.

For TR, model training and hyperparameter optimization followed the approach from previous publication on a single NVIDIA GeForce RTX 4090 GPU.[18,19] The Adam optimizer with early stopping (patience = 2, tolerance = 10^{-4}), a maximum of 150 epochs, and learning rate reduction on plateau (patience = 1, factor = 0.3) was employed. The default values in ASKCOS were used for all hyperparameters unless specifically mentioned above. Hyperparameters were tuned via Bayesian optimization over the following search space using the wandb library[20] library, selecting the set of hyperparameters with the highest validation accuracy (Table S8):

1. Dropout: [0.1, 0.2, 0.3, 0.4, 0.5]
2. Learning rate: [0.0001 – 0.01]
3. Hidden activation: [ELU, ReLU, LeakyReLU, GELU]
4. Number of hidden layers: [1, 2]
5. Hidden units: [512, 1024, 2048]

Table S8 TR model parameters. Output dimension is equal to the number of templates.

	Document-based split			Random split		
	**USPTO**	**Pistachio**	**CAS**	**USPTO**	**Pistachio**	**CAS**
Dropout	0.4	0.5	0.5	0.5	0.3	0.3
Learning Rate ($\times 10^{-3}$)	1.4809	0.6399	0.7100	1.3802	0.9883	0.5958
Hidden Activation	GELU	ELU	ELU	GELU	GELU	LeakyReLU
Number of Hidden Layers	1	2	2	2	1	1
Hidden Sizes	2,048	2,048	2,048	2,048	2,048	2,048
Input Dimension	2,048	2,048	2,048	2,048	2,048	2,048
Output Dimension	16,495	53,275	91,589	16,224	53,441	92,293

As the goal of our work is to examine failure modes, at test time, the TR model employs an *optimistic* ranking strategy: when a template generates multiple precursor sets, the ground-truth precursors (if present) are given highest priority. To reduce computational cost, we limit the number of applied templates to 500, except during our analysis of exhaustive template enumeration.

*https://github.com/microsoft/retrochimera/tree/main/retrochimera/cli/config

S3.2 Discussion on model choice for analysis

In this work, our focus is on characterizing model failure modes across diverse reaction datasets rather than performing an exhaustive benchmark of all existing one-step retrosynthesis models. Accordingly, we limit our analysis to models to TR, NeuralLoc, AT, G2S, and R-SMILES 2. We do not include the analysis with semi-template models, diffusion, or flow-based models in this study, as each framework defines reaction centers differently and is subject to constraints such as the inability to fully handle stereochemistry, and multicenter transformations.

Semi-template models first identify the reaction centers and decompose the target molecule into synthons, and subsequently reconstruct the corresponding reactants. The reconstruction process has been modeled as leaving-group selection[21] or as conditional graph generation conditioned on the predicted synthons.[22] Despite sharing this two-stage framework, these models differ in how they define and handle reaction centers. G2G defines the reaction center as the bond(s) present in the product but absent in the reactants, so it only detects bond-breaking events.[22] Other models extend this concept to include atom-level transformations: for instance, G2Retro[23] broadens the definition to include both bond-type changes and atom center which loses a fragment without other bond changes, and GraphRetro[21] implicitly captures bond and formal charge variations through hydrogen count adjustments on reactive atoms. We do not conduct any comparison because of this inconsistency.

In parallel, diffusion and flow-matching approaches have been applied to one-step retrosynthesis. However, these models often oversimplify stereochemical considerations, either by ignoring chirality and formal charges in molecular representations or by directly transferring chiral tags from the product to the reactants via atom mapping.[24–26] Moreover, some methods further restrict the number of reactive centers to two, limiting their capacity to capture multi-center transformations.[27] Importantly, most of these approaches have been evaluated only on USPTO-50k.[28] While such simplifications may perform adequately on USPTO-50k, which represents a comparatively simple chemical space, these models may fail to generalize to the more complex transformations encountered in broader and more chemically diverse datasets.

S4 Evaluation metrics

Here, we provide details on how each metric is calculated. All chemical structures, both ground truth and predicted sequences, were processed using RDKit. All reactants SMILES are canonicalized with atom-mapping removed before comparison. We note that the top-k accuracies for the stereochemistry-agnostic, synthon, superset, and two-step superset metrics are computed from the top-50 model predictions, and that predictions are deduplicated after metric-specific processing. For example, under the synthon metric, predictions such as R–Cl, R–Br, and R'–Cl all map to the same synthon R; thus R is counted only once (e.g., as top-1), and R' would be counted as the next distinct synthon (e.g., top-2).

- **Superset:** Both the recorded and the predicted precursor(s) are split into individual molecular(s) to form molecular set. A prediction is scored as correct if the set of predicted molecules is a superset of the recorded set.
- **Stereochemistry-agnostic:** Stereochemistry in both the ground-truth and predicted reactant are removed using `rdkit.Chem.RemoveStereoc` The resulting SMILES are then canonicalized and compared for exact matches.
- **Synthon:** Synthon accuracy metric relies on atom mapping. To ensure consistent mapping across the dataset, both the ground truth and predicted reactions were processed using RXNMapper[29]. We then extracted the "synthon" by discarding all unmapped atoms. A new molecular graph was constructed containing only the mapped atoms and their connecting bonds (all type of bonds), while strictly preserving the original stereochemical tags. A prediction is considered correct if the set of canonical SMILES strings derived from these mapped scaffolds is identical to that of the ground truth. To also minimize the influence of erroneous mapping cases, we first verify correctness with respect to the superset metric before extracting the synthons for evaluation.
- **Two-step superset:** If the model fails to predict the correct precursors in the first retrosynthetic step (the "first pass"), the evaluation proceeds to a second step. The largest predicted precursor (by number of heavy atoms) from the first pass becomes the target for a second pass of retrosynthesis. During this second iteration, for the template-relevance model, we exhaustively apply the default list of templates (Section 3.5) to generate all possible precursors, whereas for template-free models, we retain the top-50 model predictions. To ensure a fair comparison against the original starting materials, we reconstruct the full reactant set by combining the predictions from the second step with the smaller fragment(s) from the first step. This reconstructed set is then compared against the original ground truth using the superset accuracy.

We note that these failure modes are not mutually exclusive: different predictions for the same reaction (e.g., appearing at different ranks) may fall into different categories, and a single prediction may simultaneously satisfy multiple criteria. For example, a prediction may differ from the reported precursor in a leaving group, which may be converted to the reported leaving group through a functional group interconversion at the second-pass, thereby contributing to both synthon and two-step superset accuracy (Fig. S7).

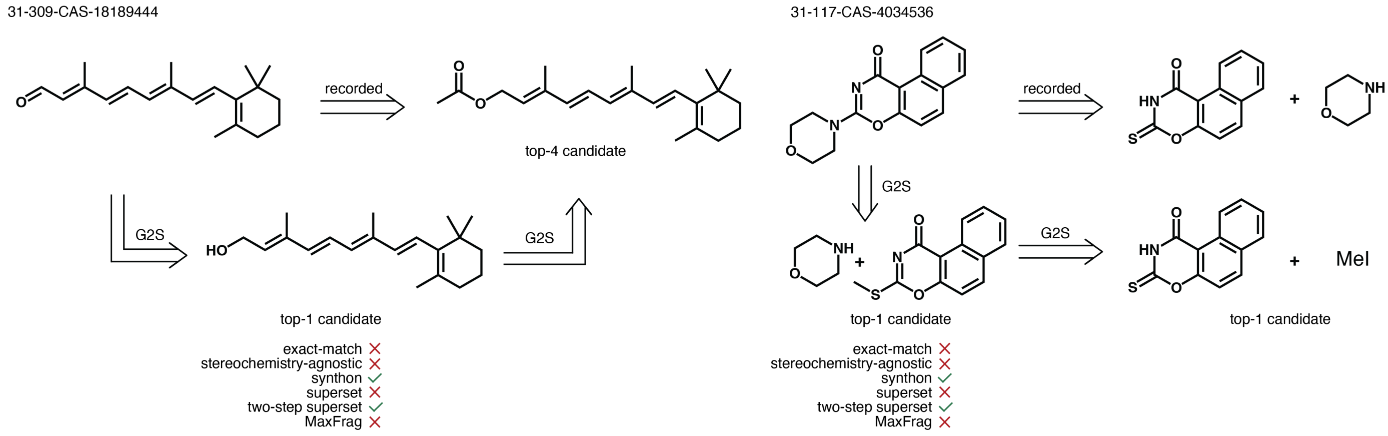

Figure S7 Example where exact-match accuracy is incorrect, but both synthon accuracy and two-step superset accuracy are correct.

S5 Additional Results

S5.1 Random split Results

Fig. S8 shows the exact-match accuracy for both random-split and document-split evaluations across USPTO, Pistachio, and CAS datasets. The results illustrate that random splits tend to give an overly optimistic estimate of model performance. This occurs because reactions reported within the same publication often share closely related transformations and structurally similar products, which inflates the accuracy when such reactions appear in both the training and test sets. In contrast, document-split evaluation provides a better assessment of generalization.

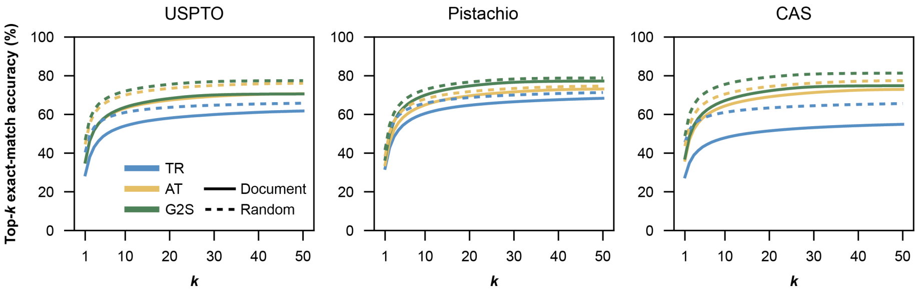

Figure S8 Comparison of exact-match accuracy between document split and random split on USPTO, Pistachio, and CAS.

S5.2 Analysis for USPTO and Pistachio

Here, we present the same analysis as in the main text, applied to the USPTO and Pistachio datasets.

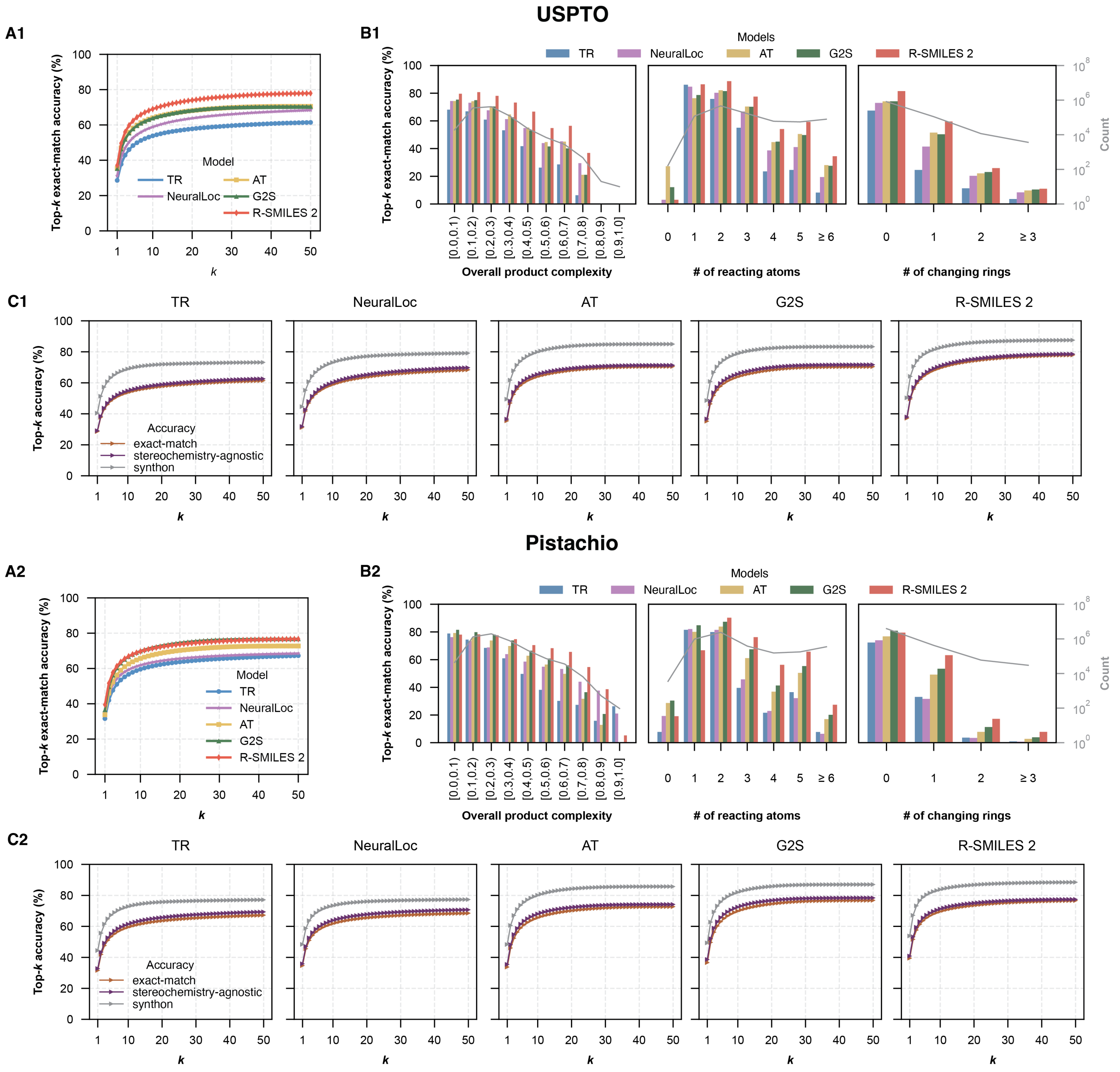

Figure S9 Performance of models evaluated on USPTO (A1, B1, C1) and Pistachio (A2, B2, C3) : (A) Top-*k* exact-match accuracy across all models. (B) Top-50 exact-match accuracies of all models stratified by product and reaction complexity. (C) Top-*k* exact-match, stereochemistry-agnostic, synthon accuracies of all models.

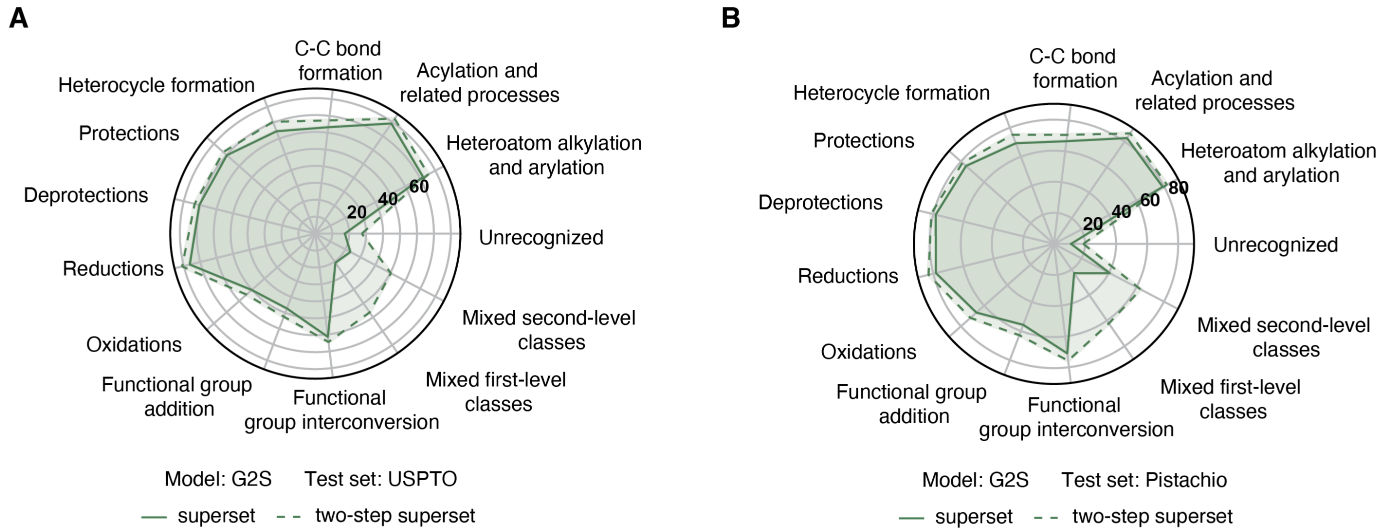

Figure S10 Top-5 superset and two-step superset accuracies stratified by reaction classes of G2S trained and tested on A) USPTO and B) Pistachio.

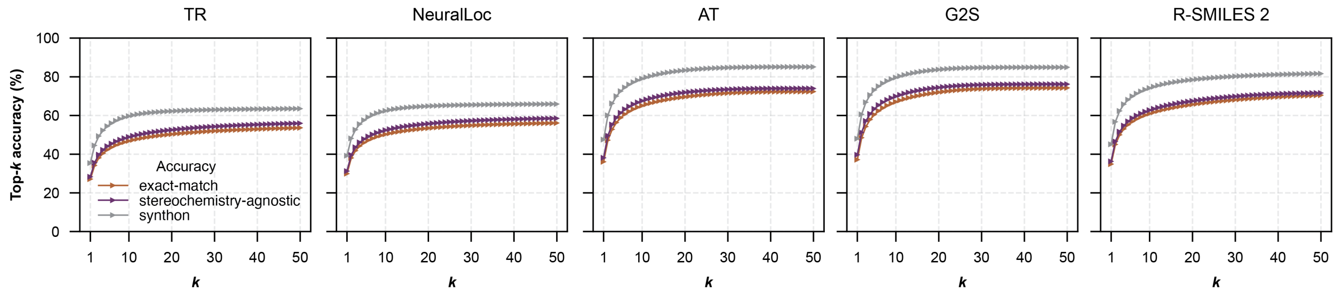

Figure S11 Top-k exact-match, stereochemistry-agnostic, synthon accuracies of all models trained and tested on CAS.

S5.3 Evaluation with complexity-rebalanced dataset

To further test whether the observed underprediction of reaction complexity is driven by model architecture or by class imbalance under maximum-likelihood training, we trained a model on a complexity-rebalanced dataset. The idea is to directly intervene on the training distribution and evaluate whether the bias persists when complex reactions are no longer underrepresented.

For USPTO, we construct the rebalanced training set by grouping reactions according to the number of reacting atoms, restricted to the range 1–6. We cap the range at 6 to reduce potential noise from atom-mapping errors in highly complex reactions. As shown in Tables S3 and S7, reactions with two reacting atoms constitute the dominant class in the original dataset. To mitigate this imbalance, we downsample class 2 and oversample classes 3–6 such that all classes in the 1–6 range have equal frequency. Reactions outside this range are retained without modification.

We then train a model on this rebalanced dataset and compare its behavior to the baseline trained on the original distribution. When evaluated on the original (imbalanced) test set, the rebalanced model shows a shift toward overprediction of reacting atoms, which is expected given the mismatch between training and test distributions and is reflected in a decrease in overall accuracy (Fig. S12A). To control for this effect, we additionally construct a balanced test set with the same class distribution as the rebalanced training set. Even under this matched setting, we still observe a tendency toward underprediction of reacting atoms, indicating that the bias is not fully eliminated by reweighting the training distribution alone. The performance is also stratified by reaction complexity. The balanced model is either slightly worse or at best comparable to the baseline model on more complex reactions (Fig. S12B). This suggests that while oversampling can adjust the model's prediction behavior under distribution shift, it does not fundamentally remove the tendency toward underprediction of complex reactions. The persistence of this trend even under a balanced evaluation setting indicates that the observed bias is not solely driven by class imbalance, and insufficient to remove the bias toward simpler transformations

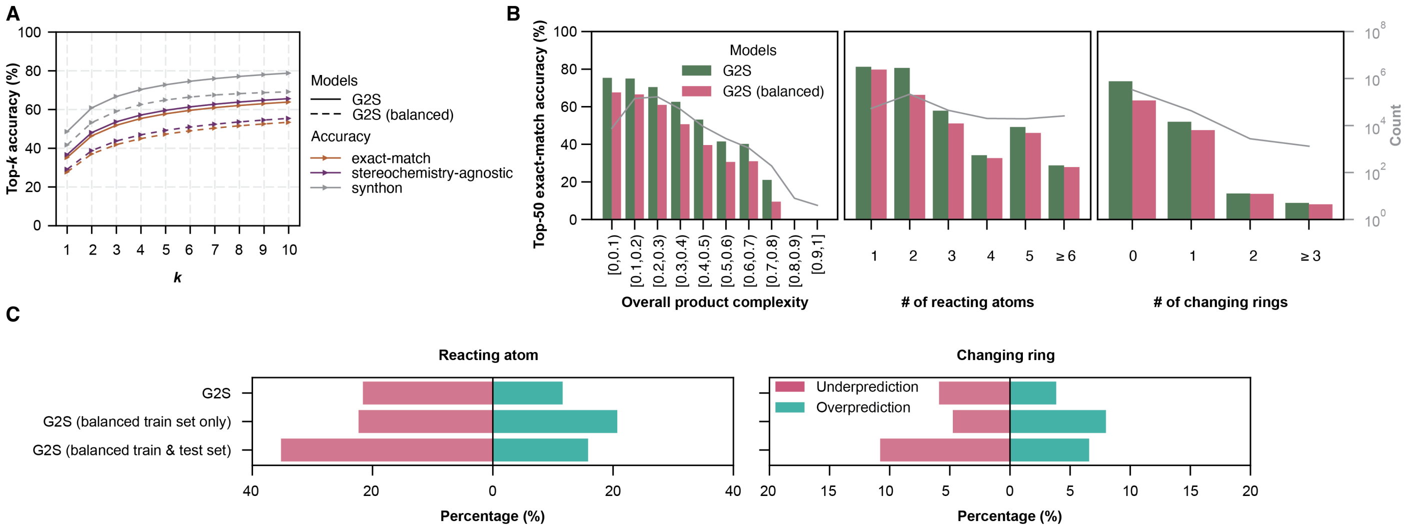

Figure S12 Result of G2S trained on CAS: A) Top-k exact-match, stereochemistry-agnostic, synthon accuracies of G2S trained from same complexity split dataset and trained from that reacting-atom-class-balanced train set. B) Top-50 exact-match accuracies of the 2 models, similiar to A, stratified by product and reaction complexity. C) Under/over-prediction analysis when testing on balanced test and imbalanced test

S5.4 Additional analysis of model underprediction of number of reacting atoms and changing ring

S5.4.1 Left-shift in distributions of number of reacting atoms and changing rings

The distributions of the number of reacting atoms and changing rings in the training and test sets (for both mapping provided) appear visually similar across all datasets. In contrast, the distributions of top-1 model predictions (using RXNMapper) show a pronounced left-shift across all models, indicating a systematic tendency to underpredict these quantities (Fig. S13). We also show that as reaction complexity increases, this trend becomes more pronounced (Figs. S14–S16), consistent with our earlier observation that model performance decreases as reaction complexity increases (Figs. S9, 2).

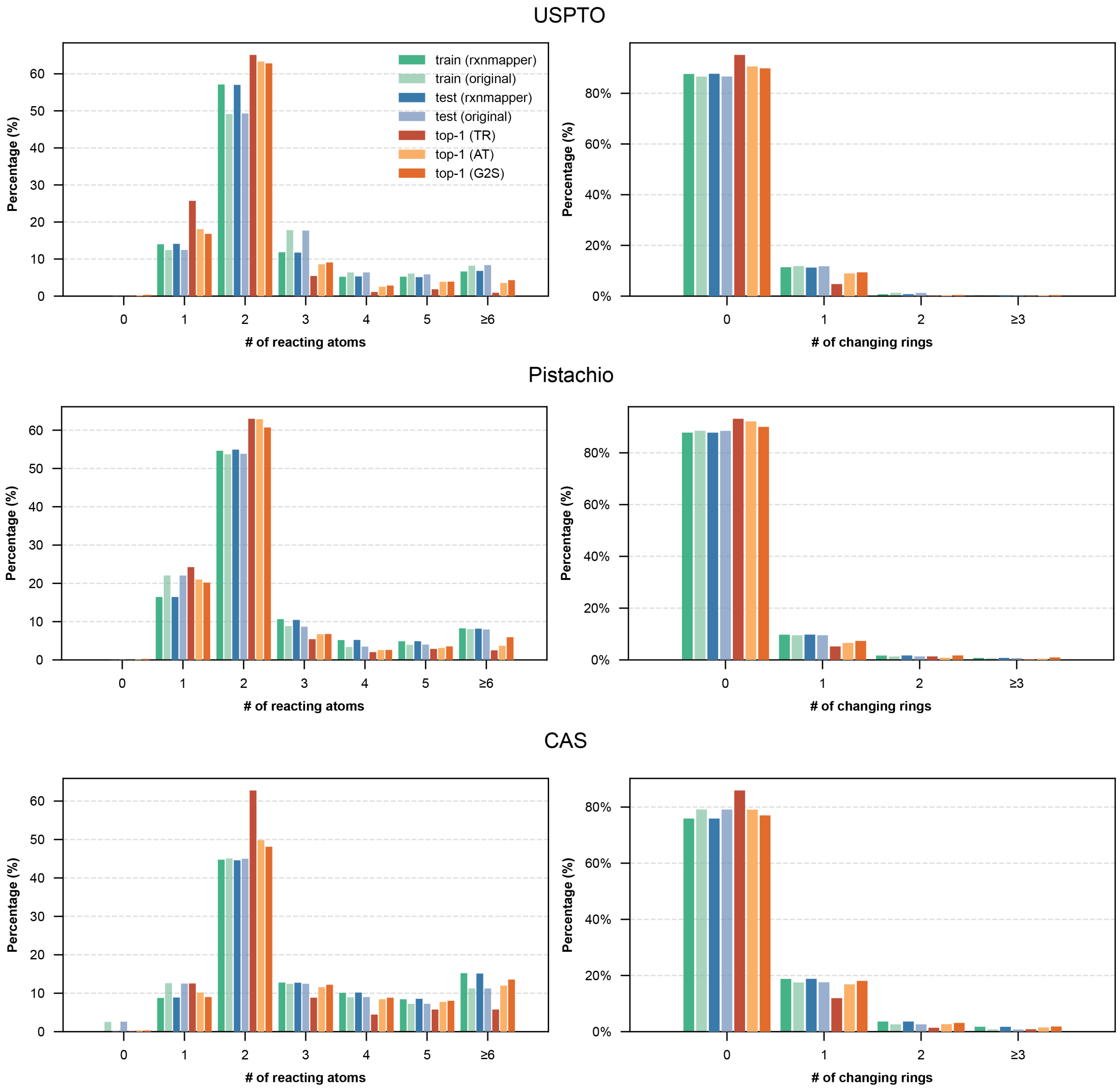

Figure S13 Distribution of the number of reacting atoms and changing rings in the train and test sets (with different mapping), compared with top-1 model predictions across all models and three datasets.

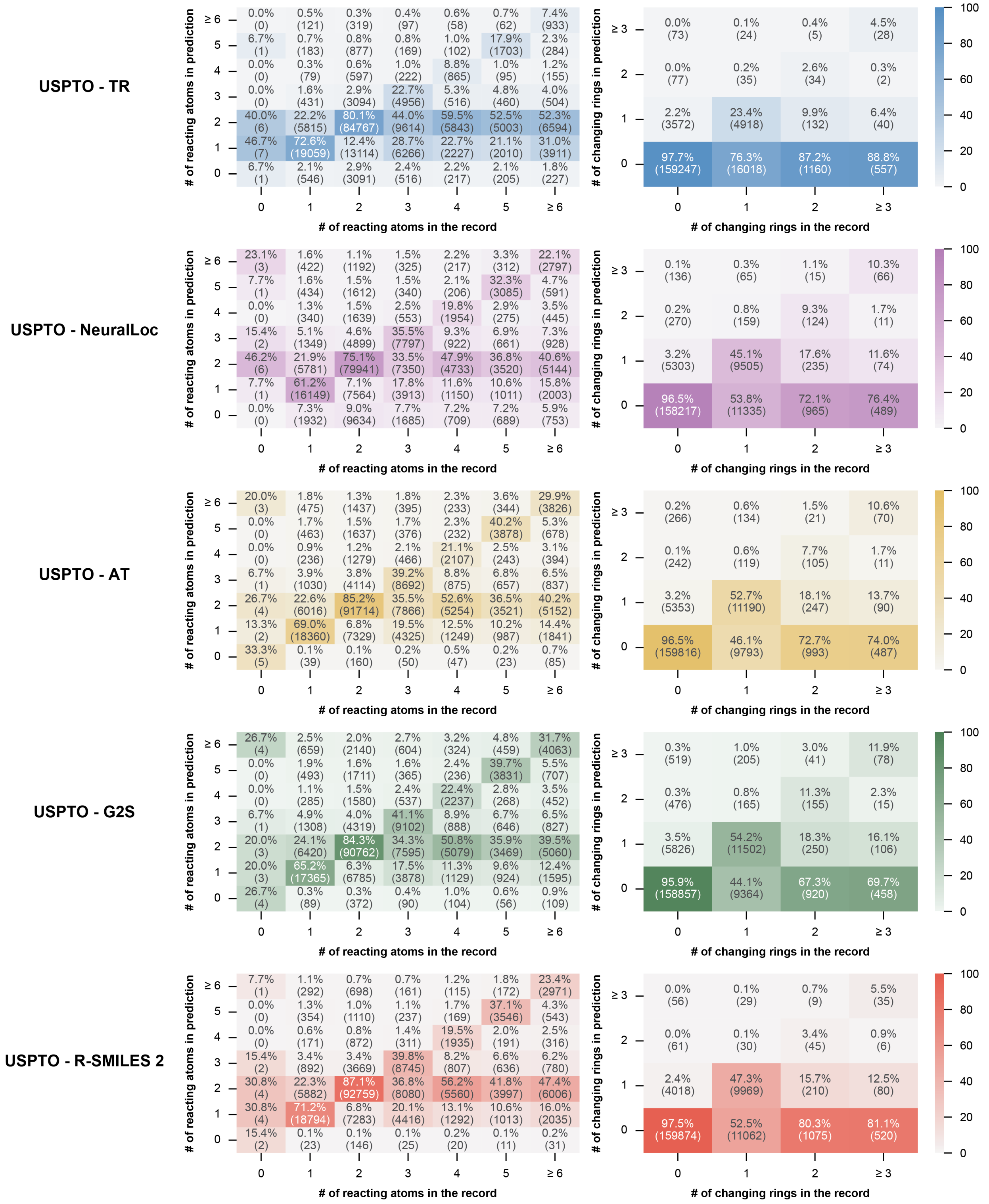

Figure S14 A heatmap of predicted versus recorded values (RXNMapper) for the number of reacting atoms and changing rings illustrates that the model often underpredicts these features for the USPTO dataset. Entries are column-normalized percentages (recorded value on the x-axis), with absolute counts shown in parentheses.

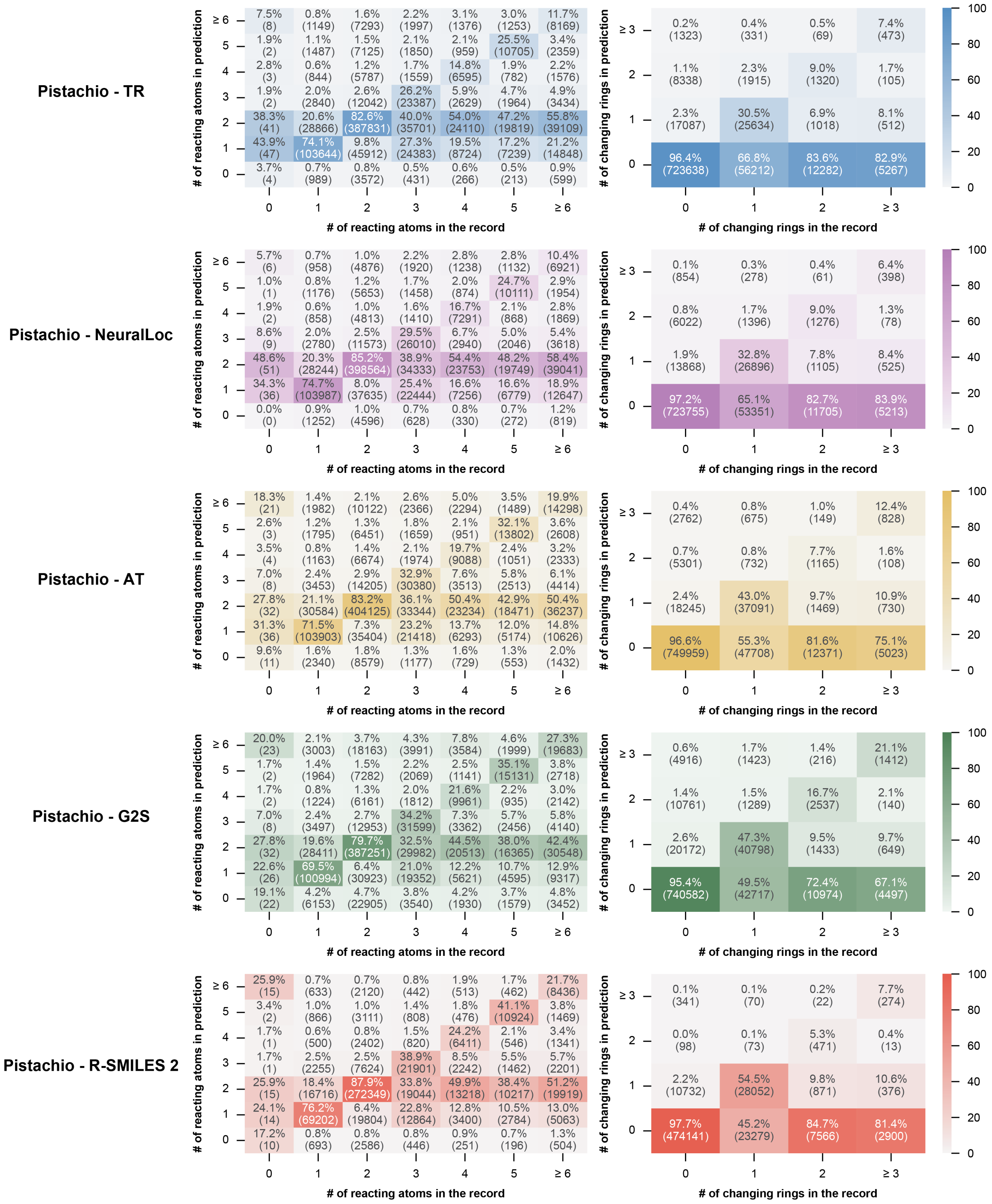

Figure S15 A heatmap of predicted versus recorded values (RXNMapper) for the number of reacting atoms and changing rings illustrates that the model often underpredicts these features for the Pistachio dataset. Entries are column-normalized percentages (recorded value on the x-axis), with absolute counts shown in parentheses.

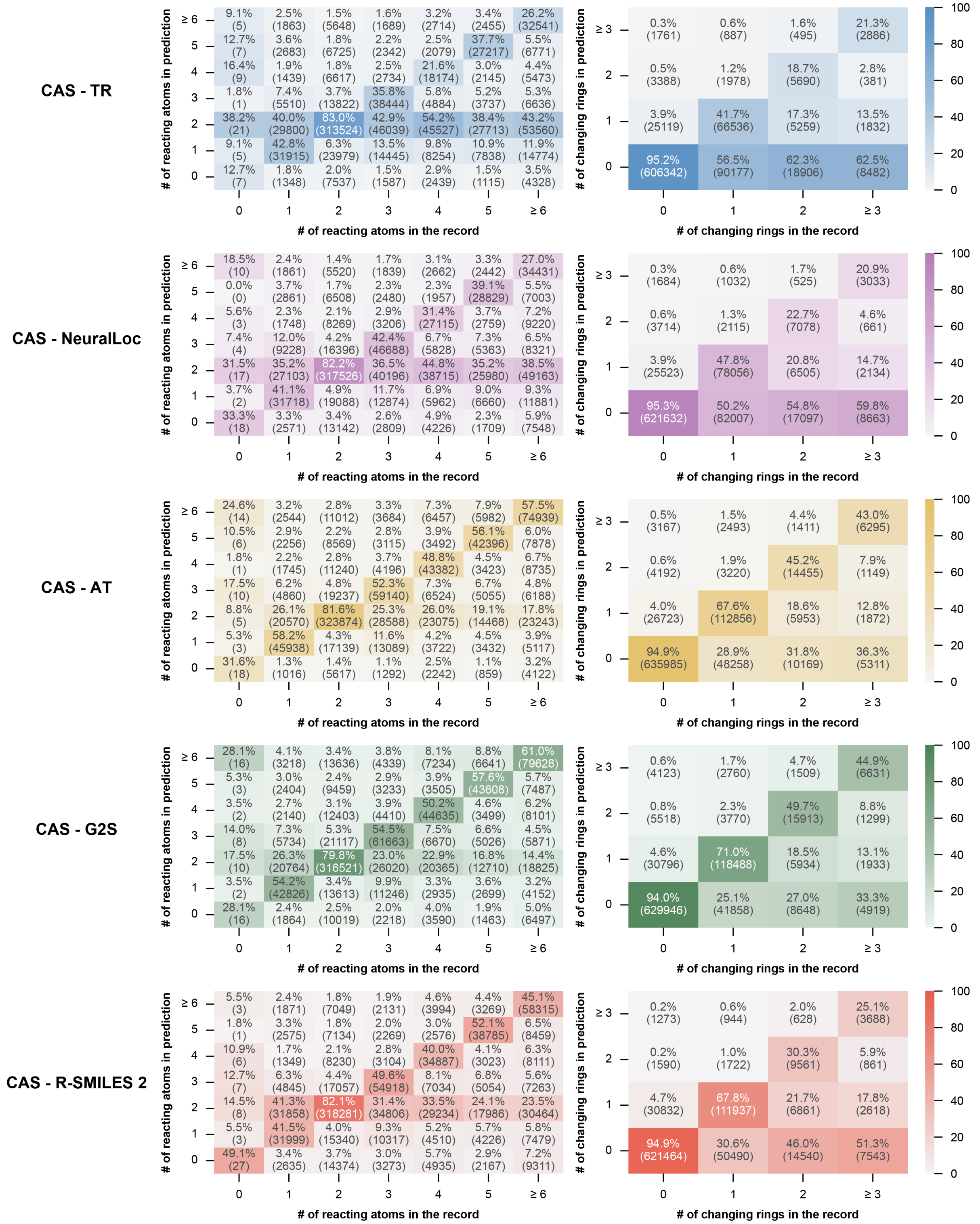

Figure S16 A heatmap of predicted versus recorded values (RXNMapper) for the number of reacting atoms and changing rings illustrates that the model often underpredicts these features for the CAS dataset. Entries are column-normalized percentages (recorded value on the x-axis), with absolute counts shown in parentheses.

S5.4.2 Mapping affects the analysis of the count of reacting atoms and changing rings

Atom mapping is used to determine the number of reacting atoms and changing rings in a chemical reaction. Inconsistent mapping can lead to apparent discrepancies in the number of reacting atoms and changing rings (Figs. S18, S19). Regardless of whether the original database mapping or the RXNMapper-assigned mapping[29] is more chemically accurate, it is important to have a consistent mapping for comparing number of reacting atoms and changing rings of prediction with the ground truth. Because RXNMapper is used to assign atom mappings to the model predictions, we apply the same tool to the ground-truth reactions to ensure a consistent and fair comparison.

We originally did not reassign atom mapping for the datasets, and retain the mappings provided with the data, because each mapping tool has its own imperfections. Across all datasets, the KS distance values for the number of reacting atoms and the number of changing rings using the original mappings (Table S9) are small, indicating that the train and test distributions are closely aligned. When using RXNMapper-assigned mappings, most metrics have similarly small KS distances, suggesting that the distributions remain comparable under alternative mapping assignments. The number of reacting atoms in Pistachio exhibits a larger distance between training and test set, but their distributions remain visually similar (Fig. S13). All other metrics with RXNMapper mapping do not show substantial differences. The distributions of the number of reacting atoms and changing rings in the training and test sets assigned by RXNMapper appear visually similar across all datasets, whereas the distributions of top-1 predictions exhibit a left-shift across all models (Fig. S13).

To verify that the observed left-shift in predictions is not specific to RXNMapper, we repeated the under-/over-prediction analysis using an alternative atom-mapping tool developed by Piotr Grzybowski and coworkers[30]. We remapped both the test-set reactions and their top-1 predictions using this alternative mapper. As shown in Fig. S17, the underprediction trend still holds for both the number of reacting atoms and changing rings relative to the ground truth.

Table S9 KS test statistics comparing complexity metrics between train and test sets across datasets, with the original atom-mapping and the atom-mapping assigned by RXNMapper[29] and Grzybowski's[30] tool.

Dataset	Metric	Mapping	KS statistic ($\times 10^{-3}$)
USPTO	# of reacting atoms	original	1.83
		RXNMapper	1.68
		Grzybowski's	1.44
	# of changing rings	original	0.47
		RXNMapper	0.76
		Grzybowski's	0.66
Pistachio	# of reacting atoms	original	1.00
		RXNMapper	2.65
		Grzybowski's	1.11
	# of changing rings	original	0.58
		RXNMapper	0.28
		Grzybowski's	1.88
CAS	# of reacting atoms	original	1.17
		RXNMapper	1.11
		Grzybowski's	1.11
	# of changing rings	original	0.56
		RXNMapper	0.35
		Grzybowski's	0.89

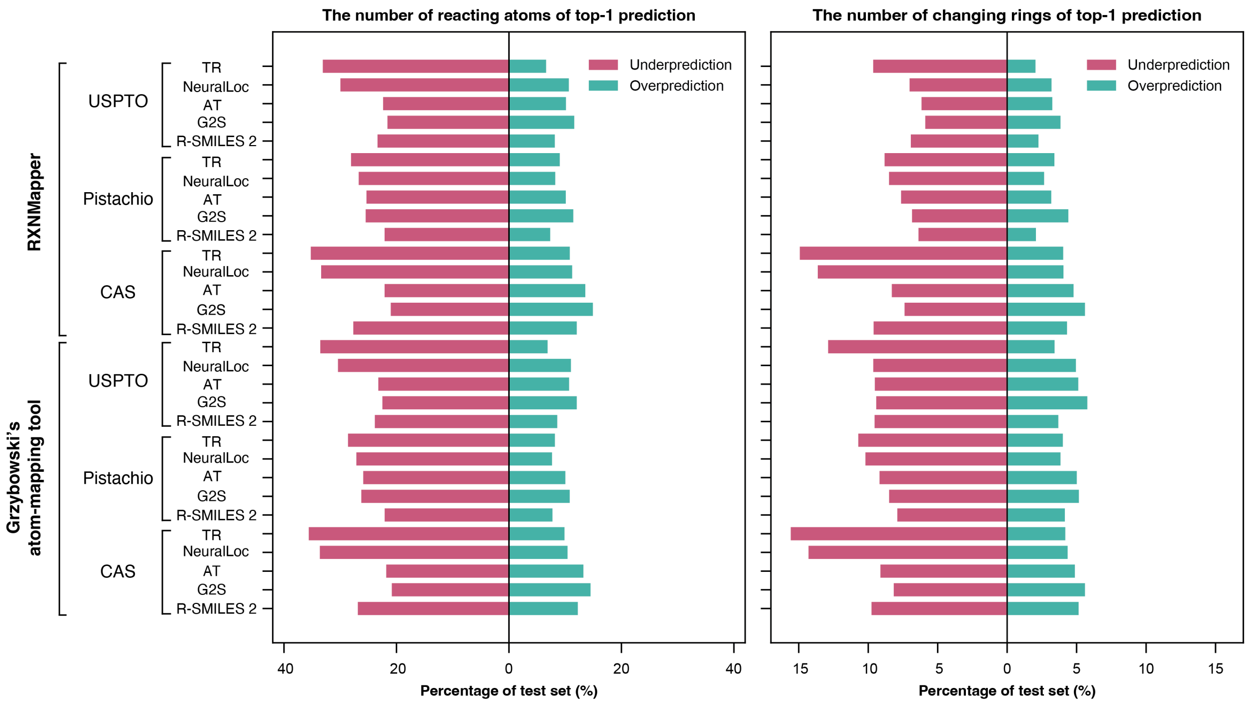

Figure S17 Under- and over-prediction analysis of the number of reacting atoms (left) and changing rings (right) for top-1 predictions across models and datasets using two atom-mapping tools.[29,30]

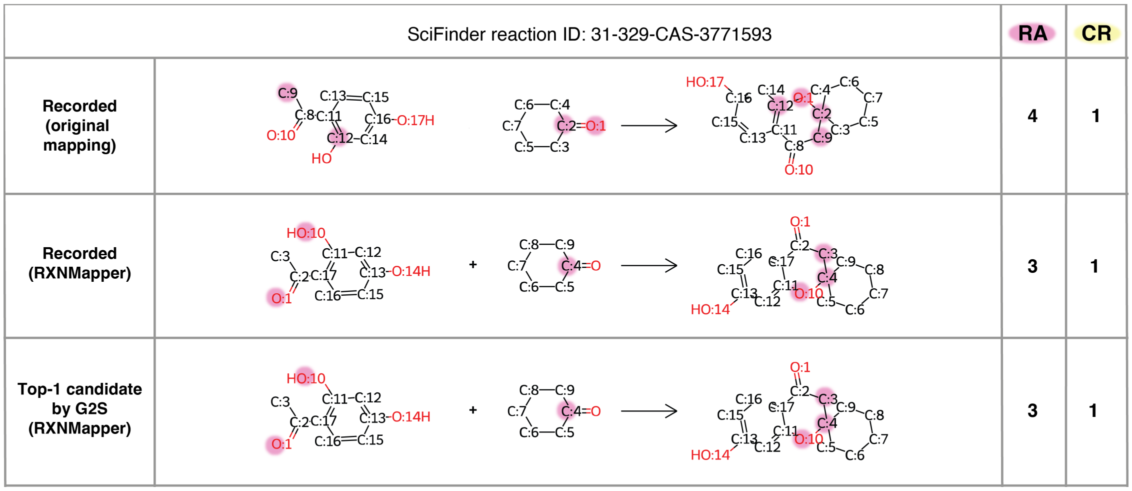

Figure S18 Atom-mapping comparison for a CAS reaction: original mapping (top), RXNMapper mapping (middle), and top-1 G2S candidate (bottom). The original mapping and the RXNMapper-assigned mapping for the recorded reaction differ in the placement of a single oxygen atom, which alters the atom correspondence between reactants and product and leads to a different number of reacting atoms being computed. With the original mapping, the top-1 result is under-predicted; with the RXNMapper mapping, it correctly matches the reacting-atom count.

SciFinder reaction ID: 31-352-CAS-14863365		RA	CR
**Recorded (original mapping)**	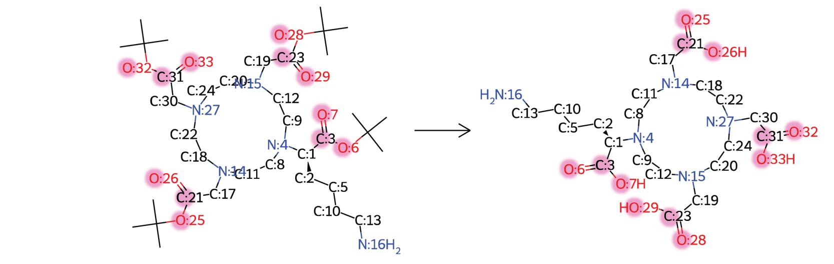	**9**	**0**
**Recorded (RXNMapper)**	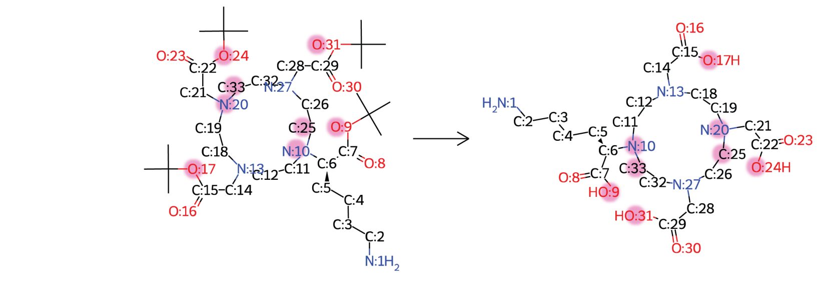	**8**	**0**
**Top-1 candidate by G2S (RXNMapper)**	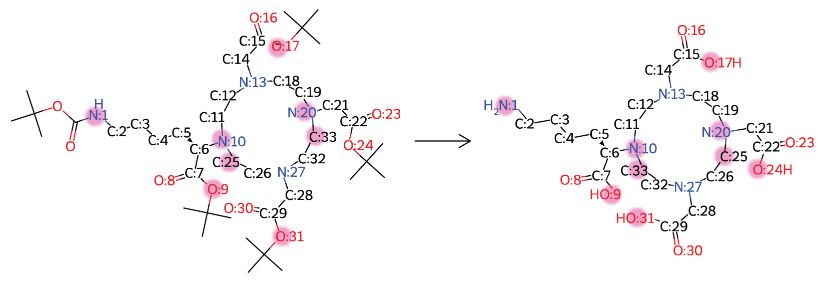	**9**	**0**

Figure S19 Atom-mapping comparison for a CAS reaction: original mapping (top), RXNMapper mapping (middle), and top-1 G2S candidate (bottom). RA: reacting atoms; CR: changing rings. The original recorded mapping misassigns the oxygens on the carboxyl group, and RXNMapper misassigns 2 nitrogens and their neighboring carbons. With the original mapping the top-1 result is correct, whereas with the RXNMapper mapping it is over-predicted.

S5.5 Stratified results by product stereochemistry and stereochemical change during reaction

Our analysis shows that a substantial fraction of the failure modes arise in reactions where there is change in stereochemistry. In these subsets, the model errors are more pronounced. However, these categories collectively represent only a small portion of the full test set, which explains why the corresponding errors lead to only modest changes in the aggregate top-k accuracy (Tables S10, S11). In other words, although stereochemical errors are disproportionately important from a chemical standpoint, their numerical impact on whole-dataset performance remains limited due to the relatively small number of reactions where stereochemical outcomes actually matter.

Table S10 Distribution of R/S changes in USPTO, CAS, and Pistachio datasets.

Dataset	R/S modification	Count	Percentage (%)
USPTO	Unchanged	181,920	96.29
	Inversion	1,901	1.01
	Removal	1,704	0.90
	Addition	2,920	1.55
	Multiple changes	494	0.26
CAS	Unchanged	790,968	88.60
	Inversion	17,176	1.92
	Removal	13,179	1.48
	Addition	66,261	7.42
	Multiple changes	5,163	0.58
Pistachio	Unchanged	837,543	94.67
	Inversion	10,297	1.16
	Removal	11,776	1.33
	Addition	21,187	2.39
	Multiple changes	3,937	0.44

Table S11 Distribution of E/Z changes in USPTO, CAS, and Pistachio datasets.

Dataset	E/Z modification	Count	Percentage (%)
USPTO	Unchanged	186,375	98.64
	Inversion	41	0.02
	Removal	1,586	0.84
	Addition	888	0.47
	Multiple changes	49	0.03
CAS	Unchanged	794,631	89.01
	Inversion	7,119	0.80
	Removal	61,605	6.90
	Addition	24,713	2.77
	Multiple changes	4,679	0.52
Pistachio	Unchanged	868,437	98.16
	Inversion	538	0.06
	Removal	10,273	1.16
	Addition	5,250	0.59
	Multiple changes	242	0.03

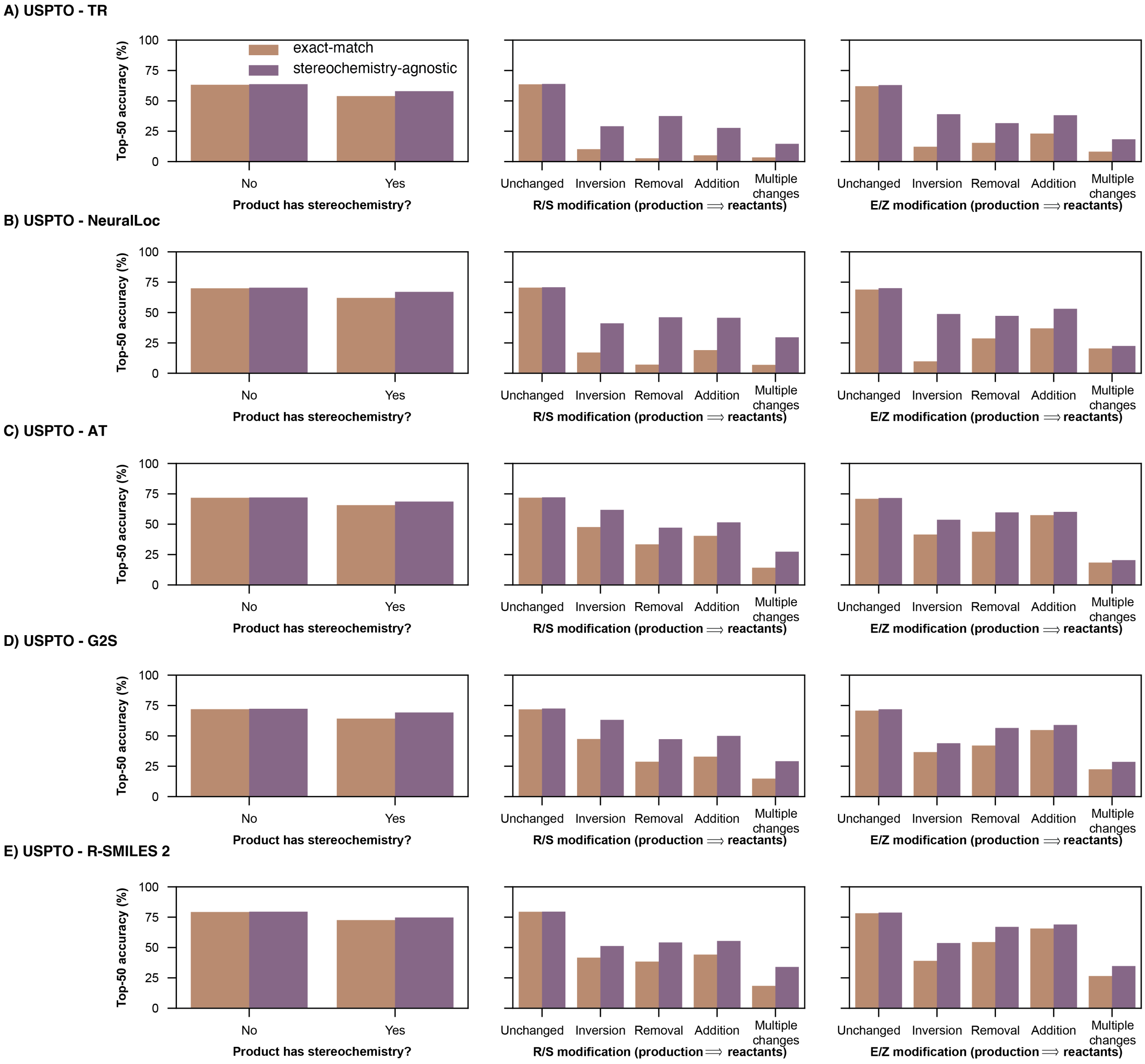

Figure S20 Top-10 exact-match and stereochemistry-agnostic accuracy of models trained and evaluated on USPTO stratified by whether the product contains stereochemistry and whether the reaction involves any changes in R/S or E/Z configuration.

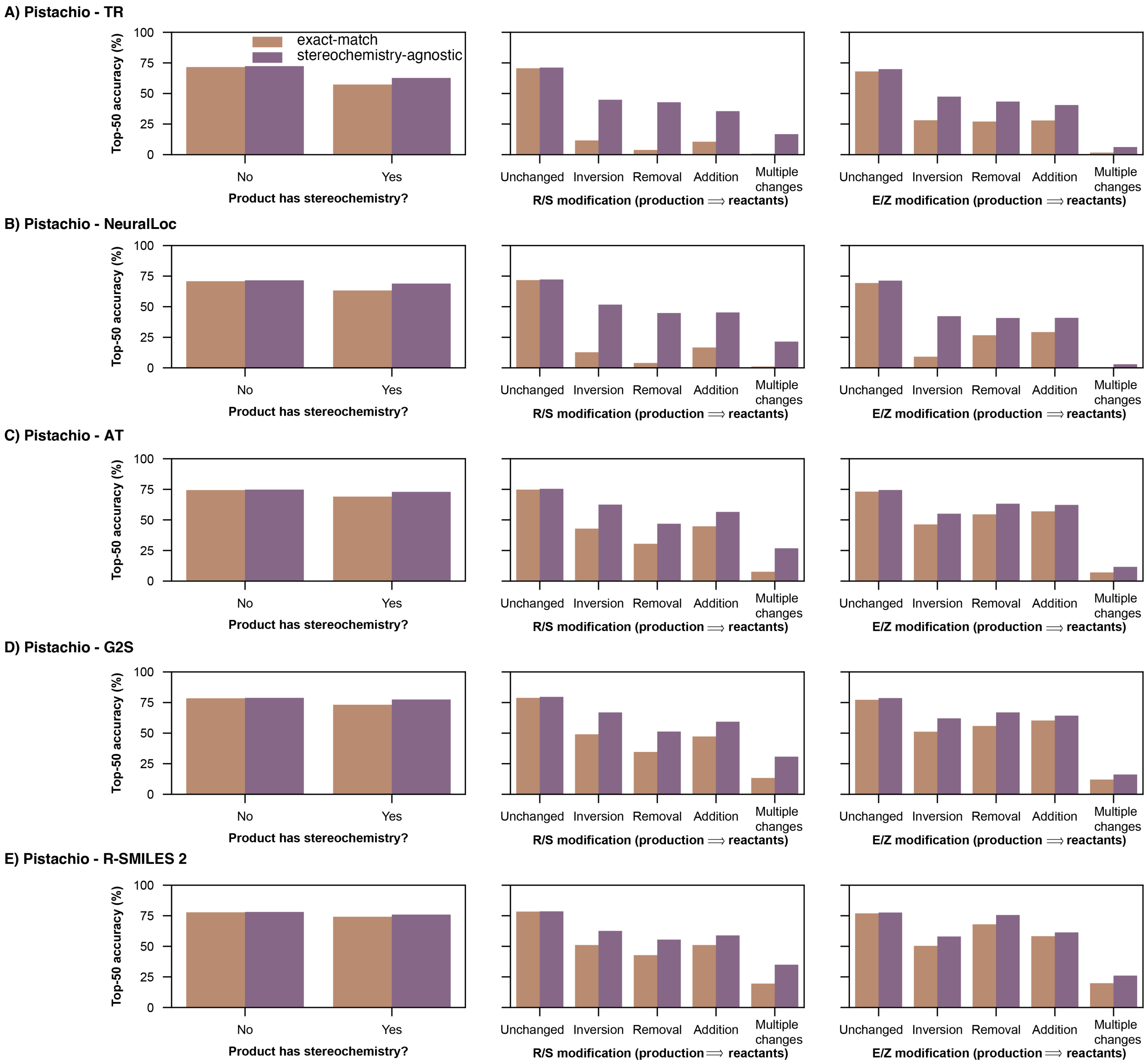

Figure S21 Top-50 exact-match and stereochemistry-agnostic accuracy of models trained and evaluated on Pistachio stratified by whether the product contains stereochemistry and whether the reaction involves a change in stereochemistry (R/S or E/Z) at any carbon atom.

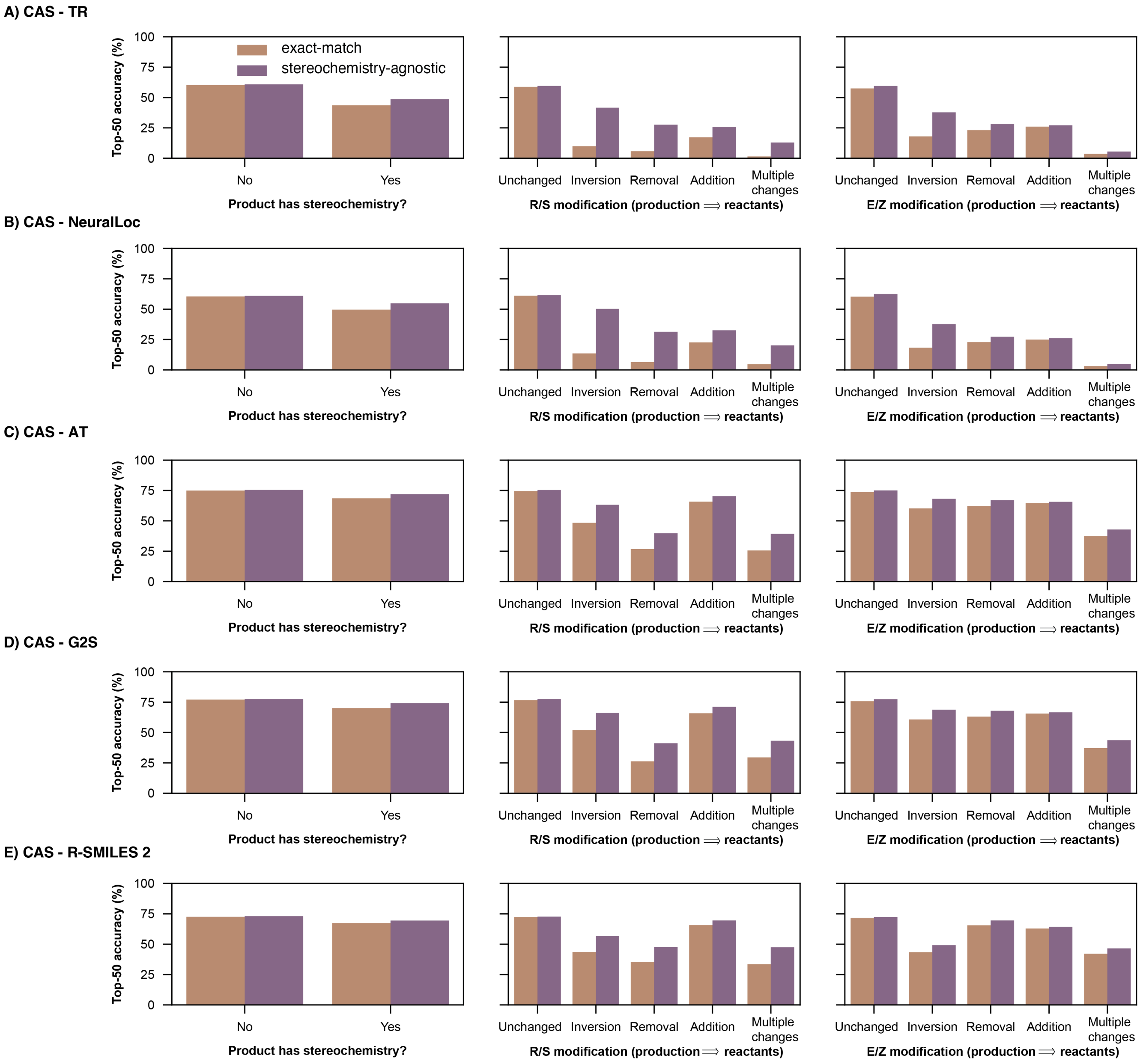

Figure S22 Top-50 exact-match and stereochemistry-agnostic accuracy of models trained and evaluated on CAS stratified by whether the product contains stereochemistry and whether the reaction involves a change in stereochemistry (R/S or E/Z) at any carbon atom.

S5.6 The difference between predicted and recorded leaving groups

Looking at the cases that are incorrect with respect to the superset metric but correct under the synthon metric, we identify several common differences between the leaving groups in the recorded and predicted precursors:

- Halogen mismatch: These are the case that only the halogen differs. Halogen mismatch accounts for 11–15% of this failure mode.
- Positional mismatch: In several examples, the identity (or identities) of the leaving group is predicted correctly, but it is placed on a different reactant than in the ground truth (Fig. S23A). This type of mismatch accounts for 5–10% of all failures in this category and is particularly common for the TR model.
- Similar structural differences: These include minor variations such as methyl vs. ethyl groups (Fig. S23D), bromine vs. chlorine (Fig. S23C), or carboxylic acids vs. their derivatives (Fig. S23B).

Table S12 Percentage of leaving-group positional mismatches and halogen mismatches for top-1 predictions that are evaluated as correct by synthon accuracy but incorrect by superset accuracy. Leaving groups are identified from both ground-truth reactions and model predictions based on atom mappings generated by RXNMapper.

**Model**	**Dataset**	**Percentage (%)**	
		**Positional Mismatch**	**Halogen Mismatch**
TR	USPTO	0.13	13.91
	Pistachio	1.18	13.99
	CAS	5.06	18.59
NeuralLoc	USPTO	0.30	16.48
	Pistachio	1.10	15.95
	CAS	4.97	19.42
AT	USPTO	0.34	14.42
	Pistachio	1.36	15.73
	CAS	5.91	17.84
G2S	USPTO	0.26	14.83
	Pistachio	1.45	15.63
	CAS	6.05	18.22
R-SMILES 2	USPTO	0.19	14.51
	Pistachio	0.88	14.83
	CAS	4.10	19.30

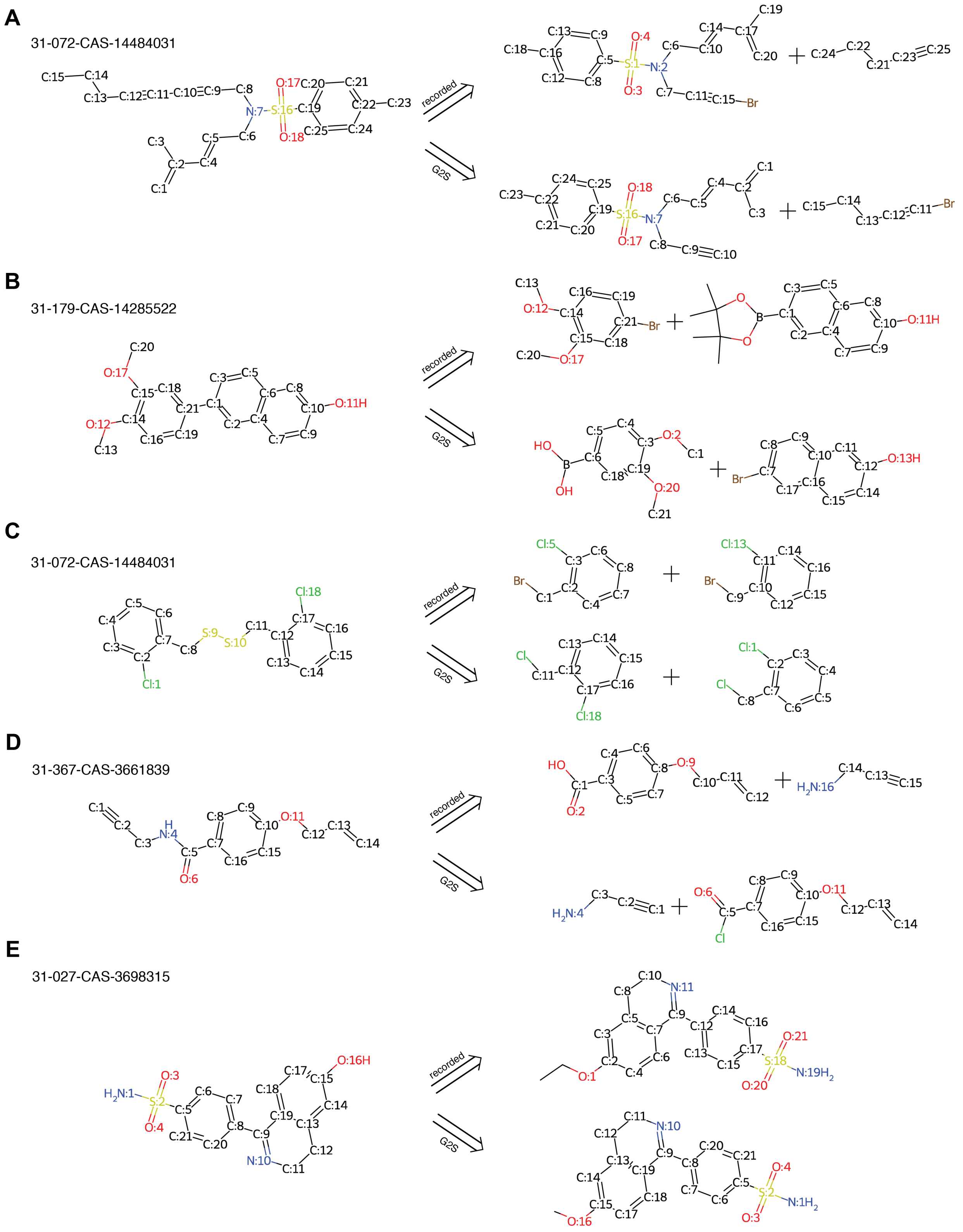

Figure S23 Examples from CAS where G2S predictions fail under the superset metric but are correct under the synthon metric. A) In a Cadiot–Chodkiewicz coupling, the predicted product swaps the bromine between the two reactants. B) In a Suzuki coupling, the predicted reaction swaps the functional groups on the two aryl rings: bromine and boronic acid/ester are exchanged between the phenyl and naphthol rings. C) The recorded reaction uses a bromide, whereas the prediction predicts it with an acyl chloride. D) A carboxylic acid in the recorded reaction is replaced by an acyl chloride in the prediction to form an amide. E) A methyl group in the recorded reaction is replaced by an ethyl group in the prediction.

S5.7 Template enumeration results

S5.7.1 Examples of filtered out templates extending coverage

To better understand why some literature-reported reactions are not recovered in multistep search despite being theoretically accessible, we examined the filtered-out templates (freq 1–4) that provide additional coverage (Figs. S24–S29). These low-frequency templates capture transformations that the model has not seen during training and account for the 5–10% increase in coverage observed when the frequency threshold is removed (Table 1, top-∞, all). These additional templates often correspond to (i) reactions with multiple or distant reaction centers, (ii) uncommon leaving groups encoded directly within the template, or (iii) highly specific structural contexts around the reaction center, such as particular aromatic heterocycles. The latter highlights the inherent trade-off between template generality and specificity in template-based methods: they must balance coverage and accuracy, as more general templates increase applicability but risk chemically implausible disconnections, whereas more specific templates improve prediction accuracy at the cost of broader applicability.

USPTO Template 1

- Number of recovered test set reactions that are not covered by the default template set: 77, Number of reference reactions in the training set: 4
- Reaction SMARTS: [C:1]-[N&H0&+0&D3:7](-[#7:5]-[C:4]=[O&H0&D1:2])-[C&H2&+0&D2:6]-[c:3]>>[C:1]-[N&H1&+0&D2:7]-[#7:5]-[C:4]=[O&H0&D1:2].[c:3]-[C&H2&+0&D2:6]-Br

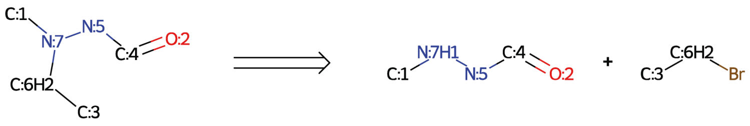

USPTO Template 2

- Number of recovered test set reactions that are not covered by the default template set: 69, Number of reference reactions in the training set: 1
- Reaction SMARTS:

[C:1]-[N&H1&+0&D2:9]-[C:2].[c:3]:[c&H0&+0&D3:10](:[c:4])-[c&H0&+0&D3:11]1:[c:6]:[c:5]:[n:8]:[n:7]:1
>>[C:1]-[N&H0&+0&D3:9](-[C:2])-C(=O)-O-C(-C)(-C)-C.[c:3]:[c&H0&+0&D3:10](:[c:4])-B(-O)-O.[c:5]1:[c:6]:[c&H0&+0&D3:11](:[n:7]:[n:8]:1)-O-S(=O)(=O)-C(-F)(-F)-F

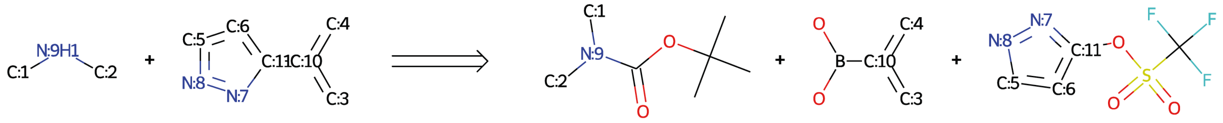

USPTO Template 3

- Number of recovered test set reactions that are not covered by the default template set: 58, Number of reference reactions in the training set: 1
- Reaction SMARTS:

[C:1]-[c&H0&+0&D3:10]1:[c&H1&+0&D2:7]:[c&H0&+0&D3:9](:[n&H0&+0&D2:4]:[n&H0&+0&D2:3]:[c&H0&+0&D3:8]:1-[C:2])-[c&H0&+0&D3:11](:[c:5]):[c:6]>>
[C:1]-[C&H0&+0&D3:10](-[C&H0&+0&D3:8](-[C:2])=O)=O.[N&H2&+0&D1:3]-[N&H2&+0&D1:4].[c:5]:[c&H0&+0&D3:11](:[c:6])-[C&H0&+0&D3:9](-[C&H2&+0&D2:7]-P(=O)(-O-C)-O-C)=O

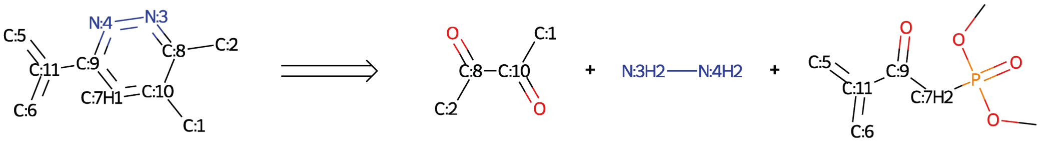

USPTO Template 4

- Number of recovered test set reactions that are not covered by the default template set: 50, Number of reference reactions in the training set: 2
- Reaction SMARTS:

[C&H2&+0&D2:1](-[C&H2&+0&D2:6]-[C:5]=[O&H0&D1:2])-[C&H0&+0&D3:7](=[O&H0&D1:3])-[c:8]:[n:4]
>>[C&H2&+0&D1:1]=[C&H1&+0&D2:6]-[C:5]=[O&H0&D1:2].[O&H0&D1:3]=[C&H1&+0&D2:7]-[c:8]:[n:4]

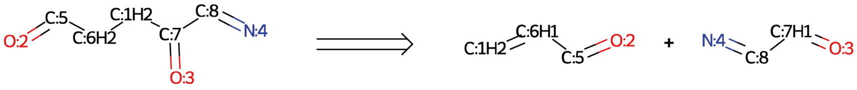

USPTO Template 5

- Number of recovered test set reactions that are not covered by the default template set: 45, Number of reference reactions in the training set: 3
- Reaction SMARTS: [O&H0&+0&D2:1](-[c:2])-[c&H0&+0&D3:5](:[c:3]):[n:4]>>[O&H1&+0&D1:1]-[c:2].[c:3]:[c&H0&+0&D3:5](:[n:4])-I

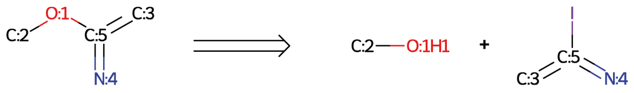

Figure S24 Top five filtered-out templates from the USPTO dataset, ranked by the number of test reactions they recover that would otherwise be failure cases (i.e., not recovered by the default template set). Each entry shows the number of such test reactions, the number of reference reactions in the training set (frequency), and the corresponding reaction SMARTS. Non-zero hydrogen counts are included for any atom for which they are explicitly specified in the reaction SMARTS.

USPTO Template 6

- Number of recovered test set reactions that are not covered by the default template set: 36, Number of reference reactions in the training set: 3
- Reaction SMARTS:

[O&H0&D1:1]=[C&H0&+0&D3:7](-[c:5]:[c:6]:[n:3])-[c&H0&+0&D3:8](:[c:2]):[n:4]
>>[O&H0&D1:1]=[C&H0&+0&D3:7](-[c:5]:[c:6]:[n:3])-N(-C)-O-C.[c:2]:[c&H0&+0&D3:8](:[n:4])-Br

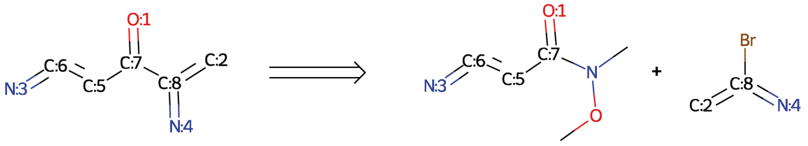

USPTO Template 7

- Number of recovered test set reactions that are not covered by the default template set: 32, Number of reference reactions in the training set: 3
- Reaction SMARTS: [C:1]-[N&H1&+0&D2:2]-[C&H1&+0&D3:5](-[c:3])-[c:4]>>[C:1]-[N&H2&+0&D1:2].[c:3]-[C&H0&+0&D3:5](-[c:4])=O

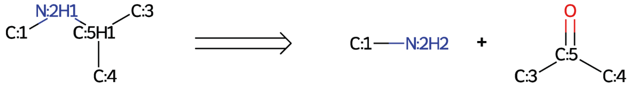

USPTO Template 8

- Number of recovered test set reactions that are not covered by the default template set: 31, Number of reference reactions in the training set: 2
- Reaction SMARTS:

[C:1]-[C&H1&+0&D3:7](-[C:5]=[O&H0&D1:3])-[C&H0&+0&D3:6](=[O&H0&D1:2])-[N&H1&+0&D2:8]-[c:4]
>>[C:1]-[C&H2&+0&D2:7]-[C:5]=[O&H0&D1:3].[O&H0&D1:2]=[C&H0&+0&D2:6]=[N&H0&+0&D2:8]-[c:4]

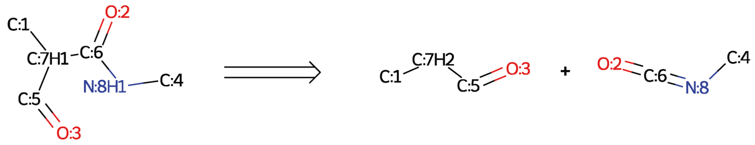

USPTO Template 9

- Number of recovered test set reactions that are not covered by the default template set: 29, Number of reference reactions in the training set: 2
- Reaction SMARTS:

[C:1]-[c&H0&+0&D3:10]1:[n&H0&+0&D3:3](:[c&H0&+0&D3:9](:[c&H1&+0&D2:8]:[c&H0&+0&D3:11]:1-[C:2])-[c&H0&+0&D3:13](:[c:6]):[c:7])-[c&H0&+0&D3:12](:[c:4]):[c:5]
>>[C:1]-[C&H0&+0&D3:10](-[C&H1&+0&D3:11](-[C:2])-[C&H2&+0&D2:8]-[C&H0&+0&D3:9](-[c&H0&+0&D3:13](:[c:6]):[c:7])=O)=O.[N&H2&+0&D1:3]-[c&H0&+0&D3:12](:[c:4]):[c:5]

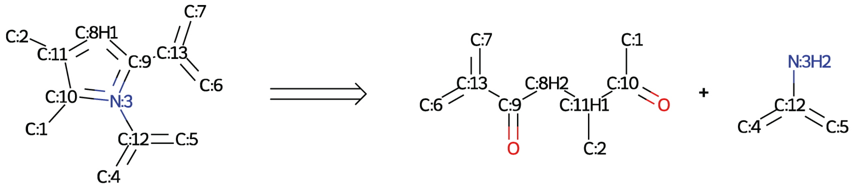

USPTO Template 10

- Number of recovered test set reactions that are not covered by the default template set: 29, Number of reference reactions in the training set: 1
- Reaction SMARTS: [N&H1&+0&D2:1](-[c:2])-[c&H0&+0&D3:5](:[n:3]):[n:4]>>[N&H2&+0&D1:1]-[c:2].[n:3]:[c&H0&+0&D3:5](:[n:4])-O-S(=O)(=O)-C(-F)(-F)-F

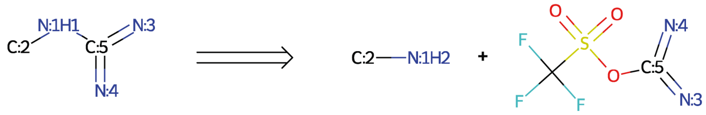

Figure S25 Filtered-out USPTO templates ranked six through ten by the number of test reactions recovered, with the same information reported as in Fig. S24.

Pistachio Template 1

- Number of recovered test set reactions that are not covered by the default template set: 180, Number of reference reactions in the training set: 1
- Reaction SMARTS:

[O&H0&D1:1]=[C:6]-[O&H1&+0&D1:7].[c:2]:[c&H0&+0&D3:9](:[c:3])-[c&H0&+0&D3:8](:[c:4]):[n:5]
>>[O&H0&D1:1]=[C:6]-[O&H0&+0&D2:7]-C.[c:2]:[c&H0&+0&D3:9](:[c:3])-Br.[c:4]:[c&H0&+0&D3:8](:[n:5])-Br

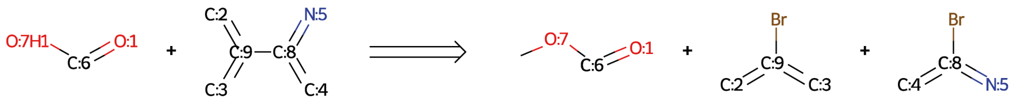

Pistachio Template 2

- Number of recovered test set reactions that are not covered by the default template set: 178, Number of reference reactions in the training set: 1
- Reaction SMARTS:

[n&H0&+0&D2:1]1:[c&H0&+0&D3:7](:[n&H1&+0&D2:2]:[n&H0&+0&D2:8]:[c&H0&+0&D3:9]:1-[c&H0&+0&D3:10](:[c:5]):[n:6])-[c&H0&+0&D3:11](:[c:3]):[c:4]
>>[N&H1&+0&D1:1]=[C&H0&+0&D3:9](-[N&H1&+0&D2:8]-[N&H2&+0&D1:2])-[c&H0&+0&D3:10](:[c:5]):[n:6].[c:3]:[c&H0&+0&D3:11](:[c:4])-[C&H1&+0&D2:7]=O

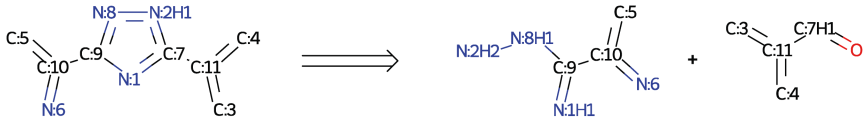

Pistachio Template 3

- Number of recovered test set reactions that are not covered by the default template set: 171, Number of reference reactions in the training set: 1
- Reaction SMARTS:

[O&H0&D1:1]=[c&H0&+0&D3:9]1:[n&H0&+0&D3:6](:[c&H1&+0&D2:5]:[c:8]:[n&H0&+0&D3:7]:1:[c:4])-[c&H0&+0&D3:10](:[c:2]):[c:3]
>>[O&H0&D1:1]=[C&H0&+0&D3:9](-O-C(-Cl)(-Cl)-Cl)-O-C(-Cl)(-Cl)-Cl.[c:2]:[c&H0&+0&D3:10](:[c:3])-[N&H1&+0&D2:6]-[C&H2&+0&D2:5]-[c:8]:[n&H0&+0&D2:7]:[c:4]

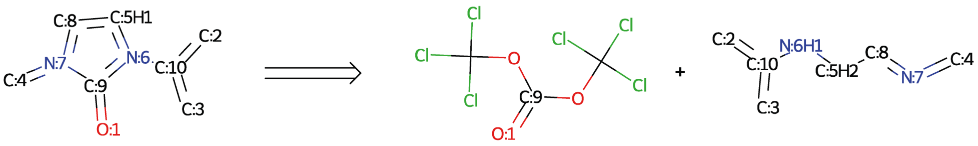

Pistachio Template 4

- Number of recovered test set reactions that are not covered by the default template set: 91, Number of reference reactions in the training set: 1
- Reaction SMARTS:

[C:1]-[N&H0&+0&D3:11](-[#7:9]-[C:8]=[O&H0&D1:4])-[C&H2&+0&D2:10]-[c:7].[C:2]#[C&H0&+0&D2:3]-[c&H0&+0&D3:12](:[c:5]):[c:6]
>>[C:1]-[N&H1&+0&D2:11]-[#7:9]-[C:8]=[O&H0&D1:4].[C:2]#[C&H1&+0&D1:3].[c:5]:[c&H0&+0&D3:12](:[c:6])-I.[c:7]-[C&H1&+0&D2:10]=O

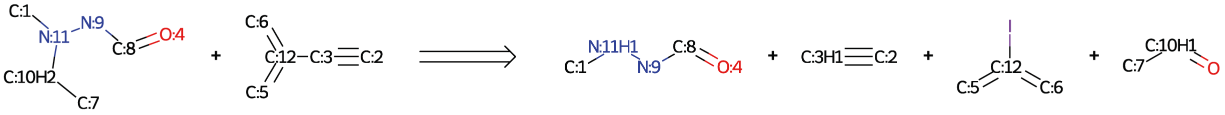

Pistachio Template 5

- Number of recovered test set reactions that are not covered by the default template set: 76, Number of reference reactions in the training set: 1
- Reaction SMARTS: [C:1]-[C&H1&+0&D3:5](-[N&H1&+0&D2:2]-[c:3])-[c:4]>>[C:1]-[C&H1&+0&D3:5](-[c:4])-Cl.[N&H2&+0&D1:2]-[c:3]

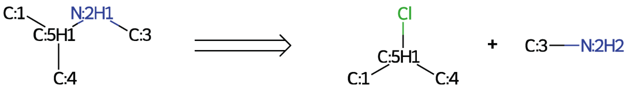

Figure S26 Top five filtered-out templates from the USPTO dataset, ranked by the number of test reactions they recover that would otherwise be failure cases (i.e., not recovered by the default template set). Each entry shows the number of such test reactions, the number of reference reactions in the training set (frequency), and the corresponding reaction SMARTS. Non-zero hydrogen counts are included for any atom for which they are explicitly specified in the reaction SMARTS.

Pistachio Template 6

- Number of recovered test set reactions that are not covered by the default template set: 72, Number of reference reactions in the training set: 1
- Reaction SMARTS: [N&H0&+0&D2:1](=[c:5]:[n:4])-[C&H0&+0&D3:6](=[O&H0&D1:2])-[c:3]>>[N&H1&+0&D1:1]=[c:5]:[n:4].[O&H0&D1:2]=[C&H0&+0&D3:6](-[c:3])-Cl

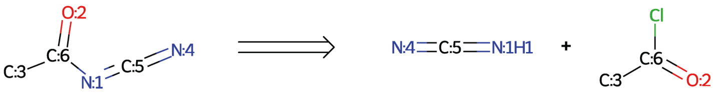

Pistachio Template 7

- Number of recovered test set reactions that are not covered by the default template set: 71, Number of reference reactions in the training set: 3
- Reaction SMARTS:

[C:1]-[N&H1&+0&D2:2]-[C&H0&+0&D3:8]1=[C:4]-[#16:5]-[#7:6]-[C:7]-1=[O&H0&D1:3]
>>[C:1]-[N&H2&+0&D1:2].[O&H0&D1:3]=[C:7]1-[#7:6]-[#16:5]-[C:4]=[C&H0&+0&D3:8]-1-Cl

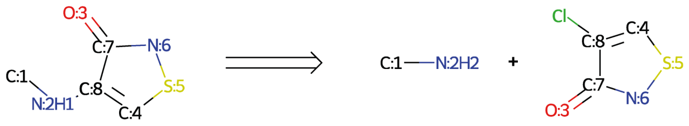

Pistachio Template 8

- Number of recovered test set reactions that are not covered by the default template set: 71, Number of reference reactions in the training set: 1
- Reaction SMARTS:

[n:1]:[n:8]1:[c:5]:[n&H1&+0&D2:3]:[c:6]:[c:4]:[c&H0&+0&D3:7]:1=[O&H0&+0&D1:2]
>>[n:1]:[n:8]1:[c:5]:[n&H0&+0&D2:3]:[c:6]:[c:4]:[c&H0&+0&D3:7]:1-[O&H0&+0&D2:2]-C

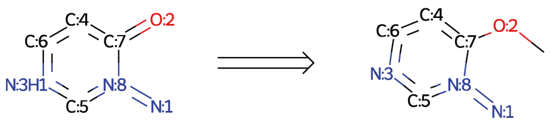

Pistachio Template 9

- Number of recovered test set reactions that are not covered by the default template set: 64, Number of reference reactions in the training set: 1
- Reaction SMARTS: [c:1]:[n&H0&+0&D3:3](:[c:4]:[n:2])-[C&H0&+0&D3]>>[c:1]:[n&H1&+0&D2:3]:[c:4]:[n:2]

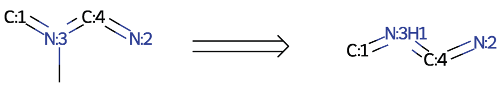

Pistachio Template 10

- Number of recovered test set reactions that are not covered by the default template set: 63, Number of reference reactions in the training set: 4
- Reaction SMARTS:

[C:1]-[N&H1&+0&D2:3]-[C&H0&+0&D3:10](=[O&H0&D1:4])-[C:7]-[#7:2].[C:5](-[C:6]-[O&H1&+0&D1:8])-[O&H1&+0&D1:9]
>>[C:1]-[N&H2&+0&D1:3].[#7:2]-[C:7]-[C&H0&+0&D3:10](=[O&H0&D1:4])-O.[C:5]1-[C:6]-[O&H0&+0&D2:8]-C(-[O&H0&+0&D2:9]-1)(-C)-C

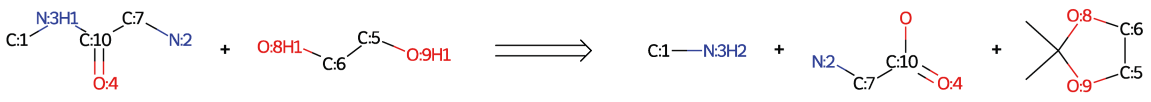

Figure S27 Filtered-out Pistachio templates ranked six through ten by the number of test reactions recovered, with the same information reported as in Fig. S26.

CAS Template 1

- Number of recovered test set reactions that are not covered by the default template set: 181, Number of reference reactions in the training set: 3
- Reaction SMARTS: [C:1]-[C&H1&+0&D3:3](-[#7:2])-[O&H1&+0&D1]>>[C:1]-[C&H2&+0&D2:3]-[#7:2]

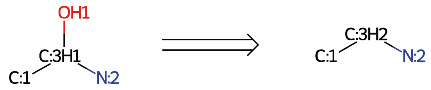

CAS Template 2

- Number of recovered test set reactions that are not covered by the default template set: 133, Number of reference reactions in the training set: 2
- Reaction SMARTS: [#16:1]-[N&H0&+0&D2:3]=[C&H0&+0&D3:5](-[N&H2&+0&D1:2])-[c:4]>>[#16:1]-[N&H2&+0&D1:3].[N&H0&+0&D1:2]#[C&H0&+0&D2:5]-[c:4]

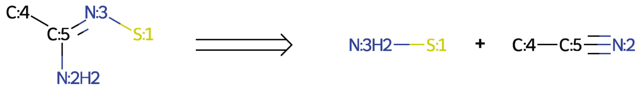

CAS Template 3

- Number of recovered test set reactions that are not covered by the default template set: 111, Number of reference reactions in the training set: 2
- Reaction SMARTS: [C:1]-[N&H1&+0&D2:3]-[O&H1&+0&D1:2]>>[C:1]-[N&H0&+0&D3:3](-[O&H0&+0&D2:2]-C(=O)-O-C(-C)(-C)-C)-C(=O)-O-C(-C)(-C)-C

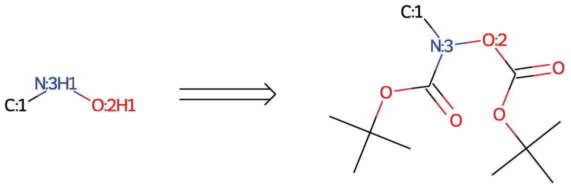

CAS Template 4

- Number of recovered test set reactions that are not covered by the default template set: 102, Number of reference reactions in the training set: 1
- Reaction SMARTS: [C:1]-[C&H0&+0&D3:3](-[O&H1&+0&D1:2])=[O&H0&+0&D1]>>[C:1]-[C&H0&+0&D3:3](=[O&H0&+0&D1:2])-O-C(-C)-C

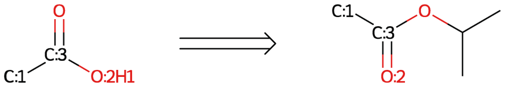

CAS Template 5

- Number of recovered test set reactions that are not covered by the default template set: 100, Number of reference reactions in the training set: 4
- Reaction SMARTS:

[C:1]=[#7:9]-[C@@&H1&+0&D3:13](-[N&H1&+0&D2:3]-[C&H0&+0&D3:12](=[O&H0&D1:5])-[C:7]-[C:8]-[C&H0&+0&D3:11](=[O&H0&D1:6])-[N&H2&+0&D1])-[C:10](-[#7:2])=[O&H0&D1:4]>>[C:1]=[#7:9]-[C&H1&+0&D3:13](-[N&H2&+0&D1:3])-[C:10](-[#7:2])=[O&H0&D1:4].[O&H0&D1:5]=[C&H0&+0&D3:12](-[C:7]-[C:8]-[C&H0&+0&D3:11](=[O&H0&D1:6])-O-C(-C)(-C)-C)-O

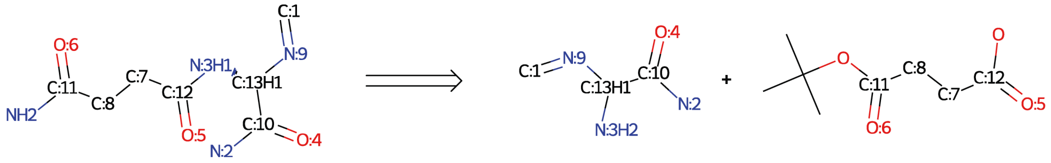

Figure S28 Top five filtered-out templates from the CAS dataset, ranked by the number of test reactions they recover that would otherwise be failure cases (i.e., not recovered by the default template set). Each entry shows the number of such test reactions, the number of reference reactions in the training set (frequency), and the corresponding reaction SMARTS. Non-zero hydrogen counts are included for any atom for which they are explicitly specified in the reaction SMARTS.

CAS Template 6

- Number of recovered test set reactions that are not covered by the default template set: 99, Number of reference reactions in the training set: 2
- Reaction SMARTS: [C:1]-[O&H1&+0&D1:4].[C:2]-[O&H1&+0&D1:3]>>[C:1]-[O&H0&+0&D2:4]-C-C.[C:2]-[O&H0&+0&D2:3]-C-C

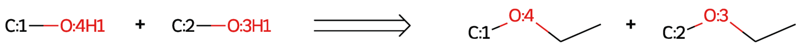

CAS Template 7

- Number of recovered test set reactions that are not covered by the default template set: 89, Number of reference reactions in the training set: 1
- Reaction SMARTS:

[n&H0&+0&D2:1]1:[c&H0&+0&D3:7](:[c&H0&+0&D3:9](:[c:2]):[c:10]:[n&H0&+0&D2:8]:[c&H0&+0&D3:11]:1-[c&H0&+0&D3:13](:[c:3]):[c:4])-[c&H0&+0&D3:12](:[c:5]):[c:6]
>>[N&H1&+0&D1:1]=[C&H0&+0&D3:11](-[N&H1&+0&D2:8]-[c:10]:[c&H1&+0&D2:9]:[c:2])-[c&H0&+0&D3:13](:[c:3]):[c:4].[c:5]:[c&H0&+0&D3:12](:[c:6])-[C&H1&+0&D2:7]=O

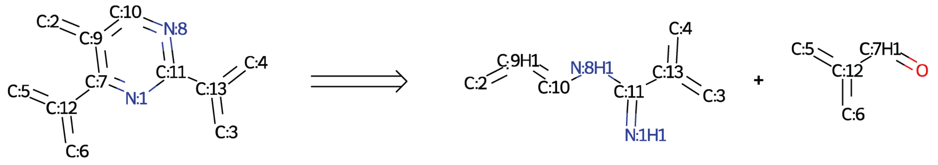

CAS Template 8

- Number of recovered test set reactions that are not covered by the default template set: 87, Number of reference reactions in the training set: 4
- Reaction SMARTS: [C:1]-[C&H0&+0&D3:7](-[N&H1&+0&D2:3]-[C:2])=[C:5]-[C:6]=[O&H0&D1:4]>>[C:1]-[C&H0&+0&D3:7](=[C:5]-[C:6]=[O&H0&D1:4])-O.[C:2]-[N&H2&+0&D1:3]

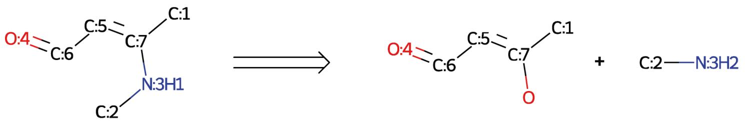

CAS Template 9

- Number of recovered test set reactions that are not covered by the default template set: 87, Number of reference reactions in the training set: 2
- Reaction SMARTS: [#8:1]-[C:7](=[O&H0&D1:3])-[C&H2&+0&D2:6]-[C&H0&+0&D4:8](-[O&H1&+0&D1:2])(-[c:4])-[c:5]
>>[#8:1]-[C:7](=[O&H0&D1:3])-[C&H2&+0&D2:6]-Br.[O&H0&+0&D1:2]=[C&H0&+0&D3:8](-[c:4])-[c:5]

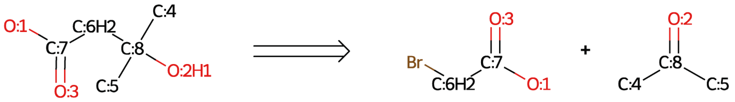

CAS Template 10

- Number of recovered test set reactions that are not covered by the default template set: 69, Number of reference reactions in the training set: 1
- Reaction SMARTS: [O&H1&+0&D1:1]-[C&H0&+0&D3:6](-[c:4])=[O&H0&+0&D1].[O&H1&+0&D1:2]-[C&H0&+0&D3:5](-[c:3])=[O&H0&+0&D1]
>>[O&H0&+0&D1:1]=[C&H0&+0&D3:6](-[c:4])-O-C-C.[O&H0&+0&D1:2]=[C&H0&+0&D3:5](-[c:3])-O-C-C

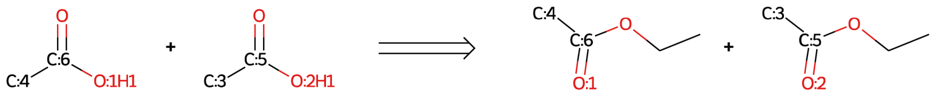

Figure S29 Filtered-out CAS templates ranked six through ten by the number of test reactions recovered, with the same information reported as in Fig. S28.

S5.7.2 Unique precursors generated per product molecule

We compute the number of unique precursors generated for each product molecule by exhaustively enumerating all templates. For computational efficiency, we randomly subsample 10,000 unique products from the test set for each dataset.

Table S13 Template enumeration statistics for a random sample of 10,000 product molecules per dataset. We report (1) the total number of templates in the set, (2) the number of templates that successfully apply to each product molecule (i.e., give at least one precursor), and (3) the number of unique precursor sets generated for each product molecule.

			USPTO	Pistachio	CAS
**Default Templates** (freq $\geq$ 5)	Total Number of Templates		16,495	53,275	91,589
	Number of Successfully Applied Templates	Mean $\pm$ SD	189 $\pm$ 83	443 $\pm$ 222	411 $\pm$ 215
		Min – Max	0 – 578	2 – 2,253	0 – 1,751
	Number of Unique Precursor Sets	Mean $\pm$ SD	263 $\pm$ 135	668 $\pm$ 614	844 $\pm$ 1,376
		Min – Max	0 – 2,509	2 – 24,996	0 – 53,484
**All Templates** (no freq cutoff)	Total Number of Templates		219,171	655,247	647,234
	Number of Successfully Applied Templates	Mean $\pm$ SD	1,344 $\pm$ 555	3,220 $\pm$ 1,602	1,904 $\pm$ 976
		Min – Max	8 – 3,731	8 – 15,334	7 – 8,999
	Number of Unique Precursor Sets	Mean $\pm$ SD	2,563 $\pm$ 1691	6,834 $\pm$ 6,237	4,684 $\pm$ 9,270
		Min – Max	9 – 26,887	16 – 188,036	7 – 390,812

S5.7.3 Template-based model performance stratified by complexity

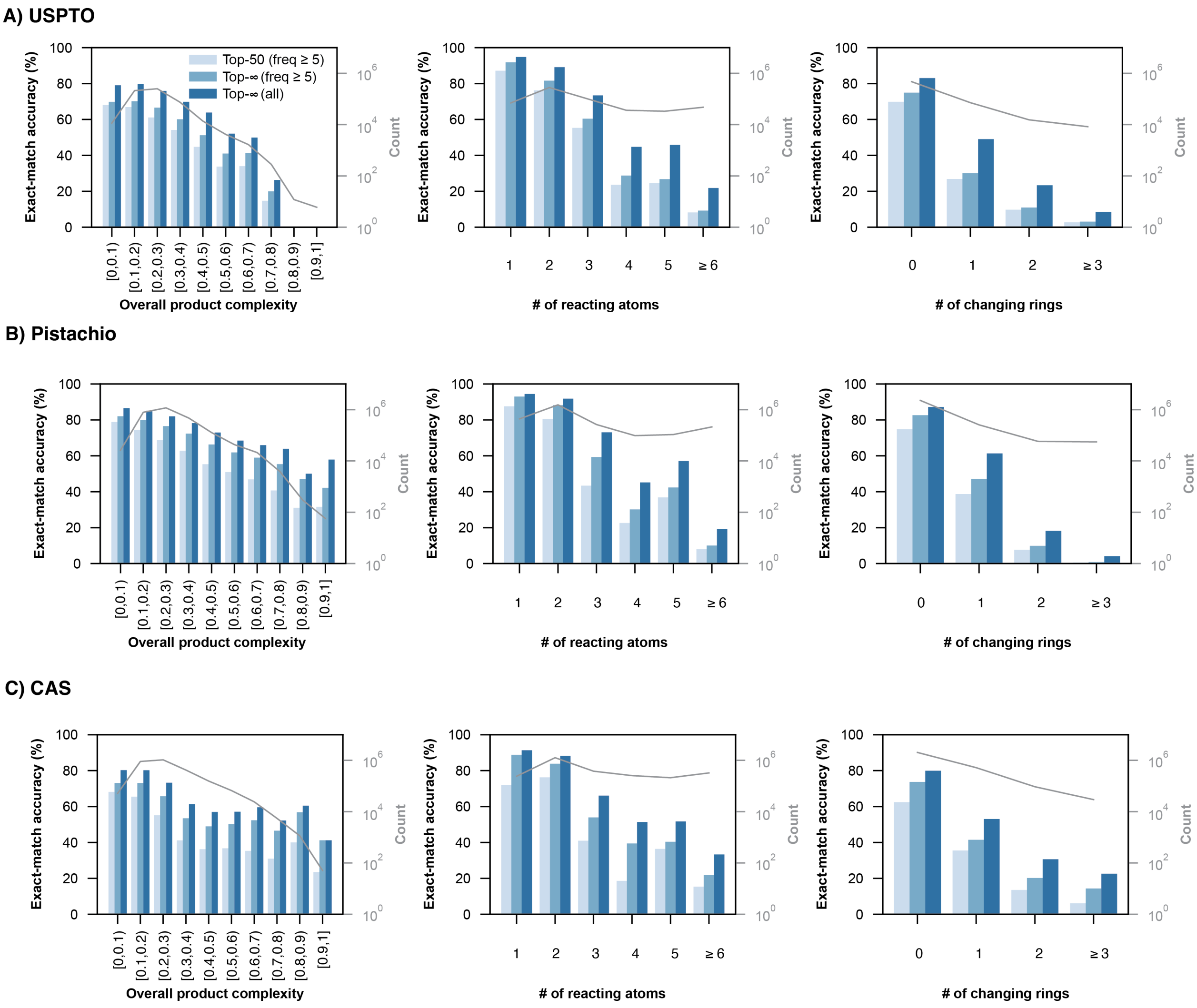

Figure S30 Exact-match accuracy of template-based models across three datasets, evaluated for varying top-k predictions, using either templates with a minimum frequency of 5 or all templates extracted from the training set A) USPTO, B) Pistachio, and C) CAS

S5.8 Summary of complementary metrics for all models and three datasets

Here, we report the percentage of test reactions for which predictions from at least one model satisfy each accuracy metric or combination of metrics.

Table S14 Summary of failure cases across datasets. Accuracies reflect the percentage of test reactions where predictions from at least one model satisfy at least one of the metrics.

Models	Accuracy metric	Top-*k* predictions	Accuracy (%)		
			USPTO	Pistachio	CAS
TR, NeuralLoc, AT, G2S, R-SMILES 2	exact-match	50	82.53	86.06	85.17
TR, NeuralLoc, AT, G2S, R-SMILES 2	stereochemistry-agnostic	50	83.21	87.27	86.86
TR, NeuralLoc, AT, G2S, R-SMILES 2	synthon	50	91.30	92.00	92.67
TR, NeuralLoc, AT, G2S, R-SMILES 2	superset	50	82.66	86.21	85.56
TR, NeuralLoc, AT, G2S, R-SMILES 2	two-step superset	5	78.30	82.48	83.00
–	**All 5 metrics and models**	–	92.34	93.06	94.44
TR, NeuralLoc, AT, G2S, R-SMILES 2	synthon + stereochemistry-agnostic	–	91.61	92.47	93.33
TR, NeuralLoc, AT, G2S, R-SMILES 2	stereochemistry-agnostic + two-step superset	–	85.50	88.97	89.79
TR, NeuralLoc, AT, G2S, R-SMILES 2	synthon + superset	–	91.30	92.00	92.67
TR, NeuralLoc, AT, G2S, R-SMILES 2	stereochemistry-agnostic + superset	–	83.34	87.39	87.24
TR, NeuralLoc, AT, G2S, R-SMILES 2	synthon + two-step superset	–	92.08	92.65	93.87
TR, NeuralLoc, AT, G2S, R-SMILES 2	two-step superset + superset	–	84.95	88.05	88.48
TR	exact-match	∞ (freq $\geq$ 5)	66.46	75.92	64.88
TR	exact-match	∞ (all)	76.03	81.47	72.33
–	**All metrics and models**	–	92.65	93.94	95.05

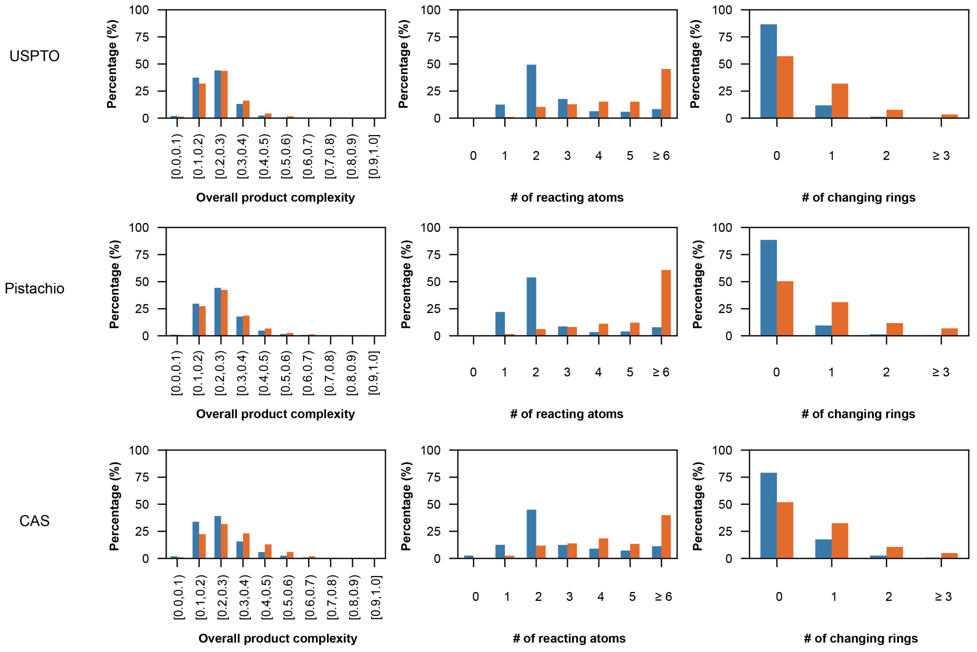

Figure S31 Distribution of product complexity and reaction complexity metrics for the test set (blue) and for the subset of test reactions where model predictions cannot be explained by any of the five complementary metrics (orange).

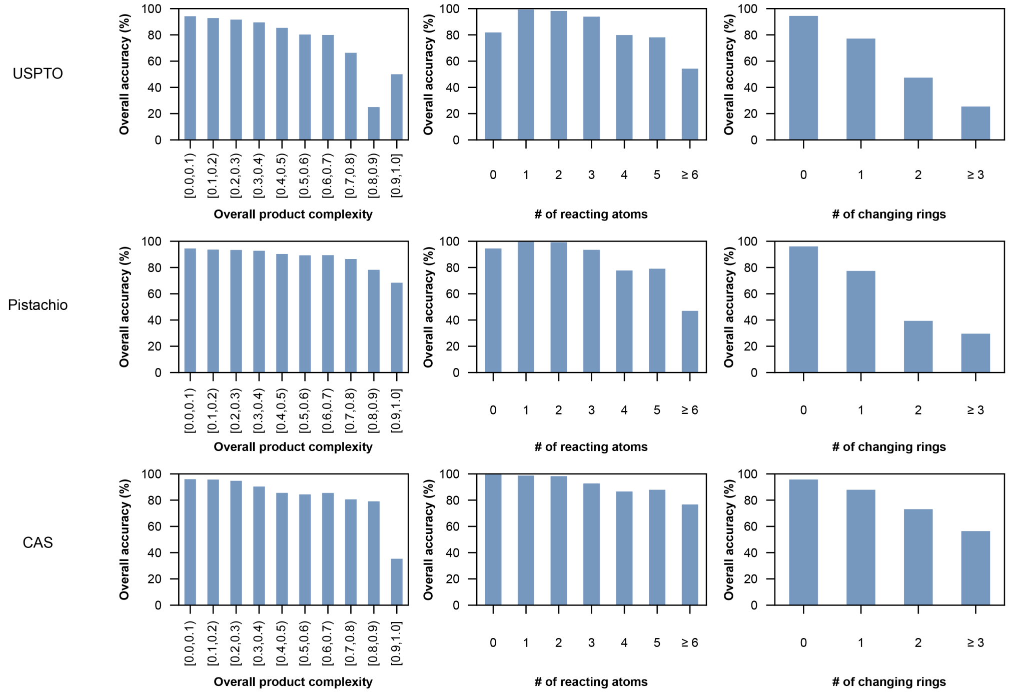

Figure S32 Combined overall accuracy stratified by product and reaction complexity. Overall accuracy is aggregated across all evaluation metrics and models.

Table S15 Summary of failure cases of Template-relevance model across datasets.

Accuracy metric	Top-*k* predictions	Accuracy (%)		
		**USPTO**	**Pistachio**	**CAS**
exact-match	50	61.45	67.24	53.63
stereochemistry-agnostic	50	62.61	69.36	55.95
synthon	50	73.20	77.14	63.53
superset	50	61.54	68.28	53.77
two-step superset	5	55.01	64.28	55.60
**All 5 metrics**	–	**76.01**	**81.22**	**71.13**
synthon + stereochemistry-agnostic	–	74.24	78.98	65.52
synthon + two-step superset	–	74.97	79.40	69.17
stereochemistry-agnostic + two-step superset	–	66.98	75.79	64.89
stereochemistry-agnostic + superset	–	62.70	70.40	56.08
synthon + superset	–	73.20	77.14	63.53
two-step superset + superset	–	65.83	73.83	62.66
exact-match	∞ (freq $\geq$ 5)	66.46	75.92	64.88
exact-match	∞ (all)	76.03	81.47	72.33
**All metrics**	–	**83.09**	**88.31**	**80.10**

Table S16 Summary of failure cases of NeuralLoc model across datasets.

Accuracy metric	Top-*k* predictions	Accuracy (%)		
		**USPTO**	**Pistachio**	**CAS**
exact-match	50	68.39	68.51	56.11
stereochemistry-agnostic	50	69.78	70.72	58.52
synthon	50	79.12	77.33	65.91
superset	50	68.47	69.56	56.28
two-step superset	5	58.52	62.83	53.38
**All 5 metrics**	–	**81.46**	**80.38**	**70.64**
synthon + stereochemistry-agnostic	–	80.25	79.20	67.97
synthon + two-step superset	–	80.35	78.52	68.61
stereochemistry-agnostic + two-step superset	–	72.59	74.55	63.36
stereochemistry-agnostic + superset	–	69.85	71.77	58.69
synthon + superset	–	79.12	77.33	65.91
two-step superset + superset	–	71.27	72.53	61.02

Table S17 Summary of failure cases of Augmented Transformer model across datasets.

Accuracy metric	Top-*k* predictions	Accuracy (%)		
		**USPTO**	**Pistachio**	**CAS**
exact-match	50	70.55	72.73	72.36
stereochemistry-agnostic	50	71.34	74.18	73.98
synthon	50	84.98	85.70	85.11
superset	50	70.68	72.86	72.67
two-step superset	5	63.73	65.13	66.79
**All 5 metrics**	–	**86.28**	**87.20**	**87.15**
synthon + stereochemistry-agnostic	–	85.43	86.47	85.92
synthon + two-step superset	–	85.87	86.48	86.42
stereochemistry-agnostic + two-step superset	–	73.67	76.48	76.90
stereochemistry-agnostic + superset	–	71.47	74.30	74.28
synthon + superset	–	84.98	85.70	85.11
two-step superset + superset	–	72.98	75.19	75.49

Table S18 Summary of failure cases of Graph2SMILES model across datasets.

Accuracy metric	Top-k predictions	Accuracy (%)		
		USPTO	Pistachio	CAS
exact-match	50	70.45	76.81	74.27
stereochemistry-agnostic	50	71.69	78.39	76.18
synthon	50	83.31	87.02	84.91
superset	50	70.68	76.92	74.58
two-step superset	5	63.80	69.82	68.35
**All 5 metrics**	–	**85.06**	**88.60**	**87.12**
synthon + stereochemistry-agnostic	–	84.10	87.85	85.88
synthon + two-step superset	–	84.33	87.84	86.24
stereochemistry-agnostic + two-step superset	–	74.11	80.49	78.92
stereochemistry-agnostic + superset	–	71.91	78.49	76.48
synthon + superset	–	83.31	87.02	84.91
two-step superset + superset	–	73.06	79.10	77.29

Table S19 Summary of failure cases of R-SMILES 2 model across datasets.

Accuracy metric	Top-k predictions	Accuracy (%)		
		USPTO	Pistachio	CAS
exact-match	50	77.95	76.67	70.48
stereochemistry-agnostic	50	78.57	77.43	71.63
synthon	50	87.53	88.47	81.64
superset	50	78.03	82.46	73.87
two-step superset	5	68.68	74.23	65.36
**All 5 metrics**	–	**88.63**	**89.65**	**84.22**
synthon + stereochemistry-agnostic	–	87.92	88.94	82.30
synthon + two-step superset	–	88.29	89.22	83.62
stereochemistry-agnostic + two-step superset	–	80.40	83.81	76.85
stereochemistry-agnostic + superset	–	78.65	83.22	75.00
synthon + superset	–	87.53	88.47	81.64
two-step superset + superset	–	79.87	83.93	76.84

S5.9 Additional reaction examples

1) We retrieved from the training set the most similar cases to the reaction example in Fig. 2C. To compute the product Tanimoto similarity, Morgan fingerprints (radius = 2 and 2048 bits) were generated from the product SMILES. The reaction fingerprint was then obtained by subtracting the reactant fingerprint from the product fingerprint, and the Tanimoto similarity was calculated between the resulting fingerprints.

A

SciFinder reaction ID:	31-614-CAS-26874882
Böttcher Score:	459.28
SAScore:	4.24
NPScore:	- 0.31
SPS:	28.31
# of reacting atoms:	6
# of changing rings:	3

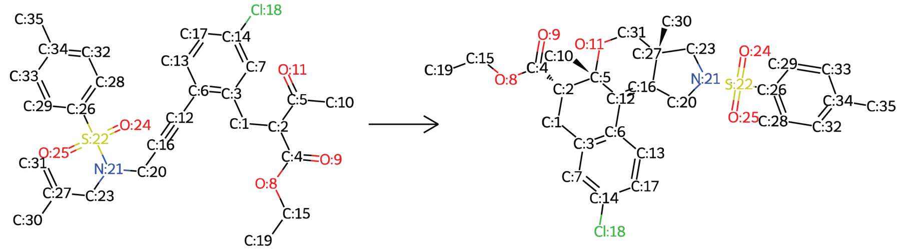

B

SciFinder reaction ID:	31-295-CAS-14712009
Böttcher Score:	289.81
SAScore:	3.72
NPScore:	-0.22
SPS:	27.28
# of reacting atoms:	6
# of changing rings:	2
Product similarity:	0.41
Reaction similiarity:	0.27

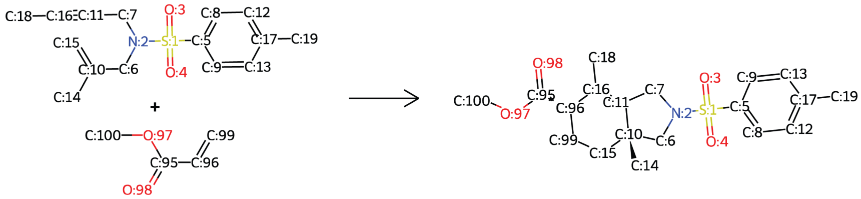

C

SciFinder reaction ID:	31-289-CAS-13524515
Böttcher Score:	331.81
SAScore:	5.25
NPScore:	0.15
SPS:	33.6
# of reacting atoms:	8
# of changing rings:	3
Product similarity:	0.31
Reaction similiarity:	0.34

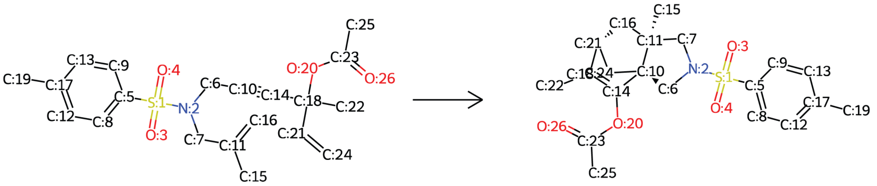

Figure S33 A) the reaction example in Fig. 2C with atom-mapping. B and C) Retrieved reactions with closest product (B) and closest reaction (C) to the example in (A).

2) Examples that G2S underpredicts number of reacting atoms and changing rings Models tend to underpredict both number of reacting atoms and changing rings. Fig. S34A illustrates an example where the model correctly identifies the number of reacting atoms but underpredict the number of changing rings. In another example, where the reaction involves the formation of an *N*-tosyl-α-chloroaldimine followed by nucleophilic attack by a *p*-toluenesulfonyl anion[31], G2S not only underpredicts the number of reacting atoms but also identifies the wrong atoms (Fig. S34B).

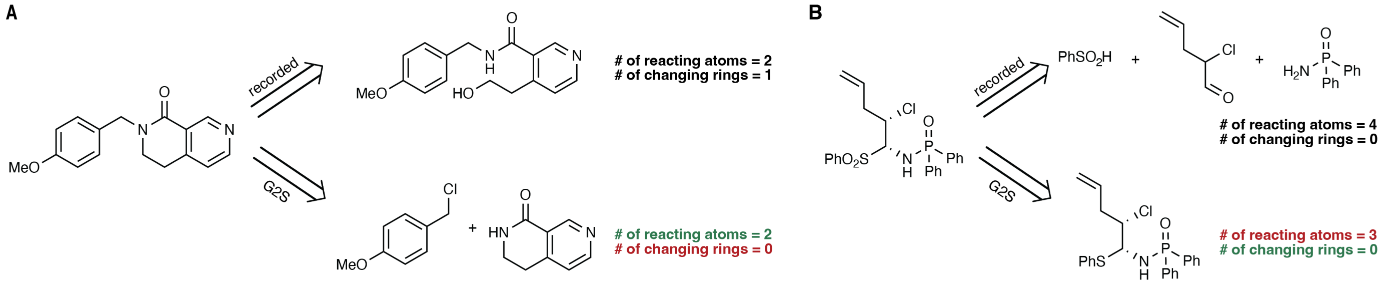

Figure S34 Examples in CAS that G2S underpredicts A) the number of reacting atoms[31] and B) the number of changing rings[32]. In both cases, only the top-1 candidate is shown, and no correct prediction is found within the top-50.

3) Examples in CAS that are not able captured by any of the complementary accuracy metrics and models.

Product

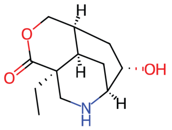

Recorded precursor(s)

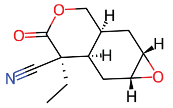

SciFinder reaction ID:	31-035-CAS-9756845
Overall product complexity:	0.47
Böttcher Score:	267.74
SAScore:	5.53
NPScore:	2.43
SPS:	51.00
# of reacting atoms:	4
# of changing rings:	2

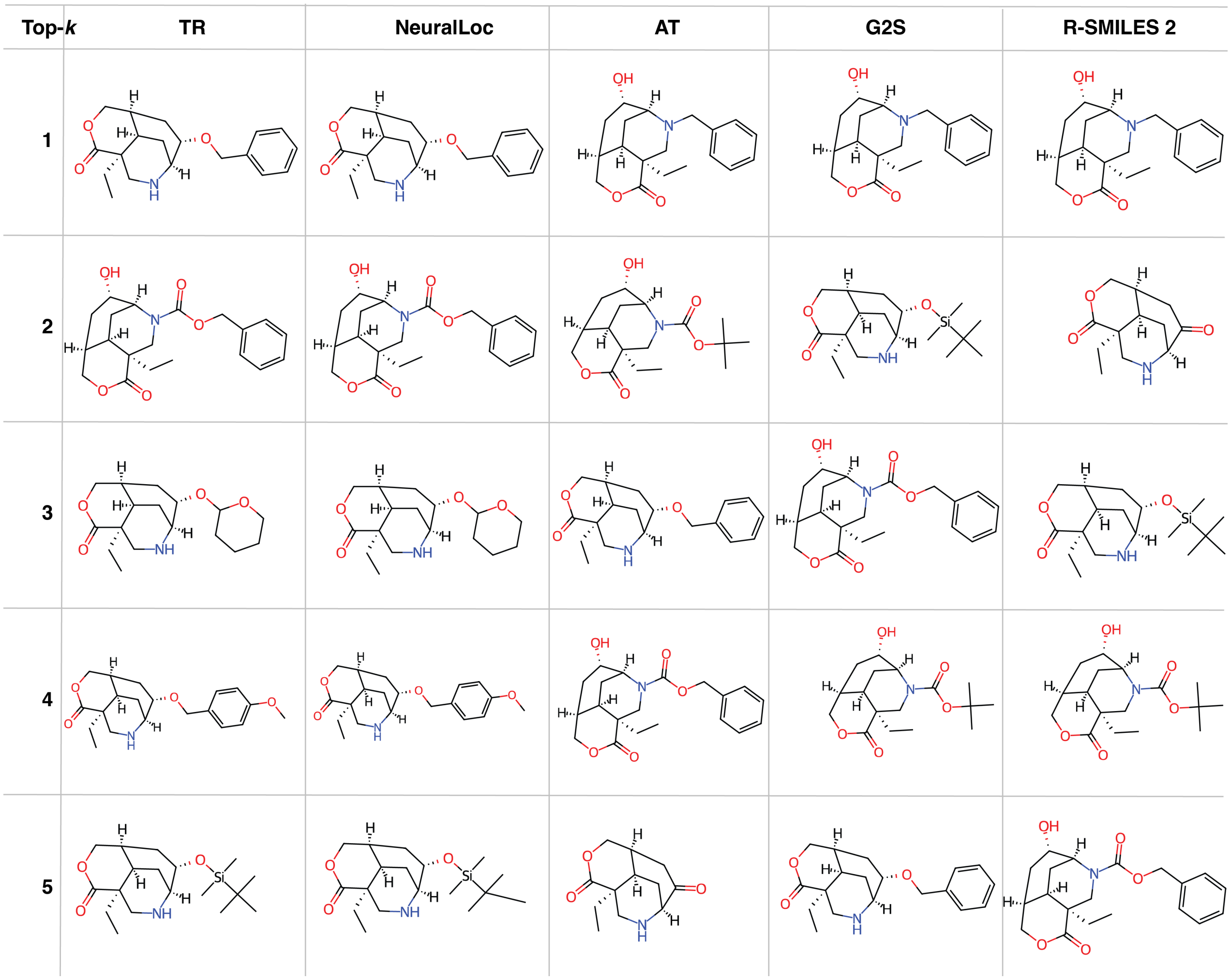

Figure S35 Example in CAS not captured by any of the complementary accuracy metrics. This is a multi-stage reaction, where the nitrile is first reduced to an amine, followed by the opening of the epoxide ring.

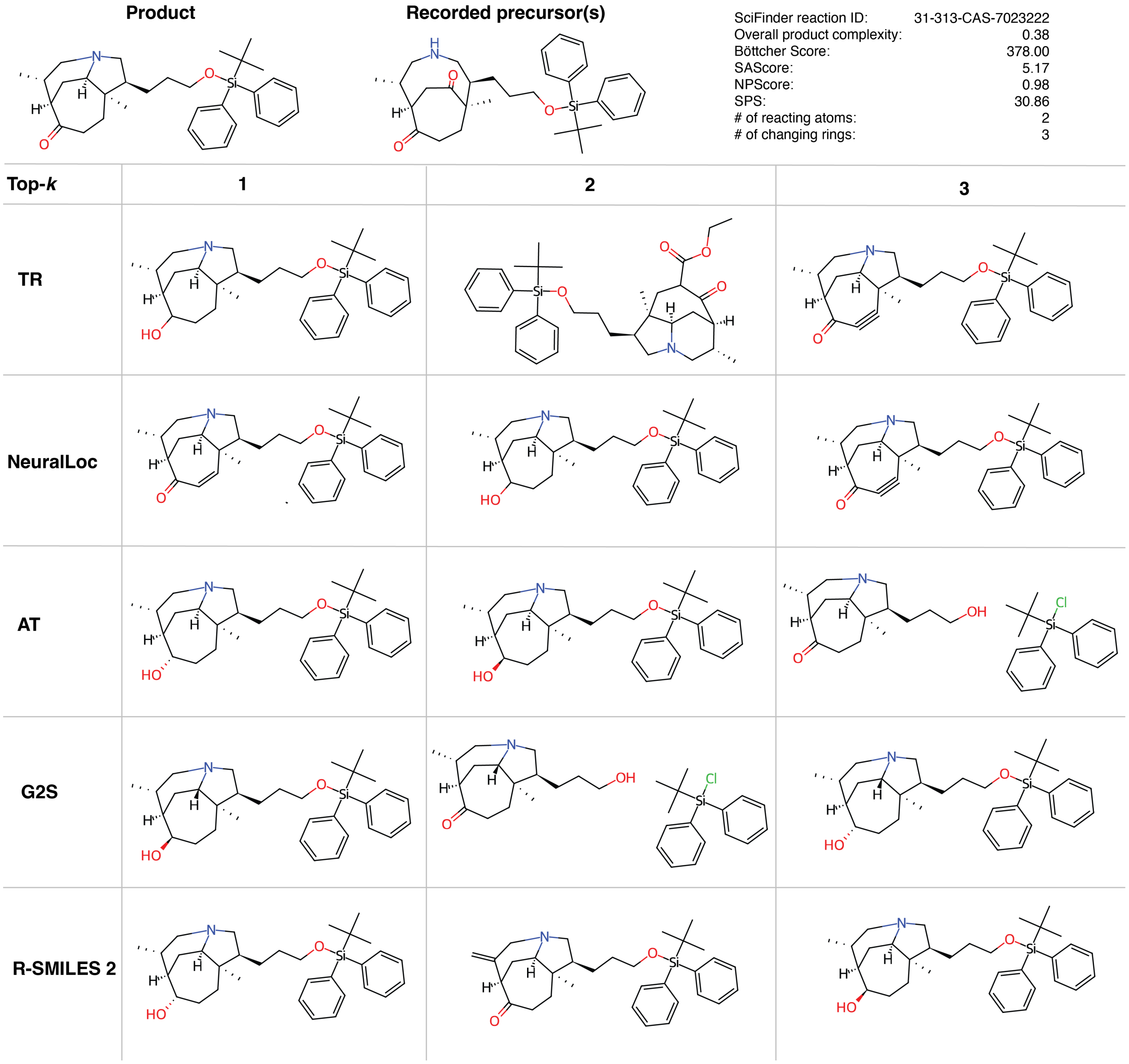

Figure S36 Example of failure cases from CAS not captured by any of the complementary accuracy metrics. The reaction involves reductive amination, with only two reacting atoms but three changing rings. In most cases, the model underpredicts the number of changing rings, leading to inaccurate precursor predictions.

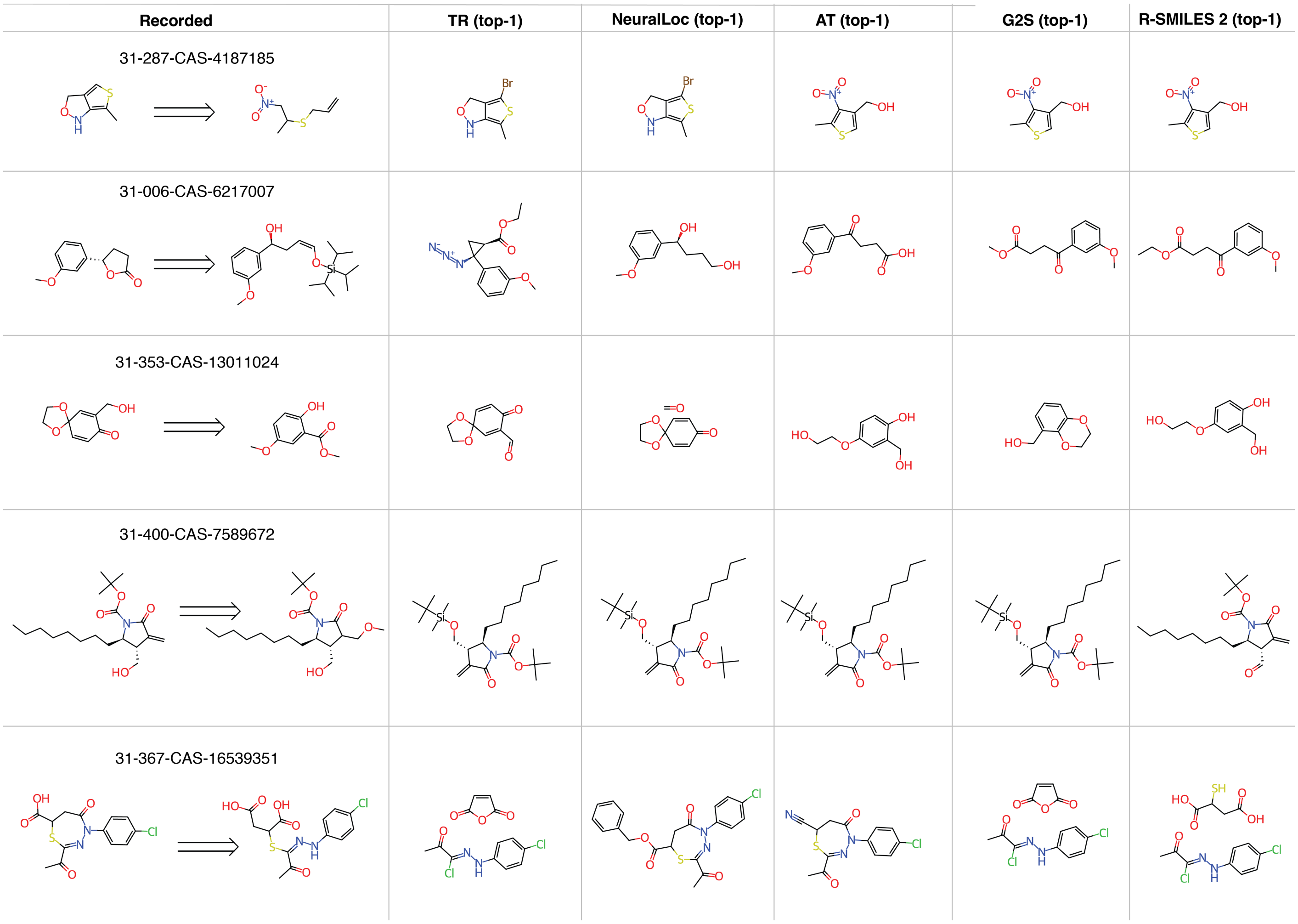

Figure S37 Example of failure cases from CAS not captured by any of the complementary accuracy metrics.

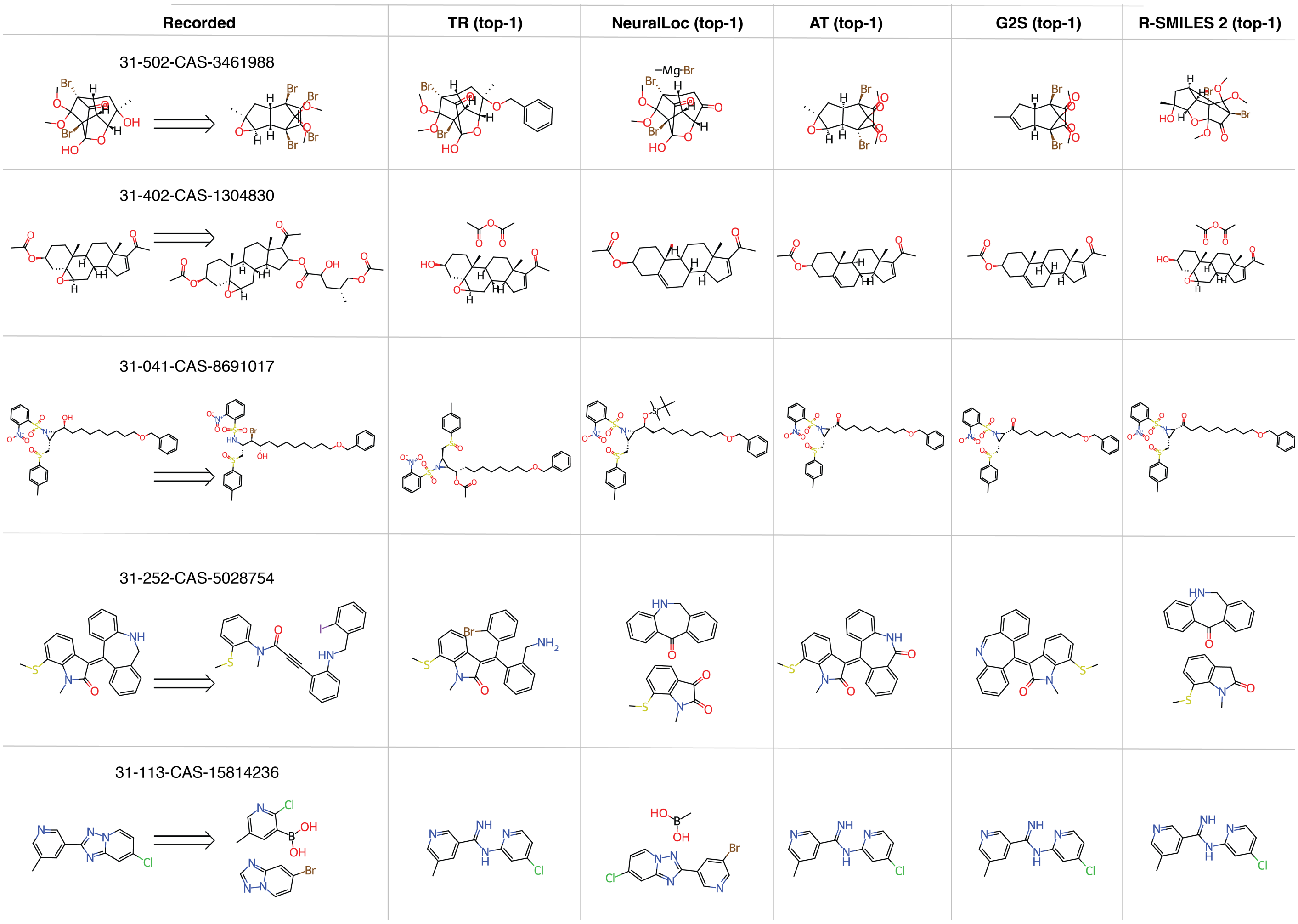

Figure S38 Example of failure cases from CAS not captured by any of the complementary accuracy metrics.

Notes and references

[1] Z. Tu, S. J. Choure, M. H. Fong, J. Roh, I. Levin, K. Yu, J. F. Joung, N. Morgan, S.-C. Li, X. Sun, H. Lin, M. Murnin, J. P. Liles, T. J. Struble, M. E. Fortunato, M. Liu, W. H. Green, K. F. Jensen and C. W. Coley, *Acc. Chem. Res.*, 2025, **58**, 1764–1775.

[2] T. Bottcher, *J. Chem. Inf. Model.*, 2016, **56**, 462–470.

[3] A. Krzyzanowski, A. Pahl, M. Grigalunas and H. Waldmann, *J. Med. Chem.*, 2023, **66**, 12739–12750.

[4] P. Ertl and A. Schuffenhauer, *J Cheminform*, 2009, **1**, 8.

[5] P. Ertl, S. Roggo and A. Schuffenhauer, *J. Chem. Inf. Model.*, 2008, **48**, 68–74.

[6] *Chemical reactions from US patents (1976-Sep2016)*, 2017, `https://figshare.com/articles/dataset/Chemical_reactions_from_US_patents_1976-Sep2016_/5104873/1`.

[7] *NextMove Software | Pistachio*, `https://www.nextmovesoftware.com/pistachio.html`.

[8] *CAS Databases*, `https://www.cas.org/training/documentation/cas-databases`.

[9] C. W. Coley, W. H. Green and K. F. Jensen, *J. Chem. Inf. Model.*, 2019, **59**, 2529–2537.

[10] D. Weininger, *J. Chem. Inf. Comput. Sci.*, 1988, **28**, 31–36.

[11] N. Schneider, D. M. Lowe, R. A. Sayle and G. A. Landrum, *J. Chem. Inf. Model.*, 2015, **55**, 39–53.

[12] I. V. Tetko, P. Karpov, R. Van Deursen and G. Godin, *Nat. Commun.*, 2020, **11**, 5575.

[13] T. Y. Cowie, L. Kennedy, J. M. Åżurek, M. J. Paterson and M. W. P. Bebbington, *J. European Organic Chemistry*, 2015, **2015**, 3818–3823.

[14] J. Zhang, C. Yu, S. Wang, C. Wan and Z. Wang, *Chem. Commun.*, 2010, **46**, 5244–5246.

[15] B. F. Mirjalili and M. A. Mirhoseini, *J Chem Sci*, 2013, **125**, 1481–1486.

[16] J. L. Hodges, *Ark. Mat.*, 1958, **3**, 469–486.

[17] P. Virtanen, R. Gommers, T. E. Oliphant, M. Haberland, T. Reddy, D. Cournapeau, E. Burovski, P. Peterson, W. Weckesser, J. Bright, S. J. van der Walt, M. Brett, J. Wilson, K. J. Millman, N. Mayorov, A. R. J. Nelson, E. Jones, R. Kern, E. Larson, C. J. Carey, Ä. Polat, Y. Feng, E. W. Moore, J. VanderPlas, D. Laxalde, J. Perktold, R. Cimrman, I. Henriksen, E. A. Quintero, C. R. Harris, A. M. Archibald, A. H. Ribeiro, F. Pedregosa and P. van Mulbregt, *Nat Methods*, 2020, **17**, 261–272.

[18] J. Roh, J. F. Joung, K. Yu, Z. Tu, G. L. Bartholomew, O. A. Santiago-Reyes, M. H. Fong, R. Sarpong, S. E. Reisman and C. W. Coley, *Higher-level Strategies for Computer-Aided Retrosynthesis*, 2025, `https://chemrxiv.org/engage/chemrxiv/article-details/67a367196dde43c908af44a1`.

[19] K. Yu, J. Roh, R. Wang, Z. Li, W. Gao and C. W. Coley, 2024.

[20] L. Biewald, *Experiment Tracking with Weights and Biases*, 2020, `https://www.wandb.com/`.

[21] V. R. Somnath, C. Bunne, C. W. Coley, A. Krause and R. Barzilay, *Learning Graph Models for Retrosynthesis Prediction*, 2021, `http://arxiv.org/abs/2006.07038`, arXiv:2006.07038 [cs].

[22] C. Shi, M. Xu, H. Guo, M. Zhang and J. Tang, author, 2020, pp. 8818–8827.

[23] Z. Chen, O. R. Ayinde, J. R. Fuchs, H. Sun and X. Ning, *Commun Chem*, 2023, **6**, 102.

[24] N. Laabid, S. Rissanen, M. Heinonen, A. Solin and V. Garg.

[25] I. Igashov, A. Schneuing, M. Segler, M. Bronstein and B. Correia, The Twelfth International Conference on Learning Representations, 2024.

[26] Y. Wang, Y. Song, Y. Wang, M. Xu, R. Wang, H. Zhou and W.-Y. Ma, *RetroDiff: Retrosynthesis as Multi-stage Distribution Interpolation*, 2025, `http://arxiv.org/abs/2311.14077`, arXiv:2311.14077 [cs].

[27] R. Yadav, Q. Yan, G. Wolf, A. J. Bose and R. Liao, *RETRO SYNFLOW: Discrete Flow Matching for Accurate and Diverse Single-Step Retrosynthesis*, 2025, `http://arxiv.org/abs/2506.04439`, arXiv:2506.04439 [cs].

[28] N. Schneider, N. Stiefl and G. A. Landrum, *J. Chem. Inf. Model.*, 2016, **56**, 2336–2346.

[29] P. Schwaller, B. Hoover, J.-L. Reymond, H. Strobelt and T. Laino, *Science Advances*, 2021, **7**, eabe4166.

[30] W. Jaworski, S. Szymkuć, B. Mikulak-Klucznik, K. Piecuch, T. Klucznik, M. Kaźmierowski, J. Rydzewski, A. Gambin and B. A. Grzybowski, *Nature communications*, 2019, **10**, 1434.

[31] G. R. Stanton, M. GÃűllü, R. M. Platoff, C. E. Rich, P. J. Carroll and P. J. Walsh, *Advanced Synthesis & Catalysis*, 2013, **355**, 757–764.

[32] M. G. Bock, C. Gaul, V. R. Gummadi, H. Moebitz and S. Sengupta, 2012.
